# Presence of a classical RRM-fold palm domain in Thg1-type 3'- 5'nucleic acid polymerases and the origin of the GGDEF and CRISPR polymerase domains

**DOI:** 10.1186/1745-6150-5-43

**Published:** 2010-06-30

**Authors:** Vivek Anantharaman, Lakshminarayan M Iyer, L Aravind

**Affiliations:** 1 National Center for Biotechnology Information, National Library of Medicine, National Institutes of Health, Bethesda, MD 20894, USA

## Abstract

**Background:**

Almost all known nucleic acid polymerases catalyze 5'-3' polymerization by mediating the attack on an incoming nucleotide 5' triphosphate by the 3'OH from the growing polynucleotide chain in a template dependent or independent manner. The only known exception to this rule is the Thg1 RNA polymerase that catalyzes 3'-5' polymerization *in vitro *and also *in vivo *as a part of the maturation process of histidinyl tRNA. While the initial reaction catalyzed by Thg1 has been compared to adenylation catalyzed by the aminoacyl tRNA synthetases, the evolutionary relationships of Thg1 and the actual nature of the polymerase reaction catalyzed by it remain unclear.

**Results:**

Using sensitive profile-profile comparison and structure prediction methods we show that the catalytic domain Thg1 contains a RRM (ferredoxin) fold palm domain, just like the viral RNA-dependent RNA polymerases, reverse transcriptases, family A and B DNA polymerases, adenylyl cyclases, diguanylate cyclases (GGDEF domain) and the predicted polymerase of the CRISPR system. We show just as in these polymerases, Thg1 possesses an active site with three acidic residues that chelate Mg^++ ^cations. Based on this we predict that Thg1 catalyzes polymerization similarly to the 5'-3' polymerases, but uses the incoming 3' OH to attack the 5' triphosphate generated at the end of the elongating polynucleotide. In addition we identify a distinct set of residues unique to Thg1 that we predict as comprising a second active site, which catalyzes the initial adenylation reaction to prime 3'-5' polymerization. Based on contextual information from conserved gene neighborhoods we show that Thg1 might function in conjunction with a polynucleotide kinase that generates an initial 5' phosphate substrate for it at the end of a RNA molecule. In addition to histidinyl tRNA maturation, Thg1 might have other RNA repair roles in representatives from all the three superkingdoms of life as well as certain large DNA viruses. We also present evidence that among the polymerase-like domains Thg1 is most closely related to the catalytic domains of the GGDEF and CRISPR polymerase proteins.

**Conclusion:**

Based on this relationship and the phyletic patterns of these enzymes we infer that the Thg1 protein is likely to represent an archaeo-eukaryotic branch of the same clade of proteins that gave rise to the mobile CRISPR polymerases and in bacteria spawned the GGDEF domains. Thg1 is likely to be close to the ancestral version of this family of enzymes that might have played a role in RNA repair in the last universal common ancestor.

**Reviewers:**

This article was reviewed by S. Balaji and V.V. Dolja.

## Findings

Nucleic acid polymerase activity has emerged independently in at least four structurally unrelated folds, namely the RNA-recognition motif (RRM) fold (also known as ferredoxin fold in the SCOP database), the double ψ-beta barrel, the polβ-like and TOPRIM folds [1-3]. Yet, all of these polymerases are only known to catalyze 5'-3' chain elongation by adding a nucleotide phosphate derived from a 5' nucleotide triphosphate substrate to the 3' OH of the prior nucleotide. This reaction might occur in a nucleic acid template-dependent or template-independent manner based on the polymerase in question. However, in the past few years a notable exception to this has emerged in form of the histidinyl tRNA guanylyl transferase (Thg1), which is highly conserved in eukaryotes, and also found in several archaea and more sporadically in bacteria [4]. This enzyme was originally characterized as synthesizing the guanine nucleotide at the -1 position of the histidinyl tRNA (HtRNA). HtRNA is distinct from all other tRNAs in possessing a unique extension of G-1 at the 5' end that is complementary to the position 73 at the 3' end, just upstream of the terminal CCA triplet. In most bacteria, the G-1 is genomically encoded and left in place due to exceptional processing by the RNAse P ribozyme [5]. However, in eukaryotes and several archaea this G-1 is added by the Thg1 [4]. In eukaryotes the reaction proceeds in a template-independent fashion, as the position 73 nucleotide in HtRNA is an adenine. However, the archaeal HtRNA possesses a cytosine and the archaeal Thg1 elongates the 5' end in a template-dependent manner [6]. Furthermore, this 3'-5' template-dependent polymerization has also been demonstrated for the yeast enzyme when supplied with the appropriate cytosine-containing templates [7]. The Thg1 polymerization reaction has been shown to be processive in terms of being able to add further nucleotides in the 3'-5' direction, if appropriate templates are supplied. Accordingly, it has been proposed that this polymerase activity is likely to have been the ancestral activity of the Thg1 enzymes across the superkingdoms of life [6].

In the case of the template-independent ligation of G-1 the reaction proceeds via an initial adenylation of the 5' phosphate by Thg1 resulting in a 5' App overhang [7]. This is then attacked by the 3'OH of GTP to release an AMP and add G at the -1 position. A similar ligation reaction can occur in the absence of ATP if the 5'end of the tRNA substrate contains a preexisting triphosphate terminal [4]. In the processive template-dependent polymerization reaction Thg1 uses the terminal 5' triphosphate generated by addition of the initial nucleotide to add further nucleotides via attack of this triphosphate by the 3'OH of the incoming nucleotide. Initially, the first step in the template independent addition of G-1 catalyzed by Thg1 was compared to the adenylation reactions catalyzed by the tRNA synthetases that result in release of AMP [7]. However, the template-dependent polymerization reaction closely resembles the conventional polymerization reaction, except that the driving energy is supplied by the nucleotide triphosphate anchored to the 5' end of the polynucleotide rather than from the free in-coming nucleotide as seen in conventional 5'-3' polymerization reactions. While site-directed mutagenesis has identified residues important for catalysis by Thg1, to date no relationship has been shown to any other known catalytic domain [8].

We were interested in the unusual catalytic properties of Thg1 and were intrigued by its mysterious evolutionary affinities. As a part of our effort to understand the origins of nucleic acid polymerization, we investigated Thg1 using sensitive sequence-profile methods and showed that it belongs to the RRM-fold nucleic acid polymerase palm domains. Furthermore, we show that it is most closely related to the catalytic domains of the diguanylate cyclases (GGDEF) and CRISPR system polymerases and provides new information regarding the origin and functions of these domains.

### Identification of a diguanylate cyclase-like catalytic domain in the Thg1 polymerases

**Figure 1:**
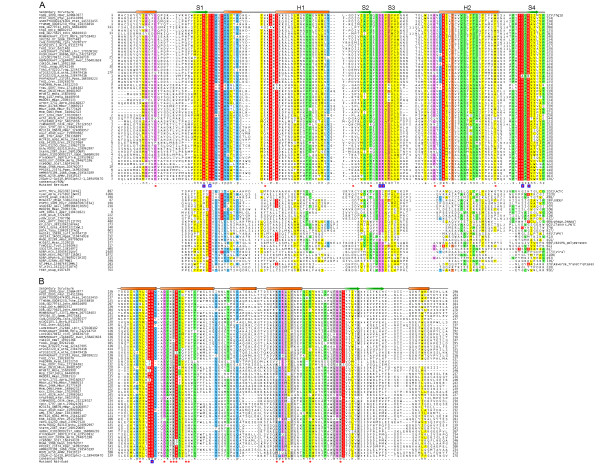
**Multiple alignment of Thg1 catalytic domain with other RRM-fold polymerase domains**. **A**. Multiple sequence alignment of the Thg1 catalytic domain was constructed using Kalign after parsing high-scoring pairs from PSI-BLAST search results. The alignment with the other RRM-fold polymerase plam domains was constructed based on the PSI-BLAST search results, pairwise alignments produced by the profile-profile searches with the HHpred program against the Thg1 catalytic domain, and DALI searches with the X-ray structures shown in the alignment (PDB codes). The secondary structure from the crystal structures is shown above the alignment with E representing a strand and H a helix. The 90% consensus shown below the alignment was derived for the Thg1 catalytic domains alone using the following amino acid classes: hydrophobic (h: ALICVMYFW, yellow shading); small (s: ACDGNPSTV, green); polar (p: CDEHKNQRST, blue) and its charged subset (c: DEHKR, pink), and big (b: FILMQRWYEK; grey shading). The limits of the domains are indicated by the residue positions, on each end of the sequence. The numbers within the alignment are non-conserved inserts that have not been shown. The sequences are denoted by their gene name followed by the species abbreviation and GenBank Identifier (gi). The active site residues are marked with a blue box. The mutated residues that affected Thg1 activity are shown below the alignment with orange circles. **B**. Multiple Alignment of the C terminal extension of the Thg1 domain. The multiple alignments of the C terminal extension of the Thg1 domain was constructed as described above. The abbreviations and legends are also as above.

**Figure 2:**
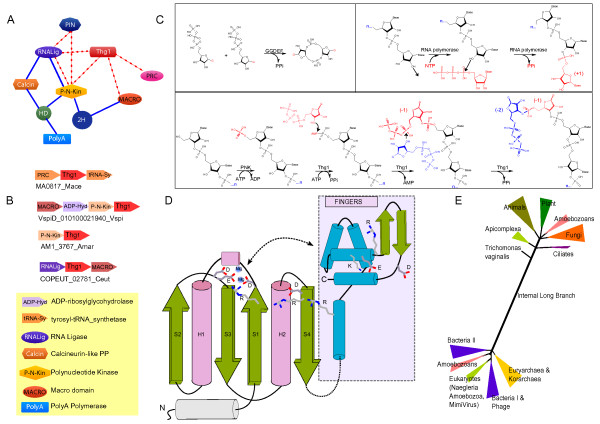
**Catalytic mechanism, topology of catalytic domain and contextual inference of Thg1 function**. **A**. Contextual Network of Thg1 domain with the blue edges indicating architectural contexts and the dotted red edges indicate predicted gene neighborhood contexts in prokaryotes. **B**. Examples of conserved gene neighborhoods of the prokaryotic Thg1 genes. The arrows indicate genes in an operon in the 5'-3' direction. The gene name Thg1 gene and organism from which the representative gene neighborhood has been extracted is shown below each representation. **C**. Simplified catalytic schemes for the GGDEF and RNA polymerase enzymes are shown along with Thg1 to illustrate the reaction differences. The Thg1 reaction scheme also includes the predicted reaction for the polynucleotide kinase (PNK) present in conserved gene neighborhoods with Thg1. Standard names are used for all enzymes. The "...n" is a placeholder for a polynucleotide chain. **D**. The topology of the Thg1 catalytic domain is shown as cartoon based on structure prediction and alignment with known RRM-fold polymerase palm domain structures. The conserved residues predicted to be required for catalysis are shown. The C terminal extension is also shown in the colored box. We predict that this extension will be able fold on the rest of the protein to be in proximity with the catalytic site and substrate. **E**. A phylogenetic tree of the Thg1 proteins was derived using the seed alignment similar to that shown in Fig. 1 with the FastTree program. Only the major phyletic groups supported with bootstrap values greater than 85% are shown. The thickness of a given branch is approximately proportional to the number of proteins contained within it.

To investigate the affinities of the Thg1 polymerase, we initiated sequence profile searches using the PSI-BLAST program [9] with several representative Thg1 proteins as seed sequences. In parallel we also initiated iterative hidden Markov model searches with the Jackhmmer program (For detailed Material and Methods refer to Additional File 1). These searches recovered sequences of Thg1 orthologs from diverse organisms belonging to the three superkingdoms of life. Additionally, at convergence in PSI-BLAST searches of the non-redundant/NR database, we also observed hits to GGDEF domains from different signaling proteins with marginal e-values. For example, a search initiated with *Pyrobaculum aerophilum *Thg1 recovered GGDEF proteins from *Hahella chejuensis *(gi: 83644929 e = .053 and *Thermosipho africanus *(gi: 217076806, e = .06). Although, these hits had only marginal significance, they perfectly aligned with the three principal acidic catalytic residues of the diguanylate cyclases/GGDEF domain (Fig. 1). Similarly, in a search with the JACKHMMER program using *Pyrobaculum aerophilum *Thg1 as a query against the NR database we recovered GGDEF domains (e.g. *Thermosipho africanus; *gi: 217076806) with conditional e-values as low as 6.1 × 10^-5^. We then predicted secondary structure using the Jpred program [10] with an alignment including all complete Thg1 sequences in the NR database and compared it to the crystal structure of the GGDEF domain (PDB: 3ign). The predicted secondary structure of Thg1 showed a conserved core of 4 strands and two helices (Fig. 1, 2) with absolutely conserved acidic residues at the end of the first strand and between strands 2 and 3 which form a hairpin. The location of these conserved residues in Thg1 with respect to the secondary structure corresponded precisely to the arrangement of the three acidic catalytic residues in the structure of the GGDEF domain. To further test this potential relationship, we used a HMM derived from the multiple alignment of the Thg1 proteins in profile-profile searches against two libraries of HMMs with the HHpred program [11]: 1) HMMs derived from the alignments in the PFAM database and 2) HMMs derived from searches seeded with representatives of structures in the PDB database. Both searches recovered the GGDEF domain as the best hit with significant p-values (p = 10^-7^-10^-6^). Additional profile-profile searches with an in-house group of profiles, which includes domains not properly represented in PFAM, recovered the catalytic domain of the polymerases of the CRISPR system [12]. This strongly suggested that the core conserved domain in Thg1 indeed adopts a GGDEF-like fold and is likely to contain a similar active site configuration with three acidic residues.

### Thg1 shares a unified active site and likely catalytic mechanism with other RRM-fold polymerase palm domains

The identification of a GGDEF-like catalytic domain in the Thg1 polymerases connects them to one of the largest group of nucleic acids polymerases, namely those that share a RRM-fold palm domain. The core group of these enzymes includes: 1) RNA-dependent RNA polymerases of positive strand RNA viruses and their derivatives (viral RdRP); 2) reverse transcriptases (RT); 3) DNA polymerase family B, including the archaeo-eukaryotic replicative enzymes; 4) the DinB-like DNA-repair polymerases; 5) DNA polymerase family A; 6) the phage T7-type DNA polymerases; 7) the phage T7-type DNA-dependent RNA polymerases; 8) the TV-polymerase family; 9) the CRISPR-system polymerases; 10) GGDEF domains; 11) nucleotide cyclases (cAMP and cGMP generating enzymes) [1,12]. The catalytic domains of all these enzymes are based on a RRM-like fold with 4 strands and 2 helices that forms a structure termed the palm domain. Furthermore, all the above enzymes are unified by a common set of acidic active site residues that are present at the end of strand 1 (nearly always an aspartate) and in the hairpin between strand 2 and 3 (usually a dyad of acidic residues; a single acidic residue in the nucleotide cyclases; Fig. 1) [12]. These residues together coordinate two Mg^2+ ^ions that are central to the catalytic mechanism of these enzymes. A common mechanism is deployed irrespective of whether an enzyme of this family catalyzes elongation of a polynucleotide chain or nucleotide cyclization (Fig. 2). The Mg^2+ ^ions direct the 3'OH of the nucleotide to attack the 5' triphosphate either on the same nucleotide (cNMP generation) or a second nucleotide (polymerization and cyclic diguanylate generation). A comparison of the Thg1 catalytic core with that of the palm domain from the above enzymes shows that it shares with the GGDEF, CRISPR, viral RdRP and RT the configuration of the active site at the hairpin, i.e. two acidic residues preceded by a small residue (Fig. 1). Consistent with the recovery of the GGDEF and CRISPR polymerase domains in searches with Thg1, they specifically share the absence of an insert between the strand 1 and helix 1 that is typical of all the other nucleic acid polymerases (Fig. 1). Thus, they are likely to share a closer common ancestor to the exclusion of other polymerase palm domains.

These observations suggest that 3'-5' polymerization catalyzed by the Thg1 polymerase domains is likely to depend on a Mg^2+^-dependent catalytic mechanism as in case of the other polymerase palm domains (Fig 2). However, in the case of Thg1, the 3' OH of the incoming nucleotide is directed by the cations to attack the 5' triphosphate present at the end of the elongating chain. Thus, in a sense this reaction is equivalent to one of the two nucleotide ligations performed by the GGDEF domain in the synthesis of cyclic diguanylate (Fig. 2). Further, like many of the CRISPR polymerases, Thg1 contains a highly conserved positively charged residue 2 positions upstream of the predicted catalytic aspartate in the first strand of the palm domain. Based on its location it is conceivable that it plays a role in interacting with the negatively charged 5' triphosphate at the end of the elongating chain. The Thg1 palm domains also possess a region of conservation at the N-terminus of strand 4 (typically DxR) that is absent in other palm domains. Based on its position it is predicted to flank the acidic residue on strand 1 on the side opposite to the Mg2+ chelating site. It is conceivable that these residues unique to Thg1 play an important role in the initial adenylation reaction required to prime polymerization. Consistent with our proposals, a subset of the above-discussed residues that were targeted in the site-directed mutagenesis study of yeast Thg1 resulted in inactive enzymes (Fig. 1) [8]. Most nucleic acid polymerases contain an insert (e.g. a helix-turn-helix domain) between strand 1 and helix 1 of the palm domain, which forms a module called the "fingers" (Fig. 1) [1]. The fingers might play a key role in holding the nucleic acid template; however, it appears to be absent in the Thg1 polymerases, just as in several CRISPR polymerases [12]. In contrast, Thg1 polymerases contain an additional region of conservation C-terminal to the core palm domain that appears to form a distinct module comprised of 5 helices and two strands. This region is characterized by several well-conserved charged residues (Fig. 1). In particular a basic residue found towards the end of the first helix of this unit might contribute to the Thg1-specific active site along with the above-described DxR motif (Fig. 2). Some of these were targeted in the mutagenesis study of yeast Thg1 and resulted in enzymes with significantly reduced catalytically activity [8]. Hence, we posit that this C-terminal module of Thg1 is likely to form a helical bundle that functions equivalently to the fingers of the other nucleic acid polymerases, probably in interacting with the template HtRNA (Fig. 2).

### Contextual information throws additional light on the functions of the Thg1 polymerases

The 3'-5' polymerization activity of Thg1, along with the presence of a genomically encoded -1 position in archaea and bacteria, raises questions regarding the actual function of this enzyme in these organisms. To investigate their potential functions we resorted to contextual information provided in the form of domain architectures and gene neighborhoods, which has proven to be a powerful tool in dissecting functions of uncharacterized proteins [13]. In terms of domain architectures, majority of the Thg1 proteins are standalone versions. The Thg1 proteins from seed plants display a duplication of the catalytic domain. While both copies are predicted to be catalytically active, only the C-terminal one retains the DxR signature at the beginning of strand 4, suggesting a possible separation of the initial adenylating and subsequent polymerization reactions between the two domains. In both bacteria and archaea we found Thg1 to be encoded in distinct gene-neighborhoods that are conserved across phylogenetically distinct lineages (Fig. 2). In bacteria the most persistent association is with a P-loop kinase of the polynucleotide kinase family [14]. Additionally, a subset of these predicted operons include a gene encoding a MACRO domain protein [15,16]. Furthermore, some bacterial gene neighborhoods also show an association between Thg1 and genes encoding a RNA ligase (Fig. 2, Additional File 1). The strong association with the polynucleotide kinase is functionally relevant because this enzyme is known to phosphorylate the 5' OH of RNA - thus, it can generate the phosphorylated 5' terminus, which is the substrate for all experimentally characterized versions of the Thg1 family. We also observed that, in a contextual network prepared from domain architectures operonic associations and physical interactions, Thg1 occupies a position comparable to two other structurally unrelated but catalytically similar nucleotidyltransferase domains (Fig. 2), namely the CCA-adding enzyme/poly A polymerase and the RNA ligase.

Like Thg1, the RNA ligase uses a 5' phosphate produced as a result of RNA repair by the polynucleotide kinase as a substrate to mediate ligation of two polynucleotide chains (e.g. in tRNA splicing) [17]. MACRO domain proteins also function in RNA-splicing reactions and have been implicated in binding ADP-ribose and in hydrolyzing the 1'' phosphate of ADP-ribose-1''-phosphate (Appr1p) generated during splicing [15,16]. Hence, it is possible that the MACRO domain enzymes encoded by a subset of these predicted operons might hydrolyze or bind the App formed at the 5' end of the RNA and thereby regulate the activity of Thg1. In archaea the only persistent genomic association that we recovered was with a gene encoding a protein with a PRC barrel domain. We had previously shown that the PRC barrel is a potential RNA-binding domain [18]; in this case it could function as a substrate-binding partner of the Thg1 protein. In conclusion these associations strongly support a RNA-related function for Thg1 in all the three superkingdoms of life. Further, the specific association with enzymes such as polynucleotide kinase, the MACRO domain and RNA ligase suggests that Thg1 might have a previously unappreciated role in tRNA repair and splicing. Hence, it is possible that its 3'-5' polymerase activity has a significant role in repair of small RNAs, including tRNAs by probably by catalyzing 3'-5' RNA polymerization or by catalyzing RNA ligation via generation of a 5' App.

## Evolutionary considerations and general conclusions

While representatives of the Thg1 family are present in all the three superkingdoms of life, they are far more prevalent in eukaryotes and archaea than in bacteria. Phylogenetic analysis revealed that the Thg1 proteins fall into two major clusters separated by an internal long branch (Fig. 2, Additional File 1). The first of these clusters is almost entirely comprised of eukaryotic proteins with the deepest split being that of *Trichomonas vaginalis*, which is believed to be an early-branching eukaryote. Further, within the eukaryotes, monophyletic lineages such as animals, plants, apicomplexans and ciliates can be discerned suggesting that Thg1 was present in the common ancestor of all extant eukaryotes (Fig. 2). The second cluster is dominated by archaeal Thg1 orthologs, with representatives from euryarchaea, crenarchaea and korarchaea, suggesting that Thg1 was present in the ancestral archaeon. Nested within this group are several bacterial versions distributed along with different archaeal groups (Fig.2, Additional File 1). A few bacterial versions also appear to have been independently transferred to eukaryotes such as slime molds and *Naegleria *(Additional File 1). In particular slime molds possess multiple copies of Thg1, of both the eukaryotic and bacterial type that are all predicted to be catalytically active enzymes. This is unusual as Thg1 is typically present in a single copy per genome, reflective of its specific role in HtRNA maturation. Likewise in seed plants there appear to be multiple copies of the eukaryote-type enzyme with two tandem Thg1 modules. In these cases it possible that the Thg1 paralogs have acquired additional RNA repair functions. Two large DNA viruses, the mimivirus and the caudovirus 201varphi2-1 which infects *Pseudomonas chlororaphis*, also possess their own copies of the Thg1 protein - it is unclear if these viruses needed a dedicated HtRNA maturation enzyme or else use it in an alternative RNA repair mechanism aimed against host defenses. Together, the phyletic distribution and phylogenetic picture suggests that Thg1 originated in the common ancestor of the archaeo-eukaryotic lineage, with more than one lateral transfer from archaea to different bacterial lineages.

The above finding that Thg1 is specifically related to the GGDEF and CRISPR polymerases raises interesting evolutionary and functional issues in light of its inferred point of origin. The GGDEF domains are found in all major bacterial lineages and are inferred to have been present in the common ancestor of the bacterial lineage [19], functioning specifically as a cyclic-diguanylate-generating signaling enzyme. In contrast to most other signaling domains of bacterial provenance, the GGDEF domain is surprisingly entirely absent in eukaryotes [19]. Similarly, conventional GGDEF domains involved in signaling are also absent in archaea, though we observed a small family of archaeal relatives of the GGDEF domain typified by MK0296 from *Methanopyrus kandleri *(Fig. 1). These are predicted to be active enzymes as they retain the three acidic catalytic residues in the RRM-fold palm, but are unlikely to be signaling proteins as they lack associations with any of the typical signaling domains that are always seen in conventional bacterial diguanylate cyclase proteins. We hence postulated that the cyclic diguanylate could be potentially toxic in the archaeo-eukaryotic lineage, perhaps due to interference with a key polymerase active site [19]. The CRISPR polymerase domains are highly mobile along with rest of the CRISPR system, though their predominance in archaea might suggest an initial origin in that lineage [12,20,21]. Thus, most parsimoniously the last universal common ancestor (LUCA) already possessed a distinct version of the RRM-fold palm domain that was the common ancestor of the Thg1, GGDEF and CRISPR polymerase catalytic domains. This ancestral version of the palm domain was already distinct from other RNA and DNA polymerase catalytic domains of the RRM-like fold (Fig. 1). From this ancestor, in the bacterial lineage the GGDEF domain arose, whereas the Thg1 progenitor arose in the archaeo-eukaryotic lineage. Likewise it appears probable that the CRISPR polymerase domain emerged early in the archaeal lineage followed by extensive lateral transfer due to it adaptive role in defense against selfish elements [20,21]. Interestingly, some versions of the GGDEF are known to bind RNA, rather than catalyze cyclic diguanylate formation [22]. Furthermore, recognition of cyclic diguanylate occurs predominantly via RNA molecules i.e. riboswitches, rather than through protein domains (unlike what is observed with cyclic nucleotides like cAMP and cGMP) [19,22].

These findings suggest that the common ancestor of these three families of enzymes might have primarily acted on RNA substrates and that the Thg1 polymerase might represent an activity close to the ancestral state. Given that certain reconstructions of LUCA posit a notable role for RNA-based genetic material [23], it is possible that this ancestral enzyme might have had a prominent role in LUCA as an enzyme that repaired RNA through its polymerase or nucleotidyltransferase activity. However, with the emergence of DNA-based genetic material, its descendant Thg1, in the archaeo-eukaryotic lineage, might have been relegated to a rather subsidiary role in tRNA maturation. Its descendant in the bacterial lineage was recycled as a diguanylate cyclase in a signaling context, whereas the polymerase of the CRISPR system emerged early in archaeal evolution retaining a function closer to its ancestor. Indeed, incorporation of the HtRNA G-1 position into the genome might have allowed Thg1 to be lost in several lineages. These considerations raise the interesting possibility that some GGDEF domains might have RNA-specific nucleotidyltransferase activity and that the CRISPR polymerase, like Thg1, catalyze 3'-5' polymerization. Further investigation of the biochemical predictions presented here might provide novel insights into the function and evolution of nucleic acid polymerases.

## Abbreviations

The organism abbreviations are APMV: Acanthamoeba polyphaga mimivirus; Achl: Arthrobacter chlorophenolicus; Amac: Alteromonas macleodii; Amar: Acaryochloris marina; Aper: Aeropyrum pernix; Apla: Arthrospira platensis; Apro: Archaeoglobus profundus; Atha: Arabidopsis thaliana; BP201phi2-1: Pseudomonas phage 201phi2-1; BPT7: Enterobacteria phage T7; BPphi6: Pseudomonas phage phi6; Bsub: Bacillus subtilis; Bthu: Bacillus thuringiensis; CKor: Candidatus Korarchaeum; Cbac: Clostridiales bacterium; Ceut: Coprococcus eutactus; Cgla: Candida glabrata; Chom: Cardiobacterium hominis; Cint: Ciona intestinalis; Cmat: Corynebacterium matruchotii; Cpar: Cryptosporidium parvum; Cpin: Chitinophaga pinensis; Crei: Chlamydomonas reinhardtii; Ddis: Dictyostelium discoideum; Dmel: Drosophila melanogaster; Ecol: Escherichia coli; Erec: Eubacterium rectale; Gobs: Gemmata obscuriglobus; Gste: Geobacillus stearothermophilus; Gsul: Geobacter sulfurreducens; HCV: Hepatitis C virus (isolate BK); HEVC: Human enterovirus C; Haur: Herpetosiphon aurantiacus; HIV1: Human immunodeficiency virus 1; Hsap: Homo sapiens; Lbic: Laccaria bicolor; Mace: Methanosarcina acetivorans; Mbar: Methanosarcina barkeri; Mbre: Monosiga brevicollis; Mbur: Methanococcoides burtonii; Mcap: Methylococcus capsulatus; Mcht: Microcoleus chthonoplastes; Mgam: marine gamma; Mhun: Methanospirillum hungatei; Mjan: Methanocaldococcus jannaschii; Mkan: Methanopyrus kandleri; Mmar: Microscilla marina; MMLV: Moloney murine leukemia virus; Msmi: Methanobrevibacter smithii; Msta: Methanosphaera stadtmanae; Mthe: Methanothermobacter thermautotrophicus; Mxan: Myxococcus xanthus; Ncra: Neurospora crassa; Nelo: Neisseria elongata; Nfar: Nocardia farcinica; Nvec: Nematostella vectensis; Pfal: Plasmodium falciparum; Plim: Planctomyces limnophilus; Ppac: Plesiocystis pacifica; Ppat: Physcomitrella patens; Ppla: Postia placenta; Psyr: Pseudomonas syringae; Ptet: Paramecium tetraurelia; Pyae: Pyrobaculum aerophilum; S2RNV: Saccharomyces 23S RNA narnavirus; Scel: Sorangium cellulosum; Scer: Saccharomyces cerevisiae; Spom: Schizosaccharomyces pombe; Ssol: Sulfolobus solfataricus; Sulf.: Sulfitobacter sp.; Ster: Sebaldella termitidis; Taqu: Thermus aquaticus; Tbru: Trypanosoma brucei; Tneu: Thermoproteus neutrophilus; Tthe: Tetrahymena thermophila; Tvag: Trichomonas vaginalis; Tvol: Thermoplasma volcanium; Vspi: Verrucomicrobium spinosu.

## Competing interests

The authors declare that they have no competing interests.

## Authors' contributions

VA, LMI and LA performed the reported research and wrote the paper. All authors read and approved the final manuscript.

## Reviewer's Comments

### Reviewer 1

Balaji Santhanam, Center for Cancer Systems Biology, Dana-Farber Cancer Institute

Department of Genetics, Harvard Medical School, Boston MA

*In this manuscript titled "Presence of a classical RRM-fold palm domain in Thg1-type 3'- 5'nucleic acid polymerases and the origin of the GGDEF and CRISPR polymerase domains" Anantharaman et al report the computational discovery of a RRM-fold domain in Thg1 family proteins and its evolutionary relationship to bacterial GGDEF domains and CRISPR polymerases, which are predominantly found in archaea. The authors also provide detailed descriptions of their catalytic sites and functional contexts based on amino-acid residue conservations, domain architectures and protein interaction information. Further, the authors discuss the common evolutionary origins of Thg1, GGDEF and CRISPR polymerase families and propose a biochemical role for the RRM-fold palm domain in LUCA. The manuscript reports fascinating findings and would aid future experimental investigations along these lines. I strongly support the publication of the manuscript in Biology Direct*.

Few points to the authors:

1. Do all Thg1 family proteins have secondary catalytic site or is there any exception? If there is one, in that specific case, can the authors comment on how the adenylation process would be coupled to 3'-5' polymerization?

#### Response

As indicated above the Thg1 orthologs from seed plants have a duplication of the Thg1 polymerase domain. The N-terminal domain lacks the DxR motif associated with secondary active site. We propose that in this case there could be a "division of labor" with the N-terminal domain mediating chain elongation but the C-terminal domain capable of adenylation as well as polymerization.

2. Do the authors find the N-terminal helical extension region to the core RRM domain present in most of Thg1 family proteins? If it is true, do the authors think there is any potential role for this region in directing the specificity for 3'-5' polymerization?

#### Response

The N-terminal helix is present in all the Thg1 proteins. It is certainly functionally important because mutation of a glutamate in this helix reduces activity. However, there are no absolutely conserved residues suggesting that it is unlikely to contribute directly to the active site. It could instead play a role in dimerization.

3.Does the C-terminal HTH-like region of Thg1 have roles other than contributing to active-site biochemistry, like mediating interactions with nucleic acids or proteins?

#### Response

It could interact with the nucleic acid template comparable to the "fingers" module of other nucleic acid polymerases. However, beyond this there is no available evidence that it mediates other interactions.

4.Thg1p of budding yeast seems to form a homodimer based on protein interaction data from two independent experiments. Would this information be helpful in providing additional insights into the functional contexts of Thg1 family? 

#### Response

The seed plant versions contain two distinct Thg1 domains that might show functional differentiation (see above). This observation, together with evidence from yeast for homodimerization, suggests that the Thg1 protein is indeed likely to function as a dimer. It remains to be seen if this dimerization might imply partitioning of adenylation and polymerization between the two monomers in the functional unit.

*5.Thg1p of budding yeast seems to be associated with Orc2p directly in a complex. Again based on the protein- and genetic- interaction data Thg1 (in budding yeast) seems to be associated with *proteins/genes *involved in DNA replication/repair. Is it possible that the Thg1 family proteins could function in the context of DNA replication/repair as well?*

#### Response

This is indeed an intriguing observation - given that Thg1 shows both physical and genetic interactions with Orc2 it is likely that this association is functionally relevant. Of the other genetic interactions of Thg1 there are multiple interactions with genes involved in tRNA- or translation- related functions such as Hts1: the histidinyl tRNA synthetase which utilizes the HtRNA produced by Thg1 as a substrate, Trm11: the tRNA methylase which methylates G10 of tRNAs, and Tma64: a protein with a RNA-binding Sui1 domain involved in RNA processing. These are consistent with the role of Thg1 in HtRNA maturation. However, in addition to Orc2, Thg1 shows genetic interactions with other DNA replication related genes such as Pol32: a winged HTH containing subunit of the DNA polymerase delta required for replication and error prone repair, and Mrc1: A regulator of replication and repair. This suggests that role of Thg1 in DNA replication might indeed be relevant. While there is currently no biochemical evidence that throws light on this role, it would be of interest to investigate if its adenylation or polymerization activity might have a direct role in processes such as priming and ligation.

*6.The authors could indicate catalytic residues explicitly (although mutation data has been mapped) in the figure *1A. *This would greatly aid in comprehending the relevant parts of the manuscript*.

#### Response

The proposed catalytic residues have been indicated by means in blue boxes in Fig. 1.

### Reviewer 2

Valerian V. Dolja, Department of Botany and Plant Pathology, Oregon State University

Cordley Hall 2082, Corvallis, OR

*This incisive study from Aravind's shop uses cutting-edge bioinformatics analyses to add yet another colorful piece to a jigsaw puzzle of the early life origins and evolution. It starts with a relatively obscure albeit mechanistically enigmatic class of Thg1 enzymes involved in maturation of a single tRNA, tRNAHis. Unexpectedly, it uncovers relationships of Thg1 with GGDEF-like catalytic domains, CRISPR system polymerases that are very much in vogue, and, finally, connects them to a vast class of RRM-fold palm domain polymerases*.

*Being a virologist turned cell biologist, I am not in position to comment on the intricacies of the methodology used to unveil structural, mechanistic and phylogenetic relationships of Thg1; I simply trust the authors by virtue of their impressive record. Rather, I would like to emphasize the most intriguing implications of their work. First, their study suggests likely catalytic mechanisms for both Thg1 and CRISPR polymerases that are experimentally testable. Second, it divines the evolutionary scenario for Thg1, GGDEF domains, and CRISPR polymerases that, although not immediately testable, proposes a feasible and broad RNA repair role for their common ancestor. This possibility, in turn, strengthens the argument for primordial RNA-based genetic systems allowing for more faithful RNA replication and larger RNA-based genomes. In addition, it stimulates further inquiry into extant RNA repair systems, a relatively young research area that recently uncovered RNA repair roles for the cellular and viral AlkB demethylases *[1,2].

### References

*Aas, P.A., Otterlei, M., Falnes, P.O., Vagbo, C.B., Skorpen, F., Akbari, M., Sundheim, O., Bjoras, M., Slupphaug, G., Seeberg, E., and Krokan, H.E. (2003). Human and bacterial oxidative demethylases repair alkylation damage in both RNA and DNA. Nature 421, 859-863*.

*van den Born, E., Omelchenko, M.V., Bekkelund, A., Leihne, V., Koonin, E.V., Dolja, V.V., and Falnes, P.O. (2008). Viral AlkB proteins repair RNA damage by oxidative demethylation. Nucleic Acids Res. 36, 5451-5461*.

*I have only two more specific and indeed minor comments*.

*1. It would be helpful to include at least a schematic dendrogram depicting results of phylogenetic analysis (now shown in Addit. File 1) as a panel in a figure*.

#### Response

We include a schematic of the phylogenetic tree shown in Fig.2. However, in the interest of space we only show the major branches in this figure.

2. On page 8, both mimivirus and caudovirus are collectively called 'phages'. Traditionally, this term reserved for viruses of bacteria only, whereas mimiviruses infect unicellular eukaryotes.

#### Response

We alter this to refer to only the caudovirus as a phage.

## Supplementary Material

Additional file 1 **RRM-fold palm domain in Thg1-type 3'- 5'nucleic acid polymerases**. Complete phyletic pattern, domain architectures and alignment data along with more detailed material and methods are provided in this file. The file can also be accessed from: ftp://ftp.ncbi.nih.gov/pub/aravind/Thg1/Thg1.html. Click here for file
