# Supplementary material for: Presence of a classical RRM-fold palm domain in Thg1-type 3'- 5'nucleic acid polymerases and the origin of the GGDEF and CRISPR polymerase domains
Source: Biol Direct. 2010 Jun 30;5:43. doi: 10.1186/1745-6150-5-43 (PMC2904730; doi:10.1186/1745-6150-5-43)
Supplement: Additional file 1 — RRM-fold palm domain in Thg1-type 3'- 5'nucleic acid polymerases. Complete phyletic pattern, domain architectures and alignment data along with more detailed material and methods are provided in this file. The file can also be accessed from: ftp://ftp.ncbi.nih.gov/pub/aravind/Thg1/Thg1.html. [file 1745-6150-5-43-S1.HTML]

RRM-fold palm domain in
Thg1-type 3’- 5’nucleic acid polymerases


# 

**Presence
of a classical RRM-fold palm domain in
Thg1-type 3’- 5’nucleic acid polymerases and the
origin of
the GGDEF and CRISPR
polymerase domains**.

**Vivek
Anantharaman, Lakshminarayan M. Iyer,**  L.
Aravind\*  
  
\* *Address
for
correspondence: L. Aravind (aravind@mail.nih.gov)*  
  
*National
Center for Biotechnology
Information, National Library of Medicine, National Institutes of
Health, Bethesda, MD 20894, USA*


---

  

Abstract  

Almost
all known nucleic acid polymerases catalyze 5’-3’
polymerization by mediating
the attack on an incoming nucleotide 5’ triphosphate by the
3’OH from the
growing polynucleotide chain in a template dependent or independent
manner. The
only known exception to this rule is the Thg1 RNA polymerase that
catalyzes
3’-5’ polymerization *in vitro*
and
also *in vivo*
as a part of the
maturation process of histidinyl tRNA. While the initiating reaction
catalyzed
by Thg1 has been compared to adenylation catalyzed by the aminoacyl
tRNA
synthetases, the evolutionary relationships of Thg1 and the actual
nature of
the polymerase reaction catalyzed by it remain unclear. Using sensitive
profile-profile comparison and structure prediction methods we show
that the
catalytic domain Thg1 contains a RRM (ferredoxin) fold palm domain,
just like
the viral RNA-dependent RNA polymerases, reverse transcriptases, family
A and B
DNA polymerases, adenylyl cyclases, diguanylate cyclases (GGDEF domain)
and the
predicted polymerase of the CRISPR system. We show just as in these
polymerases, Thg1 possesses an active site with three acidic residues
that
chelate Mg++
cations. Based on this we predict that Thg1
catalyzes
polymerization similarly to the 5’-3’ polymerases,
but uses
the incoming 3’ OH
to attack the 5’ triphosphate generated at the end of the
elongating
polynucleotide. In addition we identify a distinct set of residues
unique to
Thg1 that we predict as comprising a second active site, which
catalyzes the
initial adenylation reaction to prime 3’-5’
polymerization.
Based on contextual
information from conserved gene neighborhoods we show that Thg1 might
function in
conjunction with a polynucleotide kinase that generates an initial
5’ phosphate
substrate for it at the end of a RNA molecule. In addition to
histidinyl tRNA
maturation, Thg1 might have other RNA repair roles in representatives
from all
the three superkingdoms of life as well as certain large DNA viruses.
We also
present evidence that among the polymerase-like domains Thg1 is most
closely
related to the catalytic domains of the GGDEF and CRISPR polymerase
proteins.
Based on this relationship and the phyletic patterns of these enzymes
we infer
that the Thg1 protein is likely to represent an archaeo-eukaryotic
branch of
the same family of proteins that gave rise to the mobile CRISPR
polymerases and
in bacteria spawned the GGDEF domains. Thg1 is likely to be close to
the
ancestral version of this family of enzymes that might have played a
role in
RNA repair in the last universal common ancestor.

  


---

Contents  
  

- Materials And Methods
- Multiple
  Alignment of Thg1 domain with the C terminal extension
- Family
  clusters of Thg1 domain containing proteins with domain
  architecture
- Operons
  of Thg1 containing prokaryotic proteins
- Phylogenetic
  tree of
  Thg1 domains

  


---

Materials
And Methods  
  

The
non-redundant (NR) database of protein sequences (National
Center
for Biotechnology
Information,
NIH, Bethesda)
was searched using the BLASTP program [1].
Iterative
database searches were conducted using the PSI-BLAST program with
either a
single sequence or an alignment used as the query, with the PSSM
inclusion
expectation (E) value threshold of 0.01 (unless specified otherwise);
the
searches were iterated until convergence [1,
2].
For all searches with
compositionally biased proteins, the statistical correction for this
bias was
employed. Multiple alignments were constructed using the KALIGN
programs [3,
4],
followed by manual correction based
on the PSI-BLAST results. Globular domains were predicted using the SEG
program
with the following parameters: window size 40, trigger complexity=3.4;
extension complexity=3.75 [5].
All large-scale
sequence analysis procedures were carried out using the TASS package
developed
by our group (unpublished; Vivek Anantharaman, Santhanam Balaji, and L.
Aravind). Protein secondary structure was predicted using a
multiple
alignment as the input for the JPRED program [6].
Similarity-based
clustering of proteins was carried out using the BLASTCLUST program
(For
documentation see ftp://ftp.ncbi.nih.gov/blast/documents/README.bcl).
Profile-based HMM searches [7]
and JACKHMMER 
were performed using the newly released HMMER3 package (version beta 2)
(http://hmmer.janelia.org/). 
Pairwise comparisons of HMMs, against profiles were performed with the
HHPRED program [8] (http://toolkit.tuebingen.mpg.de/hhpred).
 

 References

1.           
Altschul
SF, Madden TL, Schaffer AA, Zhang J, Zhang Z, Miller W, Lipman DJ:
Gapped BLAST and PSI-BLAST: a new
generation of protein database search programs. *Nucleic
Acids Res* 1997,
25(17):3389-3402.

2.           
Aravind L, Koonin EV:
Gleaning non-trivial structural,
functional
and evolutionary information about proteins by iterative database
searches.
*J Mol Biol* 1999,
287(5):1023-1040.

3.           
Lassmann T, Sonnhammer EL:
Kalign--an accurate and fast multiple
sequence alignment algorithm. *BMC
Bioinformatics* 2005, 6:298.

4.           
Lassmann T, Sonnhammer EL:
Kalign, Kalignvu and Mumsa: web
servers for
multiple sequence alignment. *Nucleic
Acids Res* 2006, 34(Web Server
issue):W596-599.

5.           
Wootton JC: Non-globular
domains in protein sequences: automated
segmentation using
complexity measures. *Comput
Chem* 1994,
18(3):269-285.

6.           
Cuff JA, Barton GJ:
Application of multiple sequence alignment
profiles to improve protein secondary structure prediction. *Proteins*2000, 40(3):502-511.

7.
           Eddy
SR: Profile hidden Markov
models. Bioinformatics
1998,
14:755-763.

8.
           Soding
J: Protein homology
detection by HMM-HMM comparison. Bioinformatics
2005, 21:951-960.

 

   
  
  
  
  
Top  


---

  
  
Multiple
Alignment of Thg1 domain with the C terminal extension   

|  |
| --- |
| ---------------------------E1-------------------------------H1------------------E2-------E3-------------------------------------H2----------------------------------------------------------------E4--------->>>C terminal Extension  FINAL                                                 ------HHHHHHH------------EEEEEE-----HH-HHHH----------HHHHHHHHHHHHHHHHHH-------EEEEE---EEEEEEE--------------------------HHHHHHHHHHHHHHHHHHHH------------------------------------------------------EEEE-----HHHHHHHHHHHH-HHH--HHHHHHHHHHHHHHH-----HHHHHHHHH---HHHHHHHHHHHH---------------EEEEE-------------------------------------------------------------------EEEE-----------------EEEEE------EEEEEE---------------H--HHHHHH------  THG1\_Scer\_6321461                                     MANSKFGYVRQFETHDV--ILP-QCYIVVRIDGKKFHE-FSKFYEFAKP---NDENALKLMNACAKNLVLK--YKNDIILAFGESDEYSFILKSS----------------TTLFNRRKDKLATLFGSFFTSNYVALWAKFFPEKPLNIKH------------------------------------LPYFDSRCVAYPN--LQTIKDYLSWRY-VDT--HINNLYNTTFWQLIIKCGLTPQESEKKLCGTFSNEKQEILFSECGINYNNEP-EMFKKGSLVTR---------------------------------------------------------------------------------------------------KGEILHI-----------NVIAQIDELFEG-------    6321461         Saccharomyces cerevisiae                                eukaryota>fungi>ascomycota  ZYRO0A12144g\_Zrou\_254577809                           MAKSRFEYVRDFEQRDV--LIP-ETYIVVRIDGKKFHE-FSNYYKFEKP---NDMRALKLMNACAKNVLLQ--YRSEVILAYGESDEYSFILRSD----------------TTLYNRRIDKISSLFVSLFTSQYVMLWSKFFPNDPLDVKH------------------------------------LPFFDSRCVSYPN--LKCIKDYLSWRF-VDT--HINNLYNTVFWQLVQKCGLTTREAENKLSGTVSSEKQEILFSECGINYNNEP-EMFKKGSLVTR---------------------------------------------------------------------------------------------------KGEILHI-----------DVIKKIDELFEG-------    254577809       Zygosaccharomyces rouxii CBS 732                        eukaryota>fungi>ascomycota  KLTH0G17270g\_Lthe\_255718999                           MAKSRFEYVRQFESHET--LLP-ETYIVVRIDGKKFHE-FSKHYNFEKP---NDERALKLMNASAKNVVLK--YKNDVVLAFGESDEYSFILKKD----------------TSLFKRRRDKLSSLFVSLFTANYVALWSKFFPGTALDYKH------------------------------------LPFFDSRCVCYPN--LSTIKDYLCWRY-VDT--HINNLYNTVFWQLIIKCGLTPQESEQKLCGTLSSDKQEILFTDCGINYNDEP-NMFKKGSLVTN---------------------------------------------------------------------------------------------------KGEVCHI-----------DVVKQIDVLFEG-------    255718999       Lachancea thermotolerans CBS 6340                       eukaryota>fungi>ascomycota  AGOS\_ABR031C\_Agos\_45185260                            MAKSRFEYVREYEVHDT--LLP-ETYIVVRIDGKHFHE-FSQHYSFEKP---NDERALKLMNASAKNVVMA--YSGDIILAFGESDEYSFILRKD----------------STLFRRRRDKLSTLFVSLFTAQYVALWPKFFPEQPLSHKR------------------------------------LPFFDSRCVCYPN--TTVVKDYLCWRY-VDT--HINNLYNTVFWNLVLKCNLTPREAEQRLSGTLSSDKQEILFSECGVNYNNES-EMFKKGSLINR---------------------------------------------------------------------------------------------------KGEIMHI-----------DVVKQIDELFAG-------    45185260        Ashbya gossypii ATCC 10895                              eukaryota>fungi>ascomycota  EC1118\_1G1\_3257g\_Scer\_259146527                       MANSKFGYVRQFETHDV--ILP-QCYIVARIDGKKFHE-FSKFYEFAKP---NDENALKLMNECAKNLVLK--YKNDIILAFGESDEYSFILKSS----------------TTLFNRRKDKLATLFGSFFTSNYVALWAKFFPEKPLNIKH------------------------------------LPYFDSRCVAYPN--LQTIKDYLSWRY-VDT--HINNLYNTTFWQLIIKCGLTPQESEKKLCGTFSNEKQEILFSECGINYNNEP-EMFKKGSLVTR---------------------------------------------------------------------------------------------------NGEILHI-----------NVIAQIDELFEG-------    259146527       Saccharomyces cerevisiae EC1118                         eukaryota>fungi>ascomycota  KLLA0B06237g\_Klac\_50303737                            MAKSRFEYVRQFEVHDA--LLP-DTYIVVRVDGKKFHE-FSKYYNFAKP---NDERALKLMNAAAKNVFMQ--YKQEMICAYGESDEYSFILKRD----------------TKLFNRRRDKISTLFVSLFTANYVSLWNLFFPDVVLHHKH------------------------------------LPYFDSRCVCYPN--LTVVKDYLSWRF-VDT--HINNLYNTVFWYLIIKCGLTPQESEQKLCGTLSSDKQEILFSECGINYNNEP-EMYKKGSLVNS---------------------------------------------------------------------------------------------------KGEIVHI-----------DVVKQIDEIFNG-------    50303737        Kluyveromyces lactis NRRL Y-1140                        eukaryota>fungi>ascomycota  Kpol\_526p19\_Vpol\_156843110                            MANSRFEYVKQFETHDT--LLP-QTYIVVRIDGKKFHE-FSKFYDFAKP---NDERALKLMNACAKNVVLH--YKTDMIVAFGESDEYSFILKKD----------------TTLYRRRSEKLSTLFVSLFTSNYVALWPKFFPDTPLNHKY------------------------------------LPFFDSRCVVYPN--IQIIKDYLSWRF-VDT--HINNLYNTAFWQLIQICGLTAQEAEKKLMGTFSNDKQEILFTECGINYNNEP-EMYKKGSLITR---------------------------------------------------------------------------------------------------KGEILHI-----------DVIKQINELFVG-------    156843110       Vanderwaltozyma polyspora DSM 70294                     eukaryota>fungi>ascomycota  UM00383.1\_Umay\_71003728                               MAGTKFAYVRNFELPDS--VIP-STYLVVRIDGKGFHK-FSAAHTFAKP---NDALALELMNEAARYVMYS--LKGHIALAFGESDEYSFLLRRS----------------TTLYNRRNSKITTHIVSLFTSAYVFSWSKFFPRNPL--LH------------------------------------PPSFDGRMVVYPN--EKVVRDYFSWRQ-ADT--HINNLYNTVFWALVLQAGLTEQEATKRLEGTVSADKHEILHSQFGINYDRLE-AMFRKGTTLVW-------------------------tcadssqeipshstaatgsqmeaekavtsdtveetqvltnlkdeklriknesrrvaakqakqekkdkks----MLSHHRHR-----------SNRD--LSWLTA-------    71003728        Ustilago maydis 521                                     eukaryota>fungi>basidiomycota  CAGL0J00209g\_Cgla\_50290577                            MAKSKYEYVKQFESHDT--LLP-QCYIVVRIDGKKFHE-FSKYYDFKKP---NDERALKLMNACAKNVVLQ--YRHEMILAYGESDEYSFVLKKD----------------TELYKRRRDKLSTLIVSLFTSNYVALWSKFFPGTNLHPKH------------------------------------LPFFDSRCVIYPN--LETIRDYVTWRY-VDT--HINNLYNTAFWQLIQKCGMNPQEAEKRLSGTVSSEKNEILFKECGINYNNEP-EMYKKGSLITN---------------------------------------------------------------------------------------------------KGEILHI-----------NVI---DSLDSLFEG----    50290577        Candida glabrata CBS 138                                eukaryota>fungi>ascomycota  AgaP\_AGAP011752\_Agam\_158300966                        MALSRFEYVKQFEQEEK--LLP-NSWIVVRIDGKGFHR-FCNVHSFSKP---NDLDALQLMNLAGMTVLQE--F-NEIAIGYGQSDEYSFVFRRE----------------ASVYQRRRDKLVSYVASLFTSAYMFHWKRIFDGRSIAMRY------------------------------------PPSFDARAVLYPT--DENLRDYLSWRQ-ADV--HVNNLYNTTFWNLV-ASGLSNSDAEKRLQGTLASDKNEILFSQFGINYNNEP-IIYRKGTILLP-----------------------------------------------------------------------------------ksvvssegkkq----RLIVPIFE-----------DLIS--DAFWAKHPEILD-    158300966       Anopheles gambiae str. PEST                             eukaryota>metazoa>hexapoda  CLUG\_03111\_Clus\_260946739                             MANSRFEYVKAFERENY--LLP-DTYIVIRVDGKGFHK-FSQEYSFAKP---NDVRALQVMNRAASAMLEQ--F-PDITMAYGDSDEYSFLLRRS----------------CALFERREMKLVSTFASFMSVHYVMQWNQEFPEKQIHSGR------------------------------------LPTFDARAVVYPN--AAVLRDYFSWRQ-VDC--HINNLYNTSFWGLVQKCGMSGQEAEQALSGTLAADKNEILFSRCGINYNNEP-EMFKKGSIIVR-------------------------------------------------------------------eyedwtpvdekgmtprqkqrvqkrrsk----AEIKVYHE-----------DIIK--DGFWDARPWLLE-    260946739       Clavispora lusitaniae ATCC 42720                        eukaryota>fungi>ascomycota  Phum\_PHUM410370\_Phum\_242016870                        MAKSKFEYVKEFETEDK--CLP-NTWIVVRVDGRGFSK-FSERYKFKKP---NDVRALSVMNAAAKAVMKE--F-PEIILAYGQSDEYSFVFKKD----------------STLYNRRIQKLTSVISSAFTAFYITLWPKYFGNESITT--------------------------------------PPIFDGRVVCYPS--ENNLRDYLSWRQ-TDT--HINNLYNTCFWNLVTNKKMSHKEAEEHLKGTVSSDKNELLFTEFGINYNNEP-EEFRKGTVIIR-------------------------------------------------------------------------------------------gs-----KKKLETHNC----------DIIG--NNFWDDHKELLN-    242016870       Pediculus humanus corporis                              eukaryota>metazoa>hexapoda  zgc:101609\_Drer\_55925403                              MAKSKFEYVRNFELDDT--CLR-NCYIVVRLDGRNLHK-FSDQHNFTKP---NDDRALGLMSRSACSVMEE--L-DDITIAYGQSDEFSFVFKRS----------------TNWFKRRASKLMTHVTSQFSSSFVFYWKEYFGEQPLLY--------------------------------------PPSFDGRVVLYPS--NRNVRDYLSWRQ-ADC--HINNLYNTAFWTLVQKGGLTTTQAEERLNGTLATDKNEILFSEFNINYNNES-SVYKKGTTLIW-------------------------------------------------------------------ekvnetttkqikrpneeetevtvtrt-----RKKVTSHSC----------DVIG--DQFWDEHPDILE-    55925403        Danio rerio                                             eukaryota>metazoa>vertebrata>actinopterygii  NEMVEDRAFT\_v1g84802\_Nvec\_156403638                    MAKSKYEYVRKFEQNDA--CLP-NCWIVVRVDGRNFHR-FSDSHGFKKP---NDPRGLGLMNKCAEAVMTE--F-GDIVICYGQSDEYSFVFRKN----------------TTQFSRRVSKLITNVVSLFAATYVFHWASFFTDQHLLY--------------------------------------PPMFDGRVVLYPS--DKNLRDYLSWRQ-ADC--HINNLYNTCFWCLVNQGGVTQTKAEERLCGTVSSDKNELLFSEFDVNYNNEP-ELYRKGSILIW--------------------------------------------------------------------------vqkneasteenttdkkkrir----RKVTTLHT-----------DIIG--DGFWDEHREILT-    156403638       Nematostella vectensis                                  eukaryota>metazoa>cnidaria  PF07\_0095\_Pfal\_124511996                              MANSKFSYVKLFEEERK--ILL-NCYFIVRIDGCDFKH-FVKAHNYNKP---NDIKGLNLMNECALDILKK--F-DDIDLCYGHSDEYSFLFNKS----------------TKLWNRRYDKILTNVVSYFTSCFLYKWKNYFQKEMLY---------------------------------------APSFDARIVVYPN--EKEIKDYFSWRQ-VDC--HINTQYNECFWNLIRQANYTNDEAHKFLLTTQTKDKNELLFSRFNINYNNLP-EIFRRGTIIIR-------------------------------------------nktfqkknealgtiynndtanniddnnnnnnnnnntfndnndienfeklkkecl-PKFIISHE-----------NLVS--EKFWDKYDYIFK-    124511996       Plasmodium falciparum 3D7                               eukaryota>alveolata>apicomplexa  TTHERM\_00085230\_Tthe\_118358858                        MACSQYEYVKKFETYQT--LLP-NTYIVVRIDGKGFTK-FTANHNFEKP---NDKRGLDLMNKAAESVMET--F-NEIMLAYGQSDEFSFVFKKD----------------AELYQRRTEKIVSCVVSCFTAAYAMHFSDYFNIKPSF---------------------------------------LPMFDARAVCYPD--FKNLRDYLNWRQ-VDC--HINNLYNTCFWTMVQKGNMTPQKAQEILKDTLSDRKNEILFNDYGINYAKLE-PQFRKGSTLIRvlvlkdkhankeaeqaesqqkrekceenqglnkegalqqqneegeeqkenaeellkeesdsvmtkgekkyiagqkkqkklnskq-ekiekskyr----SKIITLSD-----------DIIQ--NEFWDKYKDSLK-    118358858       Tetrahymena thermophila                                 eukaryota>alveolata>ciliophora  POSPLDRAFT\_88866\_Ppla\_242214759                       MAGSRYQYVKKFELPDP--ILP-GTFMVLRIDGHAFHR-LSEVHKFAKP---NDERALQLMDHAARDVMNE--Y-KDIVLAFGESDEYSFLFRKS----------------TALYNRRQAKIVTTLTSLFTSSYVFNWSRYLPDTPLEY--------------------------------------PPSFDGRIVVYPS--QKEIRDYFSWRQ-ADT--HINNLYNTIFWALVQQGGETTTQAHAILRGTVSGTKNEMLHSRFGINYNTIP-ARYRKGSVLVQervcslcswifelppassddlastagetp-----------gtpqqpeqapsg-----------------------------sskqkaskkahal----TRIELHHC-----------DIIG--DEFWDQRPYLLA-    242214759       Postia placenta Mad-698-R                               eukaryota>fungi>basidiomycota  POSPLDRAFT\_87675\_Ppla\_242205948                       MAGSRYQYVKTFELPDP--ILP-GTFMVLRIDGHAFHR-LSEVHKFAKP---NDERALQLMDHAARDVMNE--Y-KDIVLAFGESDEYSFLFRKS----------------TALYNRRQAKIVTTLTSLFTSSYVFNWSRYLPDTPLEY--------------------------------------PPSFDGRIVVYPS--QKEIRDYFSWRQ-ADT--HINNLYNTIFWALVQQGGETTTQAHATLRGTVSGTKNEMLHSRFGINYNTIP-ARYRKGSVLVQersppassddlastagetqg---------------------tpqqpeqapsg-----------------------------ssrqkaskkahal----TKIELHHC-----------DIIG--DEFWDQRPYLLA-    242205948       Postia placenta Mad-698-R                               eukaryota>fungi>basidiomycota  VDBG\_07404\_Valb\_261358866                             MANSKFEYVKAFEQPDT--LLQ-NTWVVVRIDGRGFTK-LCAKYAFEKP---NDKRALDLMNAAARAVMTD--L-PDITIAYGISDEYSFVFHKS----------------CTLFERRASKLVTTIVSTFTAHYIHSWSTYFPDAPLTLP-------------------------------------LPSFDGRAVCYPS--VQNLRDYMSWRQ-VDC--HINNLYNTTFWALIQLGGMDNRAAEELLAGTVSGDKNEILFSRFHINYNNEP-EMYKKGSVVFRdyelve----------------------------------pgthnvqadadaia---------------------epvsmtksqtekdkkrrnk----ARIVIEHL-----------DIIK--DDFWDRRPWLLS-    261358866       Verticillium albo-atrum VaMs.102                        eukaryota>fungi>ascomycota  NECHADRAFT\_30739\_Nhae\_256734706                       ---TRFEYVRTFETTDA--LLP-NTWIVVRVDGRGFTK-MCAKYGFEKP---NDRRALDLMNTAAKAVVTD--L-PEITIAYGVSDEYSFVFHKA----------------CTLFERRASKLVSTVVSTFTANYVFSWSTHFPDTPLSYP-------------------------------------LPTFDGRAVCYPS--VQNLRDYMSWRQ-VDC--HINNLYNTTFWSLIQLGGLDNKEAERTLAGTLAADKNEILFSRFSINYNNEP-EIFKKGSVIFR----------------------------------------d-----------------------------------pvqqsksqkekdkksrak----ARVVVEHL-----------DIIK--DDFWDRRPWLLS-    256734706       Nectria haematococca mpVI 77-13-4                       eukaryota>fungi>ascomycota  PODANSg09507\_Pans\_171695070                           ---------------MT--VTN-MSLASLPNLSFCLTR-FSSKGFEKYP---NDRRALDLMNAAAQAVMYE--I-PEVTIAYGISDEYSFVFHKS----------------CTLFERRSSKLVTTIVSTFSSYYIHLWSTYFPDTPLSPP-------------------------------------LPSFDGRAVCYPS--VQNLRDYMSWRQ-VDC--HINNLYNTTFWALIQKGGMGNLEAEELLKGTYAADKNEILFSKFGINYNNEP-EIYKKGSVLFRglvd------------------------------------pathnaaaeadsla---------------------epvqqsknqtesdkkrrak----ARIVIEHL-----------DIIK--DEFWDRRPWLLS-    171695070       Podospora anserina S mat+                               eukaryota>fungi>ascomycota  PGUG\_00408\_Pgui\_190344604                             MANSRYEYVRQFERENF--LLP-DTYIIVRVDGKGFHK-FSDYYKFAKP---NDVGALEVMNEAALHVFKQ--I-SDIVMAYGDSDEYSFLLRKK----------------CSLYERREMKIVTLFAATMAAAYQHIWNTKFPEKPLQLER------------------------------------LPIFDARAVVYPS--MEHVSDYFRWRQ-VDC--HINNLYNTTFWALVSKGGMSPKEAENRLIGTVSSDKNEILFKEFGINYNNEP-EIFKKGTVIVR-------------------------------------------------------------------eyqesv--desqlsqrqkqryekkrgk----ASIMVHHI-----------DLIK--NDFWDSRPWLQN-    190344604       Pichia guilliermondii ATCC 6260                         eukaryota>fungi>ascomycota  PGUG\_00408\_Pgui\_146422179                             MANSRYEYVRQFERENF--LLP-DTYIIVRVDGKGFHK-FSDYYKFAKP---NDVGALEVMNEAALHVFKQ--I-SDIVMAYGDSDEYSFLLRKK----------------CSLYERREMKIVTLFAATMAAAYQHIWNTKFPEKPLQLER------------------------------------LPIFDARAVVYPS--MEHVSDYFRWRQ-VDC--HINNLYNTTFWALVLKGGMSPKEAENRLIGTVSSDKNEILFKEFGINYNNEP-EIFKKGTVIVR-------------------------------------------------------------------eyqesv--desqlsqrqkqryekkrgk----ASIMVHHI-----------DLIK--NDFWDSRPWLQN-    146422179       Pichia guilliermondii ATCC 6260                         eukaryota>fungi>ascomycota  LOC100264494\_Vvin\_225438521                           INNIKPEYVKSFQFENK--LMP-LTWIVVRIDGCHFHR-FSEVHEFEKP---NDEQALNLMNSCAVAVLEQ--F-KDVIFSYGVSDEYSFVLKKD----------------SQFYQRRGSEIVTALVSYFTSIYVMKWKEFFPQKELKY--------------------------------------PPCFDGRAVLYPT--SEILRDYLTWRQ-VDC--HINNQYNTCFWMLV-KSGKSKSEAQAYLKGTQAREKNEVLLQQFGIDYNTLP-LMFRQGSSAFW--------------------------------------------------------------------------neeektgmye-ngasaeksg----KKVVVEQC-----------NIIE--QSFWEAHPSILN-    225438521       Vitis vinifera                                          eukaryota>viridiplantae  GSVIVT01022253001\_Vvin\_270232206                      INNIKPEYVKSFQFENK--LMP-LTWIVVRIDGCHFHR-FSEVHEFEKP---NDEQALNLMNSCAVAVLEQ--F-KDVIFSYGVSDEYSFVLKKD----------------SQFYQRRGSEIVTALVSYFTSIYVMKWKEFFPQKELKY--------------------------------------PPCFDGRAVLYPT--SEILRDYLTWRQ-VDC--HINNQYNTCFWMLV-KSGKSKSEAQAYLKGTQAREKNEVLLQQFGIDYNTLP-LMFRQGSSAFW--------------------------------------------------------------------------neeektgmye-ngasaeksg----KKVVVEQC-----------NIIE--QSFWEAHPSILN-    270232206       Vitis vinifera                                          eukaryota>viridiplantae  AT2G31580\_Atha\_42569521                               IGKIEPDFIRSFQFENK--LLP-LTWVVVRIDGCHFHR-FSDVHEFEKP---NDEQALKLMNSCAVAVLEE--F-EDIHFAYGVSDEYSFVLKKE----------------SELYKRQSSKIISAVASFFTSTYVLQWGEFFPHKELKY--------------------------------------PPSFDGRAVCYPT--YNILLDYLAWRQ-VDC--HINNQYNTCFWMLV-KSGKNKTQSQDYLKGTQTREKNELLSRQFGIEYNSLP-VIFRMGSSVFR-------------------------------------------------------------------------lkeaen------gvvsgkkle----GEVVVDHC-----------NIIE--RCFWEEHLHILS-    42569521        Arabidopsis thaliana                                    eukaryota>viridiplantae  yg1g\_Omyk\_259089291                                   MAKSKFEYVRNFETDDT--CLK-NCYIVVRLDGRNFHK-FAEQHNFLKP---NDDRALGLMTCSARSVMED--P-DDIIISYGQSDEFSFVFKRT----------------SNWFKRRASKLMTHVASQFSSSYVFYWKDYFGDQPLLY--------------------------------------PPGFDGRVVLYPS--NRNLRGYLSWRQ-ADC--HVNNLYNTVFWTLVQKGGLTTTQAEDRLKGTLASDKNEIMFFEFDINYNKEP-LVHRKGTTLIW-------------------------------------------------------------------ekleetvtksvklpneageevlvtrtr----RGVSAHHC-----------DVIG--NQFWEEHPNILE-    259089291       Oncorhynchus mykiss                                     eukaryota>metazoa>vertebrata>actinopterygii  thg1\_Ssal\_213514008                                   MAKSKFEYVRNFETDDT--CLK-NCYIVVRLDGRNFHK-FAEQHNFMKP---NDDRALGLMTCSARSVMED--L-DDIIISYGQSDEFSFVFKRT----------------SNWFKRRASKLMTHVASQFSSSYVFYWRDYFGDQPLLY--------------------------------------PPGFDGRVVLYPT--NRNLRDYLSWRQ-ADC--HVNNLYNTVFWTLVQKGGLTTTQAEDRLKGTLAADKNEIMFSEFDINYNKEP-LVHRKGTTLIW-------------------------------------------------------------------ekleetvtksvklpneageevlvtrtr----RRVGAHHC-----------DVIG--SQFWEEHPNILE-    213514008       Salmo salar                                             eukaryota>metazoa>vertebrata>actinopterygii  THG1\_Eluc\_225716354                                   MAKSKFEYVRNFEADDT--CLK-NCYIVVRLDGRNFHK-LSELHKFLKP---NDDRALGLMTRCARSVMED--M-DDIIISYGQSDEYSFIFKRS----------------SNWFKRRASKLMTHVVSQFSSSYVFYWKEYFGGQPLLY--------------------------------------PPGFDGRVVLYPS--NRNLRDYLSWRQ-ADC--HINNLYNTVFWTLVQRGGLTTTQAEDRLKGTLAADKNEIMFSEFDINYNNEP-LVHRKGTALIW-------------------------------------------------------------------ekmeetvtkrvklpsedekevpvtrfr----RRVSACHC-----------DIIG--DQFWEEHPDILE-    225716354       Esox lucius                                             eukaryota>metazoa>vertebrata>actinopterygii  BRAFLDRAFT\_60263\_Bflo\_260828313                       MAKSKFEYVRQFETQDP--CLP-NTWIVIRLDGRNFHR-FSTDHGFTKP---NDERALQLMNRAAETVMND--F-RDIVISYGQSDEFSFVLKKS----------------TMLYSRRASKLMTHIVSQFSSSYVFHWAQHFPDQPLQY--------------------------------------PPGFDGRVILYPS--NKNLRDYLSWRQ-ADC--HINNLYNTCFWALVQQGGMTNKQAEERIRHTFSADKNEILFSEFGINYNNEP-EMYKKGTVLVW------------------------------------------------------------------krveetvmkacrteddpverprevtklr----KKVVPLHT-----------DIIG--DVFWEEHPDILE-    260828313       Branchiostoma floridae                                  eukaryota>metazoa  LACBIDRAFT\_252840\_Lbic\_170108182                      MANSKYAYVRDFELPDP--LLP-GVFMVLRIDGHSFHR-FSEKHNFAKP---NDVRALQLMDHAAQDLMEE--Y-PDVVLGFGESDEFSFLLRKS----------------TSLYNRRHSKIVSTLTSLFTSSYVFHWSRYFPDTPLGY--------------------------------------PPSFDGRIVLYPG--TKEVKDYFAWRQ-ADT--HINNLYNTTFWALVQQGGQTTTEAHSTLRGTFAKDKHEILFSRFGINYNQLD-AQFRKGSVLVR------------------------------------------------------------------------------edleeekdgevsrrpk----SKVVMLHC-----------DIIK--DEFWEERSGLLV-    170108182       Laccaria bicolor S238N-H82                              eukaryota>fungi>basidiomycota  LOC100165160\_Apis\_193673956                           MAKSKYEYVRTFEFEDR--CLQ-NCWIVVRLDGRSFHR-FTSSHKFEKP---NDKRALELMNRSAAAVMEE--F-RDVSLAYGQSDEYSFILRKN----------------TDLYNRRQSKIMSAINSIFSASYVYYWNTFFEDKKLLY--------------------------------------PPSFDARIVLYPT--DQNLRDYLSWRQ-ADT--HINNLYNTAFWGLISMKGLSNNEAEKVLSGTVSSEKNEILFKECGTNYNNEL-PIYRKGTVLVR----------------------------------------------------------------------------------klvnlppskskk----HVICPLHV-----------DIIG--DSFWEEYNEILN-    193673956       Acyrthosiphon pisum                                     eukaryota>metazoa>hexapoda  THG1L\_Ggal\_71895317                                   MAKSKFEYVRDFETDDT--CLP-NCWIVVRLDGRNFHR-FAEQHEFKKP---NDDRALHLMTKCAQTVMQE--L-EDIAIAYGQSDEYSFVFKKK----------------SKWFKRRASKFMTHVVSQFASSYVFYWKDYFKDQQLLY--------------------------------------PPGFDGRIVLYPS--NQNLKDYLSWRQ-ADC--HINNLYNTVFWMLVQRSGLTPVQAQDRLQGTLAGDKNEILFSEFNINYNNEP-LMYRKGTVLIW------------------------------------------------------------------qkvnevmtkkiklpkeseekevevtrtr----TKVVPLHC-----------DIIG--EQFWEEYPEILA-    71895317        Gallus gallus                                           eukaryota>metazoa>vertebrata  ACYPI006122\_Apis\_239788252                            MAKSKYEYVRTFEFEDR--CLQ-NCWIVVRLDGRSFHR-FTSSHKFEKP---NDKRALELMNRSAAAVMEE--F-RDVSLAYGQSDEYSFILRKN----------------TDLYNRRQSKIMSAINSIFSASYVYYWNTFFEDKKLLY--------------------------------------PPSFDARIVLYPT--DQNLRDYLSWRQ-ADT--HINNLYNTAFWGLISMKGLSNNEAEKVLSGTVSSEKNEILFKECGTNYNNEL-PIYRKGTVLVR------------------------------------------------------------------klvnlpp----------------skskk----HVICPLHV-----------DIIG--DSFWEEYNEILN-    239788252       Acyrthosiphon pisum                                     eukaryota>metazoa>hexapoda  LOC100223262\_Tgut\_224067593                           MAKSKFEYVRDFEADDT--VLP-NCWIVVRLDGRNFHR-FSEQHEFKKP---NDDRALQLMTKCAQTVMQE--L-EDIAIAYGQSDEYSFVFKKK----------------SRWFKRRASKFMTHVVSQFSSSYVFYWKDYFKDQQLLY--------------------------------------PPGFDGRIVLYPS--NQNLKDYLSWRQ-ADC--HINNLYNTVFWMLVQRGGLTPVQAQERLRGTLAGDKNEILFSEFNINYNNEP-LMYRKGTVLIW------------------------------------------------------------------qkinevitkkiklpkeeeekevevtrtk----TKVVPLHC-----------DIIG--DQFWEEYPEILA-    224067593       Taeniopygia guttata                                     eukaryota>metazoa>vertebrata  LOC100179812\_Cint\_198436739                           MTHSQYDYVKSFEIPDP--CLP-NCWPVVRIDGKNFHK-FSTTHNFEKP---NDDRALKLMTAAATSVMDL--L-NDIVLAYGQSDEYSFVFKKK----------------TKAYNRRSSKLSSVVVSQFSTSYVYHWKKYFPDLELQY--------------------------------------PPAFDSRLVLYPS--DKNLRDYLSWRQ-VDC--HINNMYNYCFWKLV-QSGLTHVESQERLKGTLSGDKNELLFSQFGINYNNLP-QLHRKGTVIIR----------------------------------------dk--se-----------------------------------ngdctpgkngkdk----VAVVALNT-----------DIIG--KQFWEGRPHLLA-    198436739       Ciona intestinalis                                      eukaryota>metazoa  LOC663767\_Tcas\_91083329                               MAQSKFEYVRKFETEEK--LLP-NCWIVVRIDGRAFHQ-FSTKHNFKKP---NDESALALMNKAASVVMNE--F-KDIVLAYGQSDEYSFVLRKD----------------TALYNRRGPKIMTYLSSLFTSSYVYHWNQFFKETKLKY--------------------------------------PPAFDARVVLYPS--DQNLRDYLSWRQ-ADC--HINNLYNTTFWALVLKGGLTNNEAEKRLCGTLSSDKNEILFSEFNTNYNNEP-EMFKKGTILIR----------------------------------------------------------------------------------krikspkhgkar----LVILPLHE-----------DLIQ--DKFWEKNSEILA-    91083329        Tribolium castaneum                                     eukaryota>metazoa>hexapoda  Pc16g14050\_Pchr\_255941868                             MANSRFEYVKSFEQPDV--LLP-NTWIVVRIDGRGFHK-LSDHYAFAKP---NDRRALDLMNAAAVEVMKE--L-PDLCIAYGVSDEYSFVFHPN----------------CQLFERRNGKLVTTIVSTFTAHYIYKWSEYFPDRLLLPSH------------------------------------LPSFDGRAVIYPN--NRILRDYMSWRQ-VDC--HINNLYNTTFWTMVLQGGMSNTDAEQELKGTVSADKNEILFKRFGINYNNEL-EIYKKGTVLYR----------------------------------------qvy-eespv--------------------------seskqsrsqqdkirklrrk----AQVVIDHV-----------DIIK--DEFWEKRPWILS-    255941868       Penicillium chrysogenum Wisconsin 54-1255               eukaryota>fungi>ascomycota  PKH\_031670\_Pkno\_221052852                             MANSKFAYVKQLEEERR--VLP-CCYFVVRIDGGNFKA-FTKTHGYTKP---NDIRGLHLMNACAKEVMEK--F-DEIDLAYGHSDEYSFLFRKK----------------TKVWNRRYDKILTNVVSCFSGSFPFLWKIFFPEQELLY--------------------------------------VPSFDGRIVLLPT--EREAKDYFRWRQ-VDC--HINTQYNECFWNLINKDGYSHQQAYNTLITTQKKEKNELLFSKFGINYNDLP-EIFRRGSILMRt---------------------------------------eqilkgghvaeqvditqmesdk-------------vappeeipsdaticqrdal----EKFTLSHE-----------NLVS--DIFWEKYHFLFA-    221052852       Plasmodium knowlesi strain H                            eukaryota>alveolata>apicomplexa  TP01\_0248\_Tpar\_71032267                               MANSKYSYVREFEQDST--LLN-DCWIVVRVDGRAFSA-FSNRHEFRKP---NEPRALSVINAAAVNVMSE--F-DDIVLAYGHSDEYR--------------------------RKQ--KILSCVVSVFTSSYCYYWDTFFPNRPLLT--------------------------------------VPSFDGRVILYPT--YESVLDYFSWRH-VDC--HINNQYNTCFWCLILD-GKSNDEAYNWLKGTTKLEKNEYLFTSHKLNYNNLP-NIFKKGTTLVKsntkdvnsngencinynmfdqsdnmckr------------esgeilgeglvdkerkialei--------------nqselnelds-kvkeqasa----HNILVLHC-----------DIVK--DSFWELVS-----    71032267        Theileria parva strain Muguga                           eukaryota>alveolata>apicomplexa  THG1\_Lsal\_225712230                                   KAHCKFEYVRQFETKET--LLP-NTWAVVRVDGRGFHA-FANKHEFTKP---NDVRALNLMNAAAKVVLGA--F-TDIVLGYGQSDEYSFVFRKS----------------ANLFSRRSAKIVTSVASLFASNYVYLWPEYFPDTKLKF--------------------------------------APSFDGRCVCYPS--DQNIRDYLSWRQ-ADC--HINNLYNTVFWALVQQGGMTNKEAQERLKGTLSADKNEILYSQFQINYNNES-QQFRKGSLLLK----------------------------------------kkvsvpvevhegnnassds----------------knedpstpskhqksgcrdr----VKIFDLNV-----------DIIG--DEFWENNEYIYS-    225712230       Lepeophtheirus salmonis                                 eukaryota>metazoa>crustacea  THG1\_Lsal\_225712866                                   KAHCKFEYVRQFETKET--LLP-NTWAVVRVDGRGFHA-FANKHEFTKP---NDVRALNLMNAAAKVVLGA--F-TDIVLGYGQSDEYSFVFRKS----------------ANLFSRRSAKIVTSVASLFASNYVYLWPEYFPDTKLKF--------------------------------------APSFDGRCVCYPS--DQNIRDYLSWRQ-ADC--HINNLYNTVFWALVQQGGMTNKEAQERLKGTLSADKNEILYSQFQINYNNES-QQFRKGSLLLK----------------------------------------kkvsvpvevhegnnass------------------dsknedpsk-hqksgcrdr----VKIFDLNV-----------DIIG--DEFWENNEYIYS-    225712866       Lepeophtheirus salmonis                                 eukaryota>metazoa>crustacea  MCYG\_00432\_Mcan\_238837882                             MANSKYEYVRNFEQSDV--LLP-NTWIVIRIDGRGFHK-LSDKYHFQKP---NDRRALDLMNSAAQAVMRD--I-PDLIMAYGVSDEFSFVFHPN----------------CQLFERRSSKLVSTIVSTFTAHYAFKWISFFPDTPLEPTF------------------------------------LPTFDGRAVQYPS--VKNLRDYMSWRQ-VDC--HINNLYNTTFWNMVQKGGMSNQDAEQELKGTVSSDKNEILFSRYGINYNNEL-EIFKKGSVLFR------------------dyeleq----------------vkrtpasiantnddrkw------------------envelsktqlekhqklrrk----ANVAIAHV-----------DIIK--DEFWEQRPWLLS-    238837882       Microsporum canis CBS 113480                            eukaryota>fungi>ascomycota  PAAG\_05399\_Pbra\_226278784                             SPNLLYEYVKAFEQDDN--LLP-NTWIVVRIDGRGFHR-FSGRYHFQKP---NDERALNLMNTAACAVMKD--L-PDLIIAYGVSDEYS-------------------------------KLVTTIVSTFTAHYIYNWSSFFPSAPLEPGF------------------------------------LPTFDGRAVQYPS--VRNLRDYMSWRQ-ADC--HINNLYNTTFWNMILRGGISNTEAEKELQYEIQPQTEE-----------------EKAGDG-------------------------------------------snevesetgeei-----------------------pptemtksqlarlrkiqkk----ATIVVKHM-----------DIIK--DDFWEQRPWILS-    226278784       Paracoccidioides brasiliensis Pb01                      eukaryota>fungi>ascomycota  PABG\_05979\_Pbra\_225677608                             ---VAYEYVKAFEQDDN--LLP-NTWIVVRIDGRGFHR-FSGRYHFQKP---NDERALNLMNTAACAVMKD--L-PDLIIAYGVSDEYS-------------------------------KLVTTIVSTFTAHYIYNWSSFFPSAPLEPGF------------------------------------LPTFDGRAVQYPS--VRNLRDYMSWRQ-ADC--HINNLYNTTFWNMILQGGISNTEAEKELQGTVSGEKNEILFSRFGINYNNEP-EMYRKGSVIFRdiahlfetqyetqpqteeekagdg----------------sneveaetgeev-----------------------pptemtksqlarlrkiqkk----ATIVVKHM-----------DIIK--DDFWEQRPWILS-    225677608       Paracoccidioides brasiliensis Pb03                      eukaryota>fungi>ascomycota  AFUB\_014160\_Afum\_159131585                            MANSKYEYVKSFEQPDV--LLP-NTWIVVRIDGRGFHK-LSDRYGFIKP---NDRRALDLMNAAAVEVMKE--L-PDLCIAYGVSDEYSFVFHPS----------------CQLFERRSAKLVTTIVSTFTAHYIYHWGTYFPSTPLHPPY------------------------------------LPSFDGRAVLYPT--TRILRDYMSWRQ-VDC--HINNLYNTTFWTMVQKGGMSNTDAERELQGTVSSDKNEILFKRFGINYNNED-EMFKKGSVVYRqvgqqgclydrrltwil-----qyql--------------edpkpesksrh---gdddeapm-------------deskisraqqdklrklrrk----AQVVVDHV-----------DIIK--DEFWERRPWILS-    159131585       Aspergillus fumigatus A1163                             eukaryota>fungi>ascomycota  ACLA\_020800\_Acla\_121701067                            MANSKYEYVKAFEQPDV--LLP-NTWIVVRIDGRGFHK-LSDRYEFIKP---NDRRALDLMNAAAVEVMKE--L-PDLCIAYGVSDEYSFVFHPS----------------CQLFERRSAKLVTTIVSTFTAHYIYQWGSYFPSMPLQAPH------------------------------------LPSFDGRAVLYPT--TRILRDYMSWRQ-VDC--HINNLYNTTFWTMVQKGGMSNTDAEKELQGTVSSDKNEILFKRFGINYNNED-EMYKKGSVLYR----------------------qyql--------------edpkptsdsksgtlgddgepalv------------qeasmsrsqqdkvrklrrk----AQVVVDHV-----------DIIK--DEFWERRPWILS-    121701067       Aspergillus clavatus NRRL 1                             eukaryota>fungi>ascomycota  AFUA\_1G14630\_Afum\_70996154                            MANSKYEYVKSFEQPDV--LLP-NTWIVVRIDGRGFHK-LSDRYGFIKP---NDRRALDLMNAAAVEVMKE--L-PDLCIAYGVSDEYRSLRFSP----------------QLSVVRATQRVSHPQPERASLPRLDLLTLQTLTLGPPY--------------------------------------LPSFDGRAVLYPT--TRILRDYMSWRQ-VDC--HINNLYNTTFWTMVQKGGMSNTDAERELHGTVSSDKNEILFKRFGINYNNED-EMFKKGSVVYRqvgqqgclydrrltwil-----qyql--------------edpkpesksrh---gdddeapm-------------deskisraqqdklrklrrk----AQVVVDHV-----------DIIK--DEFWERRPWILS-    70996154        Aspergillus fumigatus Af293                             eukaryota>fungi>ascomycota  NCU02105.1\_Ncra\_85111778                              MANSKFEYVKQFEQPDS--LLP-NTWIVVRLDGRGFTK-FSTKYAFEKP---NDKRALDLMNAAARSVMSE--L-PDITIAYGVSDEYSFVFHKS----------------CTLFERRASKLVSTIVSTFTAYYIHHWPTYFVDGPPLSPP------------------------------------LPSFDGRAVCYPS--VQNLRDYMSWRQ-VDC--HINNLYNTTFWALINQGGMDGTAAELMLKGTFSADKNEILFKKFGINYNNEP-EMFKKGSVVFR---------------------nyelv--------------epgtkkvseee---aeemsssa-------------vpevksksqvekdkkvrtk----AKIVVEHL-----------DIIR--DEFWERRPWLLS-    85111778        Neurospora crassa OR74A                                 eukaryota>fungi>ascomycota  An01g12630\_Anig\_145230810                             MANSKYEYVKSFEQPDA--LLP-NTWIVVRIDGRGFHK-LSDHYGFIKP---NDRRALDLMNAAAVGVMKD--L-PDLCIAYGISDEYSFAFHPN----------------CQLFERRSAKLVTTIVSTFTAHYIYLWGTYFPDTPLQPAA------------------------------------LPSFDGRAVMYPN--SRIFRDYMSWRQ-VDC--HINNLYNTTFWTMVLQGGMDRREAELELKGTLSSDKNEILFKRFGINYNNEE-EIYKKGSVIYR---q------e-----------------------------etspl------------------------------qedtpsktqqekirklrrk----VQVVVDHV-----------DIIK--DEFWERRPWILS-    145230810       Aspergillus niger CBS 513.88                            eukaryota>fungi>ascomycota  NFIA\_010790\_Nfis\_119494994                            MANSKYEYVKSFEQPDV--LLP-NTWIVVRIDGRGFHK-LSDRYGFIKP---NDRRALDLMNAAAVEVMKE--L-PDLCIAYGVSDEYSFVFHPS----------------CQLFERRSAKLVTTIVSTFTAHYIYQWGTYFPSTPLQPPY------------------------------------LPSFDGRAVLYPT--TRILRDYMSWRQ-VDC--HINNLYNTTFWTMVQKGGMSNTDAERELQGTVSSDKNEILFKRFGINYNNED-EMFKKGSVVYR----------------------qyql--------------edpkpesksrh---gdddealv-------------desktsrsqqdklrklrrk----AQVVVDHV-----------DIIK--DEFWERRPWILS-    119494994       Neosartorya fischeri NRRL 181                           eukaryota>fungi>ascomycota  AO090003000484\_Aory\_169770385                         MANSKYEYVKAFEQPDV--LLP-NTWIVVRIDGRGFHK-LTDRYNFTKP---NDRRALDLMNAAAVEVMKD--L-PDLCIAYGVSDEYSFVFHPS----------------CQLFERRSAKLVTTIVSTFTAHYVYLWGTYFPDNPLQFPY------------------------------------LPSFDGRAVMYPA--TRNLRDYMSWRQ-VDC--HINNLYNTTFWTMVLQGGMSNTDAEQELKGTVSSDKNEILFKRFGINYNNEE-EIYKKGSVLYR----------------------qyqle-------------dikpksesksgvlaeeegnnv--------------qeakisrsqqdklrklrrk----AQVVVDHV-----------DIIK--DEFWERRPWILS-    169770385       Aspergillus oryzae RIB40                                eukaryota>fungi>ascomycota  cgd1\_1990\_Cpar\_126643977                              MACSKYEYIKTYEQPSR--IVK-NCWFVVRIDGHSFHE-FTKDHEFHKP---NDKRGLDLMSRCAENVMKN--L-GDIVISYGQSDEFSFVFRRK----------------TDLWSRKNDKILTHVVSLFTSSFIFFWDSFFPGKKLLY--------------------------------------PPTFDGRIIMYPT--DEDIRTYLSWRQ-ADC--HINNLYNTCFWSLVSINKLNEREATEKLKFTDSSYKNELLFKEFGINYNNIS-PQFRKGTTIYK---a-------------------------------------rpkekksrdeylllknndillfdkckeatietdhrdyteldkplnpiwkiddemviiSCIYKCHC-----------DIIQ--DKFWHENDHLLK-    126643977       Cryptosporidium parvum Iowa II                          eukaryota>alveolata>apicomplexa  GSPATT00002474001\_Ptet\_145533455                      MANSKFEYVKQFEQMQN--LLP-NTYIVVRIDGKGFHK-FTKCYDFEKP---NDEQGLKLMSFSACVVMET--F-PDIQIAYGQSDEFSFVLKKD----------------SELYSRRSDKIATCVCSTFTSVYTLNFEKFMKKPLQFPY-------------------------------------IPIFDARCVCYPD--LQNIRDYLSWRQ-ADC--HINNLYNTCFWGLVQKG-MNKQESEKTLAGTNAGDKNELLFSKLGKNYNNEL-EMFKKGTTIIRqptqervnlkveldvdskeqqklnkkqk------------kkqkykdlnqq-------------------------------elnqlpnqqked----LQVKEFYV-----------DIIK--DNFWIENKAYIL-    145533455       Paramecium tetraurelia strain d4-2                      eukaryota>alveolata>ciliophora  LOC100071194\_Ecab\_149726693                           MAKSKFEYVRDFEADDT--CLA-HCWVVVRLDGRNFHR-FAEKHNFAKP---NDSRALHLMTKCAQTVMQE--L-EDIVIAYGQSDEYSFVFKRK----------------TNWFKRRASKFMTHVASQFASSYVFYWRDYFEEQPLLY--------------------------------------PPGFDGRVVVYPS--NQTLKDYLSWRQ-ADC--HINNLYNTVFWALVQQSGLTPAQAQERLQGTLAADKNEILFSEFNINYNDEP-LMYRKGTVLIW-----------------------------------------------------------------qkvgevtt-kevklpaemegkkmavirtr----TKPVPLYC-----------DIIG--DAFWKEHPEILH-    149726693       Equus caballus                                          eukaryota>metazoa>vertebrata  Thg1l\_Rnor\_62078661                                   MAKSKFEYVRDFEVDDT--CLP-HCWVVVRLDGRNFHR-FAEKHNFAKP---NDSRALHLMTKCAQTVMQE--L-EDIVIAYGQSDEYSFVFRKR----------------SNWFKRRASKFMTLVASQFASSYVFYWRDYFEDQPLLY--------------------------------------PPGFDGRVVLYPS--NQTLKDYLSWRQ-ADC--HINNLYNTVFWALIQQSGLTPVQAQQRLKGTLTADKNEILFSEFHINYNNEP-HMYRKGTVLVW-----------------------------------------------------------------qkvnevrt-qeirlpaemegekmavtrtr----TKLVALNC-----------DLIG--DAFWKEHPEILE-    62078661        Rattus norvegicus                                       eukaryota>metazoa>vertebrata  Thg1l\_Mmus\_62530979                                   MAKSKFEYVRNFEVQDT--CLP-HCWVVVRLDGRNFHR-FAEEHNFAKP---NDSRALHLMTKCAQTVMEE--L-EDIVIAYGQSDEYSFVFRKK----------------SNWFKRRASKFMTLVASQFASSYVFYWRDYFEDQPLRY--------------------------------------PPGFDGRVVLYPS--NQTLKDYLSWRQ-ADC--HINNLYNTVFWVLIQQSGLTPVQAQQRLKGTLTADKNEILFSEFHINYNNEP-HMYRKGTVLVW-----------------------------------------------------------------kve-evrtqevrlpa-emegekkavartr----TRVVALNC-----------DLIG--DAFWKEHPEILA-    62530979        Mus musculus                                            eukaryota>metazoa>vertebrata  Thg1l\_Mmus\_124377988                                  MAKSKFEYVRNFEVQDT--CLP-HCWVVVRLDGRNFHR-FAEEHNFAKP---NDSRALHLMTKCAQTVMEE--L-EDIVIAYGQSDEYSFVFRKK----------------SNWFKRRASKFMTLVASQFASSYVFYWRDYFEDQPLRY--------------------------------------PPGFDGRVVLYPS--NQTLKDYLSWRQ-ADC--HINNLYNTVFWALIQQSGLTPVQAQQRLKGTLTADKNEILFSEFHINYNNEP-HMYRKGTVLVW-----------------------------------------------------------------qkveevrt-qevrlpaemegekkavartr----TRVVALNC-----------DLIG--DAFWKEHPEILA-    124377988       Mus musculus                                            eukaryota>metazoa>vertebrata  THG1L\_Btau\_122692489                                  MAKSKFEYVRDFEADDT--CLP-HCWVVVRLDGRNFHR-FAEKHSFIKP---NDSRALHLMTKCAQTVMNE--L-EDIVIAYGQSDEYSFVFKRK----------------SNWFKRRASKFMTHVVSQFASSYVFYWRDYFEDQPLLY--------------------------------------PPGFDGRVIVYPS--NQTLKDYLSWRQ-ADC--HINNLYNTVFWALVQQSGLTPLQAQERLQGTLAADKNEILFSEFNINYNNEP-LMYRKGTVLIW-----------------------------------------------------------------qkveeitt-kevklpaemegkkmavtrtr----TMVVPLHC-----------NIIG--DAFWKEHPEILD-    122692489       Bos taurus                                              eukaryota>metazoa>vertebrata  IscW\_ISCW001226\_Isca\_241096199                        MAKSKFEYVRQFEQ--DDRCLP-NCWLVVRVDGKAFHR-FSDAHNFEKP---NDKRALDLMSRCAERVMDE--F-KDICLSYGQSDEYSFVFRK----------------DSLVYNRRASKLMTSVSSLFTSAYVFHWPEYFPDSM-RY--------------------------------------PPSFDGRVVLYPS--DKNLVDYLSWRQ-ADC--HINNLYNTVFWSLVQSGGLSPKQAEERLRGTLSSDKNEILFQEFGINYNNLS-PLYRKGTVIVR-----------------------------------------------------------------------epvpdetapaeggkkpgkqkdth----FFMKNSTN-----------KIYS-----TLEMCYIIDL    241096199       Ixodes scapularis                                       eukaryota>metazoa  PANDA\_000301\_Amel\_281345114                           MAKSKFEYVRNFEADDT--CLA-HCWVVVRLDGRNFHR-FAEKHNFAKP---NDSRALHLMTKCAQTVMKE--L-EDIVIAYGQSDEYSFVFKRK----------------SNWFKRRASKFMTHVASQFASSYVFYWRDYFEDQPLLY--------------------------------------PPGFDGRVIVYPS--NQTLKDYLSWRQ-ADC--HINNLYNTVFWALVQQSGLTPVQAQERLQGTLAADKNEILFSEFNINYNNEP-LMYRKGTVLIW-----------------------------------------------------------------kvg-evttkevklpa-emegkkmemtrtr----TKPVPLHC-----------DIIG--DAFWKEHPEILD-    281345114       Ailuropoda melanoleuca                                  eukaryota>metazoa>vertebrata  THG1L\_Hsap\_89242148                                   MAKSKFEYVRDFEADDT--CLA-HCWVVVRLDGRNFHR-FAEKHNFAKP---NDSRALQLMTKCAQTVMEE--L-EDIVIAYGQSDEYSFVFKRK----------------TNWFKRRASKFMTHVASQFASSYVFYWRDYFEDQPLLY--------------------------------------PPGFDGRVVVYPS--NQTLKDYLSWRQ-ADC--HINNLYNTVFWALIQQSGLTPVQAQGRLQGTLAADKNEILFSEFNINYNNEL-PMYRKGTVLIW-----------------------------------------------------------------qkvdevmt-keiklptemegkkmavtrtr----TKPVPLHC-----------DIIG--DAFWKEHPEILD-    89242148        Homo sapiens                                            eukaryota>metazoa>vertebrata  Dyak\GE24732\_Dyak\_195474109                           MACSRFEYVKSFEQDDS--ILP-NVWIVIRIDGKKFHK-FSKTHDFEKP---NDENALNVMNSAATAVMQE--F-RDIVVAYGQSDEYSFVFRKE----------------TAAFKRRSAKLLTYVTSLFSSSYVMQWSKWMSQPLAY---------------------------------------APCFDGRVVLYPS--EQNLKDYLSWRQ-ADV--HVNNLYNTAFWKLVLEKDLTNQQAEAKLRGTFSADKNELLFQEFGINYNNLP-AMYRKGTILLR------------------------------------------------------------------------------------krvilgdksr----QAVVPLHE-----------DLIS--SQFWKEHNEILG-    195474109       Drosophila yakuba                                       eukaryota>metazoa>hexapoda  LOC479315\_Clup\_57085243                               MAKSKFEYVRNFEADDT--CLA-HCWVVVRLDGRNFHR-FAEKHNFAKP---NDSRALHLMTKCAQTVMKE--L-EDIVIAYGQSDEYSFVFKRK----------------SNWFKRRASKFMTHVASQFASSFVFYWRDYFEDQPLLY--------------------------------------PPGFDGRVIVYPS--NQTLKDYLSWRQ-ADC--HINNLYNTVFWALVQQSGLTPAQAQERLQGTLAADKNEILFSEFNINYNNEP-LMYRKGTVLIW-----------------------------------------------------------------qqkvgevttkevklpeemqgkkmavtrtr----TKPVPLYC-----------DIIG--DAFWKEHPEILD-    57085243        Canis lupus familiaris                                  eukaryota>metazoa>vertebrata  CG4103\_Dmel\_19921364                                  MACSRFEYVKSFEQDDS--ILP-NVWIVIRIDGKKFHK-FSKTHDFEKP---NDENALNVMNAAATAVMQE--F-RDIVLAYGQSDEYSFVFRKE----------------TAAFKRRSAKLLTYVTSLFSSSYVMQWSKWMNLPLAY---------------------------------------APCFDGRVVLYPS--EQNLKDYLSWRQ-ADV--HVNNLYNTAFWKLVLEKGLTNQQAEAKLRGTFSADKNELLFQEFGINYNNLP-AMYRKGTILLR------------------------------------------------------------------------------------krvilgeksr----QAVVPLHE-----------DLIS--SQFWKEHTEILG-    19921364        Drosophila melanogaster                                 eukaryota>metazoa>hexapoda  \_Hsap\_62897799                                        MAKSKFEYVRDFEADDT--CLA-HCWVVVRQDGRNFHR-FAEKHNFAKP---NDSRALQLMTKCAQTVMEE--L-EDIVIAYGQSDEYSFVFKRK----------------TNWFKRRASKFMTHVASQFASSYVFYWRDYFEDQPLLY--------------------------------------PPGFDGRVVVYPS--NQTLKDYLSWRQ-ADC--HINNLYNTVFWALIQQSGLTPVQAQGRLQGTLAADKNEILFSEFNINYNNEP-PMYRKGTVLIW-----------------------------------------------------------------qqkvdevmtkeiklptemegkkmavtrtr----TKPVPLHC-----------DIIG--DAFWKEHPEILD-    62897799        Homo sapiens                                            eukaryota>metazoa>vertebrata  LOC462226\_Ptro\_114603135                              MAKSKFEYVRDFEADDT--CLA-HCWVVVRLDGRNFHR-FAEKHNFAKP---NDSRALQLMTKCAQTVMEE--L-EDIVIAYGQSDEYSFVFKRK----------------TNWFKRRASKFMTHVASQFASSYVFYWRDYFEDQPLLY--------------------------------------PPGFDGRVVVYPS--NQTLKDYLSWRQ-ADC--HINNLYNTVFWALIQQSGLTPVQAQGRLQGTLAADKNEILFSEFNINYNNEP-LMYRKGTVLIW-----------------------------------------------------------------kvd-evmtkeiklpt-emegkkmavtrtr----TKPVPLHC-----------DIIG--DAFWKEHPEILD-    114603135       Pan troglodytes                                         eukaryota>metazoa>vertebrata  Dsim\GD21976\_Dsim\_195579310                           MACSRFEYVKSFEQDDS--ILP-NVWIVIRIDGKKFHK-FSKTHDFEKP---NDENALNVMNAAATAVMQE--F-RDIVLAYGQSDEYSFVFRKE----------------TAAFKRRSAKLLTYVTSLFSSSYVMQWSKWMNLPLAY---------------------------------------APCFDGRVVLYPS--EQNLKDYLSWRQ-ADV--HVNNLYNTAFWKLVLEKGLTNQQAEAKLRGTFSADKNELLFQEFGINYNNLP-AMYRKGTILLR-----------------------------------------------------------------krvil-------------------geksr----QAVVPLHE-----------DLIS--SQFWKEHTEILG-    195579310       Drosophila simulans                                     eukaryota>metazoa>hexapoda  LOC100075219\_Oana\_149412544                           MAKSKFEYVRAFEADDT--CLP-HCWVVIRLDGRNFHR-FADQHNFAKP---NDDRALHLMNKCAQVVMQE--L-EDIVIAYGQSDEYSFVFKKM----------------SNWFKRRASKFMTHVASQFASSYVFYWKDYFKDQPLLY--------------------------------------PPGFDGRVVLYPS--NQNLKDYLSWRQ-ADC--HINNLYNTVFWMLVQRSGLTPVQAQERLKGTLAADKNEILFSEFNINYNNES-PMYRKGTVLIW-----------------------------------------------------------------qkvsevtt-keskqpedkeeqkvevtrtr----TKVVPLHC-----------DIIG--DAFWKEHQEILA-    149412544       Ornithorhynchus anatinus                                eukaryota>metazoa>vertebrata  \_Hsap\_7020726                                         MAKSKFEYVRDFEADDT--CLA-HCWVVVRLDGRNFHR-FAEKHNFAKP---NDSRALQLMTKCAQTVMEE--L-EDIVIAYGQSDEYSFVFKRK----------------TNWFKRRASKFMTHVASQFASSYVFYWRDYFEDQPLLY--------------------------------------PPGFDGRVVVYPS--NQTLKDYLSWRQ-ADC--HINNLYNTVFWALIQQSGLTPVQAQGRLQGTLAADKNEILFSEFNINYNNEP-PMYRKGTVLIW-----------------------------------------------------------------qkvdevmt-keiklptemegkkmavtrtr----TKPVPLHC-----------DIIG--DAFWKEHPEILD-    7020726         Homo sapiens                                            eukaryota>metazoa>vertebrata  LOC715853\_Mmul\_109079563                              MAKSKFEYVRDFEADDT--CLA-HCWVVVRLDGRNFHR-FAEKHNFAKP---NDSRALHLMTKCAQTVMEE--L-EDIVIAYGQSDEYSFVFKRK----------------TNWFKRRASKFMTHVASQFASSYVFYWRDYFEDQPLLY--------------------------------------PPGFDGRVVVYPS--NQTLKDYLSWRQ-ADC--HINNLYNTVFWALIQQSGLTPVQAQGRLQGTLAADKNEILFSEFNINYNNEP-LMYRKGTVLIW-----------------------------------------------------------------qkvdevmt-keiklptemegkkmavtrtr----TKPVPLHC-----------DIIG--DAFWKEHPDILD-    109079563       Macaca mulatta                                          eukaryota>metazoa>vertebrata  LOC100025355\_Mdom\_126290678                           MAKSKFEYVRDFEADDT--CLA-HCWVVVRLDGRNFHR-FAEKHNFTKP---NDSRALDLMTRCAQTVMTE--L-EDIVMAYGQSDEYSFVFKRK----------------SNWFRRRASKFMTNAASQFASSYVFYWKDYFKDQDLLY--------------------------------------PPAFDGRVVVYPS--NQTIKDYLSWRQ-ADC--HVNNLYNTVFWMLIQRSKLTPAQAQERLRGTLAADKNEILFSEYNINYNNEP-PMFRKGTVLIW-----------------------------------------------------------------qkkikeikskeikppgetegkqvevtrir----TKPVALHC-----------DIIG--DTFWKEHPEILE-    126290678       Monodelphis domestica                                   eukaryota>metazoa>vertebrata  LOC100353509\_Ocun\_291387726                           MAKSKFEYVRDFEADDT--CLA-HCWVVVRLDGRNFHR-FSEKHNFAKP---NDSRALHLMTKCAQTVMEE--L-EDIVIAYGQSDEYSFVFKRK----------------SNWFKRRASKFMTHVASQFASSYVFYWRDYFEDQPLLY--------------------------------------PPGFDGRVVVYPS--NQTLKDYLSWRQ-ADC--HINNLYNTVFWALVQQSGLTPVQAQGRLQGTLAADKNEILFSEFNINYNNEP-LMYRKGTVLIW-----------------------------------------------------------------qkvgevtt-kevklpaemegkkmavtrtr----SKPVALHC-----------DLIG--DAFWKEHPEILS-    291387726       Oryctolagus cuniculus                                   eukaryota>metazoa>vertebrata  Dsec\GM14588\_Dsec\_195338513                           MACSRFEYVKSFEQDDS--ILP-NVWIVIRIDGKKFHK-FSKTHDFEKP---NDENALNVMNAAATAVMQE--F-RDIVLAYGQSDEYSFVFRKE----------------TATFKRRSAKLLTYVTSLFSSSYVMQWSKWMNLPLAY---------------------------------------APCFDGRVVLYPS--EQNLKDYLSWRQ-ADV--HVNNLYNTAFWKLVLEKGLTNQEAEAKLRGTFSADKNELLFQEFGINYNNLP-AMYRKGTILLR------------------------------------------------------------------------------------krvilgeksr----QAVVPLHE-----------DLIS--SQFWKEHTEILG-    195338513       Drosophila sechellia                                    eukaryota>metazoa>hexapoda  THG1\_Ccle\_225719698                                   MAHSKFEYVRSFESKDS--LLP-NTWLVVRIDGRGFHA-FSNEHDFEKP---NDVRALNLMNAAAKVVIEA--F-TDTVLAYGQSDEYSFVFRRN----------------TNLYSRRSAKIATNVTSLFAANYVYLWPQFFPDKPLKV--------------------------------------APSFDGRCVCYPT--DANLRDYLSWRQ-ADC--HINNLYNTVFWALVLKGGLTNREAQERLKGTLSGDKNEILFSQFQINYNQEA-QQFRKGSTLLK----------------------------------------kapvpveipqdkked-------------------------spkrqkensgfrdr----VKIFDLNI-----------DLIG--DDFWKENAHIYS-    225719698       Caligus clemensi                                        eukaryota>metazoa>crustacea  LOC100117826\_Nvit\_156547518                           MAKSKYDYVKDFEHEDN--CLP-NCWIVVRIDGRNFSK-FADSHQFVKP---NDLAALELMNRAAMTVMED--F-REIVIAYGQSDEYSFVFRKD----------------TQLFKRRASKLMSNVNSLFASAYVYNWPRFFKNRELHY--------------------------------------PPSFDARVVLYPT--DKNLRDYLAWRQ-ADV--HINNLYNTCFWSLVLKKHLTPQQAEERLSGTLSSHKNELLYQEFGINYNNEP-AVYRKGTTLLR-----------------------------------------------------------------------------------klvahgngrlk----PTVVPLVD-----------DIIG--DRFWKENPEVIG-    156547518       Nasonia vitripennis                                     eukaryota>metazoa>hexapoda  LOC692885\_Bmor\_114051932                              MAKSSFEYVKKFEHDDS--LLP-NTWIVIRLDGKCFHK-FADDHNFTKP---NDLRALKLMNYAAYTVLKE--Y-SDILLSFGQSDEYSFVLRKD----------------SCLYKRRSAKLLTTINSKFSSSYVFYWNKFFEHLPLKY--------------------------------------PPCFDGRIVLYPC--DENLIDYMKWRQ-ADV--HINNLYNTTFWTLILKGQLTPVQAEKRLSGTVSADKNEILFQEFNMNYNNEP-EIFKRGTILLR-----------------------------------------------------------------------------------ksilh-nkvnk----SIIVDVHD-----------DMLK--DKFWKENIYILS-    114051932       Bombyx mori                                             eukaryota>metazoa>hexapoda  SJAG\_04233\_Sjap\_213409163                             MANSRFEYVKGFEKADN--LLP-ETYIVIRIDGKGFHK-FTEKHEFAKP---NDSRCLELMNKAAEIVVSE--F-TDIVLAYGDSDEYSFVWCKG----------------TQLYERRESKLVSHVCSLFTSAFVFNWSKFFDIPLRS---------------------------------------LPSFDGRAVLYPS--FSSLRDYLSWRQ-ADC--HINNLYNTTFWALRLQGKMSNREAEERLKGTVSADKHEILFSQFGINYNNEP-EMYKKGTIFTRpadgddmls-------------------------------------------------------------------------kgtnlskkqkkk----LVIEKLHV-----------DLIA--DSFWKERPYLET-    213409163       Schizosaccharomyces japonicus yFS275                    eukaryota>fungi>ascomycota  thg1\_Ddis\_66802352                                    MANSKYEYVKSFEQPDI--LLQ-NVWIVVRIDGRSFHK-FTTKHDYAKP---NDDRGLSLMNRAALEVCKE--F-PDIVIAFGESDEYSFVLKKS----------------CNLFERRSSKISSSIVSYFTSQFVYRWKEYFGEHELKY--------------------------------------PPTFDSRCVLYPT--DENIKDYLSWRQ-ADT--HINNLYNTCYWALVLKAGKTPIEAENELRGTFSDGKNEMLFSRFNINYNNLP-AEYRKGSVIFK------------------------------------------------------------------------------kpvqetnketgltksk----KRLVIEHV-----------DIIS--EKFWKEYPDILA-    66802352        Dictyostelium discoideum AX4                            eukaryota>amoebozoa>mycetozoa>dictyosteliida  LOC100205752\_Hmag\_221135876                           MAKSKYEYVKKFEQNET--CLL-NCWIVVRIDGRGFHK-FTHDHLYEKP---NDIRGLSLMNFCAKEVMKQ--F-QDIVISYGQSDEYSFVFSKN----------------TSQFKRRSCKLMSNIVSLFSSSFVFYWKTFFLNDLIY---------------------------------------PPQFDGRIILYPS--LENIRDYLSWRQ-ADC--HINNLYNTCFWSLVNKGGLSTLDAELKLKGTLAKDKNELLFSEFDVNYNDIS-PIFRKGNIVIR------------------------------------------------------------------------qkvaeeimkekngesvkvikek----NDTVILHD-----------DIIG--ENFWKKFPEILT-    221135876       Hydra magnipapillata                                    eukaryota>metazoa>cnidaria  \_Gmor\_289742577                                       MACSRYEYVKSYEVQDV--LLP-NVWIIIRIDGKGFHK-FCKAHDFEKP---NDERALNLMTSAAQTVMEK--F-SDIILSYGQSDEYSFVFRKE----------------TNVYNRRSSKLLSYVTSLFTSCYVMYWPQWMGNQKLTY--------------------------------------PPSFDGRVVLYPS--DQNLRDYLSWRQ-ADV--HINNLYNTAFWNLVQKKGLTNREAEAKLRGTYAAYKNELLFSEFGINYNNLP-GMFRKGTILLR----------------------------------------------------------------------------------kqvnrtkheisk----QLIVPFHE-----------DMIS--DIFWKKHSELLA-    289742577       Glossina morsitans morsitans                            eukaryota>metazoa>hexapoda  Dere\GG24229\_Dere\_194857325                           MACSRFEYVKSFELDDS--ILP-NVWIVIRIDGKKFHK-FSKAHDFEKP---NDENALNVMNAAATAVMQE--F-RDIVLAYGQSDEYSFVFRKE----------------TAAFKRRAAKLLTYVTSLFSSSYVMQWPKWMQLPLAY---------------------------------------APCFDGRVVLYPS--EQNLKDYLSWRQ-ADV--HVNNLYNTAFWKLVLEKGLTNQQAEAKLRGTFSADKNELLFQEFGINYNNLP-AMYRKGTILLR------------------------------------------------------------------------------------krvilgeksr----QAVVPLHE-----------DLIS--SQFWKKHTEILG-    194857325       Drosophila erecta                                       eukaryota>metazoa>hexapoda  TVAG\_470220\_Tvag\_123437995                            MACSKWEYVKDFELDDR--LLP-STYIVVRVDGRGFTE-FCINHNLEKP---LDDRLIRLMSNCAQKVMLK--F-DEMVLAFGESDEFSFIFKKS----------------AKVFNRRRDKINSTVASLFSSIFVKDWSNFFPNLPLQD--------------------------------------PPSFDSRIVLYPS--LDVVKDYLCWRQ-ADT--HINCLYNYTLNVLL-RAGENPTDATEKLRGTFSNDKNEILFKH-GINYKLLP-AAHRKGTVWIH--------------------------------------------------------------------------------------------ak-----KLFETND-----------DLIQ--DAFWKKYSKLFE-    123437995       Trichomonas vaginalis G3                                eukaryota>parabasalia  LOC100374565\_Skow\_291224944                           MANSKYEYVRHFETQDR--CLP-NCWIVVRLDGKNFHK-FSDSHEFSKP---NDEAALNLMNCCAVYVMNE--F-QDITVAYGESDEYSFVFRKG----------------TTQFSRRASKLMTNVVSLFAASYVFNWSKFFPNKQLMY--------------------------------------PPAFDCRVVVYPS--DENLRDYLSWRQ-ADC--HINNLYNTCFWKLVLQGGYSTKEAEQKLKGTYSSDKNELLFSQFDINYNELP-QLFRKGTVLFWqkveeklm------------------------------------------------------------------khfkskdserletkevtrvr----NVVVTQHI-----------DIIG--DEFWKLHPEILG-    291224944       Saccoglossus kowalevskii                                eukaryota>metazoa>hemichordata  Dana\GF14997\_Dana\_194758495                           MACSRFEYVKSFEQDDS--ILP-NVWIVIRVDGKKFHK-FSKTHDFEKP---NDENALNVMNAAATAVMQE--F-RDIVLAYGQSDEYSFVFRKE----------------TTAFKRRSAKLLTYVTSLFSSSYVMQWPRWKSVPLAY---------------------------------------APCFDGRVVLYPS--DENLRDYLSWRQ-ADV--HVNNLYNTAFWKLVLGKGLSNQQAEERLRGTFSADKNELLFQEFGINYNTLP-AMYRKGTILLR-------------------------------------------------------------------------------------krvvdrkgr----QAIVPLHE-----------DLIS--SQFWKNHTEILG-    194758495       Drosophila ananassae                                    eukaryota>metazoa>hexapoda  Dmoj\GI17333\_Dmoj\_195115597                           MACSRYEYVKSYEQDDK--ILP-NVWIVIRVDGKKFHK-FANAHKFEKP---NDENALNVMNAAGIAVMEE--F-RDIVLGYGQSDEYSFVFRKD----------------TSAFKRRAAKLLSYVTSMFTSSYVLSWSQWMQRPLSY---------------------------------------APCFDGRIVLYPS--DENLRDYLSWRQ-ADV--HVNNLYNTAFWKLVLESGLTNQQAEERLRGTLSSDKNELLFQEFGINYNTLP-AMYRKGTILLR--------------------------------------------------------------------------------krvvigsqdeqkgr----QAIVPIHE-----------DLIG--NEFWKQHTEILG-    195115597       Drosophila mojavensis                                   eukaryota>metazoa>hexapoda  Dwil\GK18307\_Dwil\_195437103                           MACSRYEYVKSYEQDDS--ILP-NVWIVIRIDGKKFHK-FSKIHDFEKP---NDENALNLMNAAAIAVMEE--F-RDIVLAYGQSDEYSFVFRKE----------------TQAFKRRSAKLLTYVTSLFSTNYVMQWPQWMKDRKLNY--------------------------------------APCFDGRVVLYPS--DENLRDYLSWRQ-ADV--HVNNLYNTAFWKLVLDKGLSNQQAEERLRGTFSADKNELLFQEFGINYNNMP-AMYRKGTILMR--------------------------------------------------------------------------------krlelp--qgdktr----QVIVPLHD-----------DLIS--SKFWKTHTELLG-    195437103       Drosophila willistoni                                   eukaryota>metazoa>hexapoda  Dper\GL25730\_Dper\_195155911                           MACSRYEYVKTYEQDDT--ILP-NVWIVIRIDGKKFHK-FSKTHNFEKP---NDENALNVMNAAATAVMQE--F-RDVVLAYGQSDEYSFVFRKE----------------TAAFKRRSAKLLTYVTSLFSTSYVMQWSKWMCLPLAY---------------------------------------APCFDGRVVLYPS--DENLRDYLSWRQ-ADV--HVNNLYNTAFWKLVLDSGLSNQKAEERLRGTFSADKNELLFQEFGINYNNLP-AMYRKGTILLR--------------------------------------------------------------------------------krvisdgdndqkgr----QAIVPLHE-----------DLIS--SHFWKVHTEILG-    195155911       Drosophila persimilis                                   eukaryota>metazoa>hexapoda  Dpse\GA17959\_Dpse\_125987191                           MACSRYEYVKTYEQDDT--ILP-NVWIVIRIDGKKFHK-FSKTHNFEKP---NDENALNVMNAAATAVMQE--F-RDVVLAYGQSDEYSFVFRKE----------------TAAFKRRSAKLLTYVTSLFSTSYVMQWSKWMSLPLAY---------------------------------------APCFDGRVVLYPS--DENLRDYLSWRQ-ADV--HVNNLYNTAFWKLVLDSGLSNQKAEERLRGTFSADKNELLFQEFGINYNNLP-AMYRKGTILLR--------------------------------------------------------------------------------krvisdgdndqkgr----QAIVPLHE-----------DLIS--SHFWKVHTEILG-    125987191       Drosophila pseudoobscura pseudoobscura                  eukaryota>metazoa>hexapoda  BBOV\_IV004960\_Bbov\_156086032                          MANTRFSYVKHFEQDTI--LLP-ESWPVVRVDGRGFTK-FSKLHEFRKP---NEPLALGVMNAAAAHVMST--F-DDIVLAYGHSDEYR-------------------------------KILSSVVSAFSSAFSFYWSRFYPDRQLKI--------------------------------------LPSFDGRIVLYPR--FENIVDYFSWRH-ADC--HINNQYNICFWCLVAD-GKCPDEAYKWLKHTQKGEKNEYIYQSRGINYNNLP-RIFRKGTTLVRllstepglgqcvqdgittkvdrgsidcqatd---------rhrldtsivntrqqatgdvielti------------deseialisqkldsicnp----MGIGVVHC-----------DNTS--EAFWNAAA-----    156086032       Babesia bovis T2Bo                                      eukaryota>alveolata>apicomplexa  PAS\_chr1-4\_0323\_Ppas\_254566669                        MANSKFEYVKAFEKEVY--LLP-ETYMVVRVDGRGFHK-LSETYKFEKP---NDLNALELMNKAAQSVMEK--I-PEALLAYGDSDEYSFLLQKN----------------CEIFERREAKLTSTFSSTFTAYYNFYWKDYFPNSPLTPER------------------------------------LPTFDARVVLYPN--QQNVKDYFSWRQ-VDC--HINNLYNTTFWQLILKKGLTPQESEKRLMGTLSSDKNEILFTELGINYNDEA-EIYKKGTVLIRewelkddqpslnerels----------------------------------------------------------------krqkdrlrkkfrk----AKIQKYHI-----------DIIN--EGFWNERPWLLE-    254566669       Pichia pastoris GS115                                   eukaryota>fungi>ascomycota  DEHA2F22880g\_Dhan\_294659006                           MAKSRFEYVKQFERENF--LLP-DTYLIVRVDGKGFHK-FSEEYEFSKP---NDIRALKVMNNAAKNLMAQ--F-PDIMMAYGDSDEYSFLLRRK----------------CSLFERREMKLVSTFASFISVNYLYEWNLEFPEKQIRLER------------------------------------LPTFDARIVVYPT--IKHIRDYFSWRQ-VDC--HINNLYNTTFWTLVIKGGMTGREAENKLLGTVSSDKNEMLFKEFGINYNNES-EIFKKGTILVReydytr-----egddls----------------------------------------------------------------krqqqrvekqrkk----ASIEEYHL-----------DIIG--DTFWNERPWLLE-    294659006       Debaryomyces hansenii CBS767                            eukaryota>fungi>ascomycota  DEHA0F24321g\_Dhan\_50425487                            MAKSRFEYVKQFERENF--LLP-DTYLIVRVDGKGFHK-FSEEYEFSKP---NDIRALKVMNNAAKNLMAQ--F-PDIMMAYGDSDEYSFLLRRK----------------CSLFERREMKLVSTFASFISVNYLYEWNLEFPEKQIRLER------------------------------------LPTFDARIVVYPT--IKHIRDYFSWRQ-VDC--HINNLYNTTFWTLVIKGGMTGREAENKLLGTVSSDKNEMLFKEFGINYNNES-EIFKKGTILVReydytr-----egddls----------------------------------------------------------------krqqqrvekqrkk----ALIEEYHL-----------DIIG--DTFWNERPWLLE-    50425487        Debaryomyces hansenii CBS767                            eukaryota>fungi>ascomycota  GSTEN:00031660:G:001\_Tnig\_47216813                    ----FEY-VRNFETDDS--CLR-NCYIVVRLDGRNFHK-FAEHHTFLKP---NDGRALGLMTHSARCVMAE--LE-DVVIAYGQSDEFSFVFKKT----------------STLFKRRASKLMTHVVSQFSSSYVFYWREFFGDQPLLY--------------------------------------PPGFDGRVVLYPS--NRNLRDYLSWRQ-AD-----------------------SATEENSCEGTLAADKNEILFSEFNVNYNNE--AVHRKGTILIW----------------------------------------erqeetvkkrtkspreeke--------------------------tdatvtrsr----RRVQEHHC-----------DIIG--EQFWQEHPDILE-    47216813        Tetraodon nigroviridis                                  eukaryota>metazoa>vertebrata>actinopterygii  THG1\_Crei\_158283078                                   MANSKYEYVKQYELDDS--LLP-GCWIVVRIDGKGFTK-FSELHGFEKP---NDKRALDLMDECAKEVLNE--F-PDVRLAYGESDEYSFVLGRS----------------TDMYGRRASKIVSLLVSCFTANYVAKWAAFLPDTPLRS--------------------------------------TPMFDGRAVCYPL--DSNLRDYLSWRQ-ADT--HINNQYNTCFWALV-KSGKTPTEAQATLRGTQTAQKNELLFNEFGINYAHLP-EQFKKGSVVIR----------------------------------------rktmvlqdgev----------------------------------kqradgt------NLPTVLHT-----------DIIR--EEFWQEYPHLMA-    158283078       Chlamydomonas reinhardtii                               eukaryota>viridiplantae>chlorophyta  Dvir\GJ16007\_Dvir\_195385629                           MACSRYEYVKSYEQDDR--ILP-NVWIVIRVDGKKFHK-FANVHKFEKP---NDENALNVMNAAGIAVMQE--F-RDIVLGYGQSDEYSFVFRKD----------------TTAFKRRAAKLLSYVTSMFSSSYVLSWPQWMQQPLSY---------------------------------------APCFDGRIVLYPS--DENLRDYLSWRQ-ADV--HVNNLYNTAFWKLVLESGLTNQQAEERLRGTLSADKNELLFQQFGINYNNLP-AMYRKGTILLR----------------------------------------krlleqe--------------------------------------kgrpvlrer----QAIVPIHE-----------DLIG--AAFWQKHTEILG-    195385629       Drosophila virilis                                      eukaryota>metazoa>hexapoda  AT2G32320\_Atha\_238479416                              IGKIEPDYVKSFQFESR--LLP-LTWVVVRIDGCHFHR-FSEVHEFEKP---NDEQALKLMNSCAVAVLEE--F-QDIAFAYGVSDEFSFVLKNK----------------SELYKRQSSKIISAVVSFFTSTYMMRWGDFFPHKKLKY--------------------------------------PPSFDGRAVCYPT--SDILLDYLAWRQ-VDC--HINNQYNTCFWMLV-KSGKSKIQAQDYLKGTQTREKNELLSQQFGIEYNSLP-VIFRMGSSVFR---------------------------------------lktqegvteen-----------------------------------gevsgkqve----AEVGVDYS-----------NIID--QCFWQQHPHILS-    238479416       Arabidopsis thaliana                                    eukaryota>viridiplantae  TSTA\_094890\_Tsti\_242778279                            MANSKYEYVRLFEQPDN--LLP-ETWIVVRIDGRGFHK-LSDKYKFEKP---NDRRALDLMNAAAVSVMKD--L-PDLIIAYGVSDEYSFVFHPN----------------CQLFERRSAKLVTTIVSTFTASYVCQWPIFFPDKPLDLSS------------------------------------LPTFDGRAVQYPN--AKILRDYMSWRQ-VDC--HINNLYNTTFWSLVLKGGMSNVEAEKELQGTVSSDKNEILFSRFGINYNNEA-EIYKKGSVVYR------------------------------------qyqledqfsattasltqaaesq-----------------psgelsktqqekmrklrrk----AQVVVEHV-----------DIIK--DDFWQRRPWLLS-    242778279       Talaromyces stipitatus ATCC 10500                       eukaryota>fungi>ascomycota  AaeL\_AAEL014040\_Aaeg\_157138643                        MAKSRFEYVKSFEQSDT--LLR-NCWIVVRVDGKGFHK-FCDVHGFEKP---NDNRGLNLMSLAAVNVMQE--F-NEIVIAYGQSDEYSFVFKRD----------------SMVYERRRDKLVSYVASLFTSAYIFNWGYIFKDTVPLKY-------------------------------------PPVFDARAVLYPT--DQNLRDYLSWRQ-ADV--HINNLYNTAFWNLV-ASGLTNAEAENRLRGTLSSDKNELLFSEFNINYNNEP-AMFRKGTVLLK----------------------------------------kktav-------------------------------------------adnksl----SLIVPIFD-----------DMIG--DKFWQTHPELLD-    157138643       Aedes aegypti                                           eukaryota>metazoa>hexapoda  THG1\_Crog\_225711510                                   MAHSKFEYVRGFESKDT--LLP-NTWLVVRIDGRGFHS-FSDRHDFVKP---NDARSLDLMNAAAKVVMKA--F-PETVLAYGQSDEYSFVFRRN----------------TNLYSRRASKIVTNVTSLFAANYVYLWPEFFPDTKLKY--------------------------------------APSFDGRCVTYPT--DQNLRDYLSWRQ-ADC--HINNLYNTVFWALVQEGGLSNQKAQERLKGTLSGDKNEILFSQFNINYNEEP-QQFRKGSILLK----------------------------------------kkvsvpiegsapvaeeeekqp--------------egggskakr-kphsgtrer----VQVFDLNV-----------DMIG--DEFWRENSHIYT-    225711510       Caligus rogercresseyi                                   eukaryota>metazoa>crustacea  OsJ\_19344\_Osat\_222632362                              -EKCRYENVKKFDINHR--LPP-CNWTVVRIDICKFEQ-FSLIHSFDKP---NDEAALRLMNASASLMMES--F-PDIVFGYGFSNEYSFVFQDK----------------TELYQRQESLILSSCTSRFTLFYMMKWKDFFPNKDLVE--------------------------------------PPHFEAELLCYPK--QKILCDYLSSRQ-AEC--HTTNQYSTCFWMLV-KSGKSENEAREILKGTLSKDKNELLFQQFHLNYNNEP-AVFRKGSCTYR----------------------------------------qkveesadae-----------------------------------grenttrer----WDVIVAHA-----------D-MG--TEFWRKHPYILR-    222632362       Oryza sativa Japonica Group                             eukaryota>viridiplantae  OsI\_20769\_Osat\_218197162                              -EKCRYENVMKFDINHR--LPP-CNWTVVRIDICKFEQ-FSLIHSFDKP---NDEAALRLMNASASLMMES--F-PDIVFGYGFSNEYSFVFQDK----------------TELYQRQESLILSSCTSRFTLFYMMKWKDFFPNKDLVE--------------------------------------PPHFEAELLCYPK--QKILCDYLSSRQ-AEC--HTTNQYSTCFWMLV-KSGKSENEAREILKGTLSKDKNELLFQQFHLNYNNEP-AVFRKGSCTYR----------------------------------------qkveesadae-----------------------------------grenttrer----WDVIVAHA-----------D-MG--TEFWRKHPYILR-    218197162       Oryza sativa Indica Group                               eukaryota>viridiplantae  Dgri\GH10821\_Dgri\_195030226                           MACSRYEYVKGYEQDDR--ILP-NVWIVIRIDGKKFHK-FANTHKFEKP---NDENALHVMNVAGIGVMQE--F-RDIVLGYGQSDEYSFVFRKD----------------TDAFKRRAAKLLTYVTSLFSTSYVMSWPKWMQQPLAY---------------------------------------APCFDGRIILYPS--DENLRDYLSWRQ-ADV--HVNNLYNTAFWKLVLESGLSNQQAEQRLRGTLSADKNELLYQQFGINYNNMP-AMYRKGTILLR----------------------------------------krvlqfgqd---------------------------------------eqdkgr----QAIVPLHE-----------DLIG--AEFWRKHPEILG-    195030226       Drosophila grimshawi                                    eukaryota>metazoa>hexapoda  Os05g0535500\_Osat\_115465113                           -EKCRYENVKKFDINHR--LPP-CNWTVVRIDICKFEQ-FSLIHSFDKP---NDEAALRLMNASASLMMES--F-PDIVFGYGFSNEYSFVFQDK----------------TELYQRQESLILSSCTSRFTLFYMMKWKDFFPNKDLVE--------------------------------------PPHFEAELLCYPK--QKILCDYLSSRQ-AEC--HTTNQYSTCFWMLV-KSGKSENEAREILKGTLSKDKNELLFQQFHLNYNNEP-AVFRKGSCTYR----------------------------------------qkvee------------------------------sadae-----grenttrer----WDVIVAHA-----------D-MG--TEFWRKHPYILR-    115465113       Oryza sativa Japonica Group                             eukaryota>viridiplantae  CNC02800\_Cneo\_58265566                                MAKSRFEYVKKFELPDP--LVP-NTYIIVRIDGKGFHK-FSDVHSFDKP---NDIRALKLMNTAAKAVLNE--Y-KDVVMAFGESDEYSFLLRRT----------------TTLYNRRRSKINSSIVSLFTSAYVFHWASFFPNTPLLY--------------------------------------PPSFDARVVLYPN--VKEVRDYFSWRQ-ADT--HINNLYNTTFWALV-HDGLTTAEANKALQGTNSKDKNEILFTKFGINYNTLP-EMFRKGSVCVR------------------slsleepqgslaeqqaahgimtlsivpstsg--------------------------nsntils--qkekvyqgte----GSPMVLHM-----------DIIN--DIFWSERPWLLS-    58265566        Cryptococcus neoformans var. neoformans JEC21           eukaryota>fungi>basidiomycota  PICST\_60761\_Psti\_150865885                            MAKSRFEYVKQFERENY--LLP-DTYIVIRVDGKGFHK-FSQEYEFDKP---NDIRALNVMNRAAQAVVES--Y-SDVLMAYGDSDEYSFLLRKN----------------CQLYERREMKLITMFSSMISTNYFYFWNEEFPEKKLKQSR------------------------------------LPNFDARAVLYPN--FALIKDYFSWRQ-VDC--HINNLYNTTFWALVLKGGMTPQEAENRLIGTVASDKNEILFSQFGINYNNEP-EIFKKGTIIMR----------------------------------------eldeedsr---------------------------dekelsarqkqridkkrkk----AEIKLLHE-----------DLIT--ETFWSSRPWLSS-    150865885       Pichia stipitis CBS 6054                                eukaryota>fungi>ascomycota  MONBRDRAFT\_31372\_Mbre\_167518403                       MANSRFQYVRGFESEAR--LLP-NTWIVVRIDGRGFHK-FSAKHGFKKP---NDARAINLMNAAAVACMNE--F-PDIVMAYGESDEYSFVFDKR----------------TQLFSRRGDKIMSSIVSFFSSTYVMQWPQHMVDAEGQPEPLVA---------------------------------TPHFDGRCVLYPT--LENLRDYVAWRQ-VDC--HINNLYNTTFWTLVLKGGLTEYEAEQRLVGTFSKDKNELLFSEFGINYNEEP-LMFRKGSVLHW----------------------------------------rripvtvtkegpts---------------------kadptpvv--r--tvtrqk----LTVIVDHC-----------DLLQ--EEFWTEHADVFT-    167518403       Monosiga brevicollis MX1                                eukaryota>choanoflagellida  THG1\_Afim\_229366028                                   MAKSKFEYVRNFETDDS--CLR-NCYIVVRLDGRNFHK-FAEQHKFLKP---NDNRALGLMTRSARSVMED--L-EDIIIAYGQSDEFSFVFKRT----------------STLFKRRASKLMTHVASQFSSSYVFYWKEFFGEQPLLY--------------------------------------PPGFDGRVVLYPS--NHNLRDYLSWRQ-ADC--HINNLYNTVFWTLVQNGGLTTAQAEERLKGTLAADKNEILFSELDINYNTES-AVHRKGTTLIW---------------------------------------ekrdetti----------------------------krtklpkgeetemavtrsr----RRVHAHHC-----------DVIG--EQFWTEHPDILE-    229366028       Anoplopoma fimbria                                      eukaryota>metazoa>vertebrata>actinopterygii  LOC587315\_Spur\_115921197                              MAKSKFEYVRQFETQDK--LLP-NSWIVIRIDGKNFHK-FSDIHGFEKP---NDLRGLSLMSEAATSVMTE--F-KDIILAYGQSDEYSFVMKKD----------------TTQYGRRASKLATTVSSLFASSFVFLWPKFFPDTPLQY--------------------------------------PPAFDGRCVLYPS--NKNLRDYLSWRQ-ADC--HINNLYNTCFWTLTQRGNCSPKEAEERLKGTVSGDKNELLFSQFDINYNAEP-EIFRKGTTLMS----------------------------------------ntveeevlcg-------------------------qgtefek------tvsktr----RTVNKIHK-----------DIIG--NSFWTEHPELLG-    115921197       Strongylocentrotus purpuratus                           eukaryota>metazoa>echinodermata  TRIADDRAFT\_32525\_Tadh\_190579964                       MAKSRYEYVKLFEQADQ--CLR-NCWIVVRLDGRCFHK-FSSQHRFKKP---NDDRALNLMNHCGKAVMKE--F-PDIILGYGQSDEFSFIFKKS----------------CNLFGRRASKLMTNVTSLFSSSYVFYWKNYFNDILQY---------------------------------------PPTFDGRVVLYPS--DKNLRDYLSWRQ-ADC--HINNLYNTSFWALVQQGQYSLPDAEKKLCGTDSGDKNELLFSQFQINYDKEP-AIFRKGSILLW--------------------------------------------------------------------------------------kenvvrkr----KDVVVEHV-----------DIIG--DNFWTGNSQLLS-    190579964       Trichoplax adhaerens                                    eukaryota>metazoa>placozoa  PHYPADRAFT\_217293\_Ppat\_168038223                      MANSKYEYVKDFEVNDN--ILP-HTWIVIRIDGRAFTK-FSQAHQFQKP---NDLQALLLMNASAVAVVED--L-ADVVFAYGVSDEYSFVLRKT----------------STLYQRRASKLISVICSLFASSYVMNWGKYFPETKLQY--------------------------------------APAFDGRAVCYPS--ESILRDYLSWRQ-VDC--HINNQYNTCFWNLV-GSGKSTAESQNMLKGTTADVKNNLLFDTFKINYNDLP-QIFRKGSIVYR---------------------------------------------------------------------------kkvekvvkvedgqeikrlr----SCAVVEHE-----------DIIR--DNFWTQYPYILG-    168038223       Physcomitrella patens subsp. patens                     eukaryota>viridiplantae  CpipJ\_CPIJ000684\_Cqui\_170028733                       MANSRFEYVKSYEHADI--LQK-NCWIVVRIDGKGFHK-FCDVHNFLKP---NDERGLNLMNLAAVHVLQE--F-NEIVLAYGQSDEYSFIFRRD----------------TSVYNRRRDKLISYVGSLFTSAYIFNWRTIFQDTLELKY-------------------------------------PPVFDSRAVLYPT--DQNLRDYMSWRQ-ADV--HINNLYNTSFWNLV-ASGLTNAEAEERLRGTLSSDKNEILFKEFNINYNNVP-VMFRKGTTLMR-----------------------------------------------------------------------------------kkvkltndkkl----SLIVPIFE-----------DMID--DAFWTRHSEILS-    170028733       Culex quinquefasciatus                                  eukaryota>metazoa>hexapoda  PADG\_06749\_Pbra\_226295250                             MANSKYEYVKAFEQDDN--LLP-NTWIVVRIDGRGFHR-FSGRYHFQKP---NDERALNLMNTAACAVMKD--L-PDLIIAYGVSDEYSFVFHRN----------------CQLFERRGSKLVTTIVSTFTAHYIYNWSSFFPSAPLEPGF------------------------------------LPTFDGRAVQYPS--VRNLRDYMSWRQ-ADC--HINNLYNTTFWNMILQGGISNTEAEKELQGTVSGEKNEILFSRFGINYNNEP-EMYRKGSVIFR-------------------------------------------------diahlfetqyetqpqteeekagdgsneveaetgee----dheisc----SPMRFIHQP----------EDVP--SEFWTSATVLEP-    226295250       Paracoccidioides brasiliensis Pb18                      eukaryota>fungi>ascomycota  SPCC63.07\_Spom\_19075481                               MAKSRFEYVKQYERLDR--LLP-ETYIVIRIDGKGFHK-FTKKHDFEKP---NDLRCLNLMNAAARVVMSE--F-TDIVLAYGDSDEYSFVWSKS----------------TELYERRESKLVSHVCSLFTSAFVFNWPKHFDIPLLS---------------------------------------LPSFDGRAVLYPN--MKVLRDYLHWRQ-VDC--HINNLYNTTFWMLILKGGFTNTQAEEYLKGTVSAEKHEILFSKFGINYNFEP-EIYKKGSIWIR-------------------------------------------------------------------------epidqewhqqdkkfsvkqkkk----MVLSILHV-----------SLID--DDFWTSRPFLEV-    19075481        Schizosaccharomyces pombe                               eukaryota>fungi>ascomycota  CD36\_70100\_Cdub\_241957021                             MANSKYEYVKLFEKENY--LLP-DTYIVIRVDGKGFHK-FSQFYQFEKP---NDLKALQVMNSAAEKIMSK--Y-SDVMLAYGDSDEYSFLLRKN----------------CQLYERREMKLTTLFASLMSTYYMYFWQQHFPDKPLDIDH------------------------------------LPNFDARAVLYPD--FKHIRNYFSWRQ-VDC--HINNLYNTTFWNLVLKLKMTPQEAEQRLMGTVASDKNEILFKECGINYNNEL-EMFKKGTIIVR-------------------------------------------------------------------efenyeiedetglskrqaqrlekkrkk----ADLKTYHV-----------DIIND-NSWWDNRPWLRD-    241957021       Candida dubliniensis CD36                               eukaryota>fungi>ascomycota  CAWG\_05412\_Calb\_238883228                             MANSKYEYVKLFEKENY--LLP-DTYIIIRVDGKGFHK-FSQFYEFEKP---NDLKALQVMNSAAEKLMSK--Y-SDVMLAYGDSDEYSFLLRKN----------------CQLYERREMKLTTLFSSLMSTYYMYFWSQYFPDKPLHIDH------------------------------------LPNFDARAVLYPD--FKHIRNYFSWRQ-VDC--HINNLYNTTFWNLVLKLKMTPQQAEQRLMGTVASDKNEILFKECGINYNNES-EMFKKGTIIVR-------------------------------------------------------------------efenyetedeaglskrqvqrlekkrkk----AELKIYHV-----------DIIND-DSWWKSRPWLKD-    238883228       Candida albicans WO-1                                   eukaryota>fungi>ascomycota  THG1\_Calb\_46442219                                    MANSKYEYVKLFEKENY--LLP-DTYIIIRVDGKGFHK-FSQFYEFEKP---NDLKALQVMNSAAEKLMSK--Y-SDVMLAYGDSDEYSFLLRKN----------------CQLYERREMKLTTLFSSLMSTYYMYFWSQYFPDKPLHIDH------------------------------------LPNFDARAVLYPD--FKHIRNYFSWRQ-VDC--HINNLYNTTFWNLVLKLKMTPQQAEQRLMGTVASDKNEILFKECGVNYNNES-EMYKKGTIIVR-------------------------------------------------------------------efenyetedeaelskrqvqrlekkrkk----AELKIYHV-----------DIIND-DSWWKSRPWLKD-    46442219        Candida albicans SC5314                                 eukaryota>fungi>ascomycota  VITISV\_031884\_Vvin\_147819926                          INNIKPEYVKSFQFENK--LMP-LTWIVVRIDGCHFHR-FSEVHEFEKP---NDEQALNLMNSCAVAVLEQ--F-KDVIFSYGVSDEYSFVLKKD----------------SQFYQRRGSEIVTALVSYFTSIYVMKWKEFFPQKELKY--------------------------------------PPCFDGRAVLYPT--SEILRDYLTWRQ-VDC--HINNQYNTCFWMLV-KSGKSKSEAQAYLKGTQAREKNEVLLQQFGIDYNTLP-LMFRQGSSAFW---------------------------------------------------------------------------nevivfsg------kktkq----GESSPPHCLSST-------GYNLC-IQGVSREAFTVLL    147819926       Vitis vinifera                                          eukaryota>viridiplantae  TRIADDRAFT\_55308\_Tadh\_196003010                       IGNRMKDYERQMEWQ----VQA-NQYYIIRLDGHAFTK-YCQ--GLTKP---FDHRIYLALLHTAADLLNK--F--GSRSAFCFSDEISLVFAPTEPSQRSDREGGEEG--IIHYQGRTMKLCSLTAGMASSRFNYYMNMQQFNDTMGALTLARIQSH-----------------------------PAIFDSRLFCLPN--ADEVVKNIYWRA-HDC--LRNSKISFA------QHHVSRQSLH----RVKASEAIKLVKDKKGINYSLDP-DWFRYGTLIKK----------------------------------------------------------------------ilvdhegingktg--epvkvkrs-----RIAYGSINFTSNL------ASNI--EEILLA-------    196003010       Trichoplax adhaerens                                    eukaryota>metazoa>placozoa  GobsU\_010100002157\_Gobs\_168698293                     -----KLYEGAESGRRLMPRLP----ALARLDGRAFHA-FVR--GLARP---FDQRLSDLMIDTLAALVRE----TNATVGYTQSDEFTLAWVPFGAGT------------QVFFDGRIQKMTSALAALCSVHFHRRLPAFLPADYTDR--------------------------------------VPTFDCRVWNVPT--FDEAANVFVWRE-LDA--KKNSISMAA------RAYYDHATVH----GRTGAELQELLFRE-GVNWNSYP-ACFKRGTYSRR------------------------------------------------------------------ravrrpftaderdalp---akhqahrt-----PDLVIERSEVAVFNLPPLSKLENR-AGVLFRGEE----    168698293       Gemmata obscuriglobus UQM 2246                          bacteria>planctomycetes  Mlab\_0759\_Mlab\_124485581                              --------MKDREIFAGLSTTVP---FVLRLDGRSFHR-FSKD-RYKKP---YDKVFSDAMVKTARALVTDSGL--SPSFAYTFSDEISLYVP------------------APVFDCRVEKLASVSAAFAASAFTLYAGASE---------------------------------------------PLAFDARVIPIEE---GLFPAYLSWRQ-AEA--WRNHMNGYAQKILQ-DEGVSPTNAQKQLDGMNAAALHEFAFSR-GVNLALTP-AWERRGICIYR----------------------------------------------------------------------dvvmrdgynpikd--ekvsvertiavi-DEDVPLF------------KPEG--TAWVLSKIR----    124485581       Methanocorpusculum labreanum Z                          archaea>euryarchaeota  CBFG\_01250\_Cbac\_239627528                             IFDELDTGMRRFETSLDQTILP-ELYMAVRIDGRNFTK-LTKETCRFEAP--FDIRFRDAMVDTVKHLMDC-GF--RIVYGYTQSDEISLLFHPR----------------DNTFGRKTRKINSVLAGEASAFFSLRLGV-----------------------------------------------LACFDCRVVPLPN--LECVKDYFSWRQ-EDA--HRNSLNAHCYWLLR-REGMSAREATREIEGKSIAFKNELLFSR-NINFNDLP-NWQKRGVGLYY----------------------------------------------------------------------ssyekkgynpvkd--eevtsirsr-----LDVNMDLEIG--------EAY---TDWVISQIP----    239627528       Clostridiales bacterium 1\_7\_47FAA                       bacteria>firmicutes  METSMIF1\_02117\_Msmi\_261349483                         --------MKEYEVYGDL-KVPVNSKIILRLDGRSFHS-LAKNLNLEKP---YDEDFARLMVKVSKDLFNE--F--APVFIYTFSDEISILLD------------------NIPFNGRIEKINSVVASFASSSFTYNLNKEIAK-------------------------------------------PIAFDSRIIPIID---DDIYKYFKWRQ-DEA--WRNCINAYGIHILK-SK-YGDKVANEKIKGLKSSDIHELLFGE-GINLNDVD-NWKKRGIAIYK----------------------------------------------------------------------qdkeiagynkken--knqvsyrsflfa-DLEIPIFS-----------E------NFFKDINII---    261349483       Methanobrevibacter smithii DSM 2374                     archaea>euryarchaeota  METSMIALI\_01627\_Msmi\_222445979                        --------MKEYEVYGDL-KVPVNSKIILRLDGRSFHS-LAKNLNLEKP---YDEDFARLMVKVSKDLFNE--F--APVFIYTFSDEISILLD------------------NIPFNGRIEKINSVVASFAASSFTYNLNKEIAK-------------------------------------------PIAFDSRIIPIID---DDIYKYFKWRQ-DEA--WRNCINAYGIHILK-SK-YGDKVANEKIKGLKSSDIHELLFGD-GINLNDED-NWKKRGIAIYK----------------------------------------------------------------------qdkeivgynkken--knqvsyrsflfa-DLEIPIFS-----------E------NFFKDINII---    222445979       Methanobrevibacter smithii DSM 2375                     archaea>euryarchaeota  Msm\_0463\_Msmi\_148642523                               --------MKEYEVYGDL-KVPVNSRIILRLDGRSFHS-LAKNLNLEKP---YDEDFAGLMVKVSKDLFNE--F--APVFIYTFSDEISILLD------------------NIPFNGRIEKINSVVASFAASSFTYNLNKEIAK-------------------------------------------PVAFDSRIIPIND---KDIPKYFKWRQ-DEA--WRNCINAYGIHILK-SK-YSDKTANEKIKGLKSSDIHELLFNE-GINLNNVD-NWKKRGIAIYK----------------------------------------------------------------------qnkeivgynkken--knqvsyrsflfe-DFEIPIFS-----------E------NFFKDINII---    148642523       Methanobrevibacter smithii ATCC 35061                   archaea>euryarchaeota  Mhun\_0610\_Mhun\_88601907                               ----MKEREIYADLRTLS-------PIIVRLDGRAFHQ-VTDKLNFSEP---FDEKFSDAMAFVTKGLLIDAGF--APVFGYTFSDEISLYFS------------------ENLFLGRVEKIDSVLASFASSCLTIALELKE---------------------------------------------PVSFDARIIPVTP---DHILPYLSWRQ-QEA--WRNHMNGWSQKLLK-DEGYTSQEAASMLHAMKAAELHEFCFQR-GVNLAMTP-AWQRRGILVYR----------------------------------------------------------------------tvvekegynpktd--ektittrrvvti-DKDIPLFGKP---------D-G---KDFIERIII----    88601907        Methanospirillum hungatei JF-1                          archaea>euryarchaeota  AM1\_3767\_Amar\_158336895                               KFTELDQTLRIYETAYDLCVLPGL-QMVARLDGRNFTR-LTKETLDLDAP--FDATFRDAMVATVQHLMTC-GF--RVLYGYTQSDEISLLLHPD----------------EQTFARKLRKLNSVLAAEASAKMSLILGC-----------------------------------------------IAVFDCRISQLPT--VDLVVDYFRWRQ-EDA--HRNALNAHCYWMLR-KGGDSAGTATQKLEGLSVGDKNELLFQLGQMNFNDLP-PWQKRGVGCLW----------------------------------------------------------------------qsyptagvnpvsg--evsqgtrtkigv-ELNLPMK------------EHY---REFILKRMTL---    158336895       Acaryochloris marina MBIC11017                          bacteria>cyanobacteria  Cpin\_5797\_Cpin\_256424765                              KFDELDSKMRIYETAHDKIVLPGM-YMVARIDGRGFTK-LTKETHPFEAP--FDPRFRDYMVETVKHLMNC-GF--NVVYGYTESDEISLLFHLA----------------EGAFGRKHRKYNSILAGETSAKFSLQLGS-----------------------------------------------LAAFDCRICELPN--RQLVVDYFRWRN-EDA--HRNALNAHCYWQLR-RDNHTRQSATNTIEKMSTADKNELLFRY-NINFNNLP-AWQKRGIGIYW----------------------------------------------------------------------aemekdgvnpktg--eqvvvkrrqlyv-NQELPVK------------DEY---NRFIQELIE----    256424765       Chitinophaga pinensis DSM 2588                          bacteria>bacteroidetes/chlorobi  MC7420\_6362\_Mcht\_254412407                            KFDQLDAKMRVYETAHDHCVLPGL-YMIARLDGRGFTR-LTRDVHPFSAP--FDQRFRDAMILTVEHLMNC-GL--KVIYGYTQSDEISLLVDGA----------------ESAFNRKLRKLNSVLAGEASAKFSLILGT-----------------------------------------------VACFDCRISQLPT--IEIVVDYFRWRQ-EDA--HRNALNSHSYWCLR-NDGKTARQATSQLERLSVADKNELLFQH-GINFNHLP-NWHKRGVGLYW----------------------------------------------------------------------etyqkpglnpltg--etteatrkrirq-ELNLPMK------------DQY---SQFIRDVLS----    254412407       Microcoleus chthonoplastes PCC 7420                     bacteria>cyanobacteria  MM\_1964\_Mmaz\_21228066                                 ----MKNREIYAEMRCIP-------PVVLRADGRNFKN-TLSGLGFEKP---YDTTFARAMADTAELFIKKSGL--SPYFAYTFSDEISFLFT------------------DLPFDGRVEKIDSVVASFLGSALTIKLRLEA---------------------------------------------PIAFDSRLVALQK---EEVSEYFHWRQ-LEA--WRNFVASWGYYSLR-NEGMGKDEAGKFLKGKKEWEIHEMLFER-GINLAALP-AWQRRGIIISK----------------------------------------------------------------------eeceisgfnpvyg--kevksmrrritq-NWEIPKFKS----------EEG---ITFLEKLIN----    21228066        Methanosarcina mazei Go1                                archaea>euryarchaeota  Mmah\_1763\_Mmah\_294496410                              ----MKEREIYADLRCVS-------PVILRVDGRNFQR-ALKKEGFQKP---YDIFFATCMADSIELFFKKSNM--NPVFAYTFSDEASLVFT------------------DLPFDRRVEKLDSVVPSFLSSAFTLFSGIEE---------------------------------------------PVAFDCRVIPVCN---DQFTEYMHWRQ-QEA--WRNFVSSYGYYTLI-DEGIDRKSAASVMHGKKSQDIHEMMFER-GTNLAKKP-AWQRRGVAVYR----------------------------------------------------------------------ekypiegynplle--ektqstrtrisq-DWDLPLFSTA---------EG----DNFLKRHIS----    294496410       Methanohalophilus mahii DSM 5219                        archaea>euryarchaeota  MCP\_2556\_Mpal\_282165226                               ---MADERFKVKEIYKDIKALP---PVIVRADGRNFKE-SLARLKFKKP---YDLKFEKAMVSAGHALMESSGL--GPLWVYTFSDELNVFFS------------------ELPFDGRVEKLDSVVPSFLSSALTLALGVKT---------------------------------------------PLAFDARVVPLHY---EDVSGYLQWRQ-AEA--WRNHMQSYGFYTLV-KDGLAEKYASKTLRGMKFEDIHEMMWQR-GINLNETP-AWQRKGVFIYR----------------------------------------------------------------------kmvmkegydpvkk--kkvrarraevee-SWDPPLFGSK---------EG----EAFLKSLKS----    282165226       Methanocella paludicola SANAE                           archaea>euryarchaeota  HMPREF0198\_1468\_Chom\_258545199                        KFDDLDRLMRPYETAYD-FCVPMGNHIVVRLDGRGFTR-LTKDIWQFDAP--FDPRFRDLMTQTVAHLMQC-GF--NILYGFTQSDEISLLFHPA----------------DDTFARKTRKLASVLAGEASASFTHLHGQ-----------------------------------------------MATFDARVCVLPG--AAQVWDYFHWRQ-EDA--HRNALNAHCYWKLR-QEGASERDAAARISGLKLAEKHDLLHAR-GINYNDLP-AWQKRGIGLYW----------------------------------------------------------------------rdvaqsghnpqtg--eatqttrrrlit-DLELPYK------------EDY---RRFLQGMTAVA--    258545199       Cardiobacterium hominis ATCC 15826                      bacteria>proteobacteria>gammaproteobacteria  Mbar\_A1746\_Mbar\_73669253                              --------MKDREIYAEMRCIP---PVVVRADGRNFKN-TLRDLGFGKP---YDQTFARAMADTAELFIKKSGL--SPLFAYTFSDEVSFLFM------------------ELPFEGRVEKMDSVTASFLGSALTINLQFEK---------------------------------------------PVAFDSRIVVLQK---DEIPAYFHWRQ-LEA--WRNFVAAWGYYTLL-NEGISKIEASKYLRGKKEWEIHEMLFER-GINLAKIP-AWQRRGVIISK----------------------------------------------------------------------eeyeisgfnpvld--ketkslrrriiq-NWEIPNFKS----------EEG---MEFLQKLIN----    73669253        Methanosarcina barkeri str. Fusaro                      archaea>euryarchaeota  Mboo\_2270\_CMet\_154151809                              --------MDNYEIFSNLATIP---PVFVRLDGRAFHG-LTKKYGFAKP---FDDRFCSAMMAACRALVADSGL--APVFAYTFSDEISLYFT------------------GLPFSGRVEKIDSVAASYAASALTLALGAEE---------------------------------------------PLAFDARVVQATP---ETAIEYMTGRQ-DEA--WRNHINSYCQQALI-AEGMDATDAARKLKGLPAAELHEMMHER-GVNLAKTP-AWQRRGILVCK----------------------------------------------------------------------keeekegynpvtd--ehvivtrsrvve-DRDLPLFSTP---------EG----QAFLRMLIC----    154151809       Candidatus Methanoregula boonei 6A8                     archaea>euryarchaeota  Mpal\_0151\_Mpal\_219850835                              -------MNDNREIFSTLAAIP---PLWVRLDGRAFHS-LTADLALERP---FDLRFSEAMATTASALVGSSGL--SPCFAYTFSDEISLYFT------------------GLPFGGRVEKIDSVAASYAASALTLALGVTS---------------------------------------------PLSFDARVVFATP---TSAREYLINRQ-QEA--WRNHINAYCQHALI-SEGLSSREAASRLRGLPGKALHDLMHER-GVNLAETP-AWHRRGLMART----------------------------------------------------------------------advtvsgynpikk--ekvttirhrvvi-DRDLPLFSSP---------EG----EVFLSSVLG----    219850835       Methanosphaerula palustris E1-9c                        archaea>euryarchaeota  M23134\_06676\_Mmar\_124008957                           KFDELDARMRVFESANDFLVLP-NVYMVARLDGRGFTKLTKQNLSLETP---FDVRFKDYMIATTTHLMTC-GF--KFVYGYTESDEISLLFDLE----------------ENIFGRKTRKLVSLLAAEASAKFALLINQ-----------------------------------------------VASFDCRISQLPR--KQDVIDYFRWRN-EDA--FRNSLNAHCYWMLR-KQGISAKKANDEVTGLSVAQKNELLFQA-GINFNDLP-LWQKRGVGFYW----------------------------------------------------------------------ttidkegfnprtn--ekvtaqrktlvt-NDALPMK------------DAY---SNFLTDLL-----    124008957       Microscilla marina ATCC 23134                           bacteria>bacteroidetes/chlorobi  Mthe\_0875\_Mthe\_116754185                              PESSMIRRGRRNEAIY--SELRVRSPFFVRVDGRGFGR-MLRD--FSKP---YDLGFARSIVSAARAFMESSGL--APILAYTFSDEINLLFL-D----------------EP-FRGRLEKLDSITASYISSSLSISLGR-----------------------------------------------VVSMDARVIPICR---EEILSYLQESQ-AEA--WRNHVFSYGFYALL-GEGKSHADAMESLRNMKESDIHEMLFQR-GVNLAKTP-AWERRGVMVYR----------------------------------------------------------------------sg--------------------sgive-EWELPLFTT----------DDG---RIFMEEILS----    116754185       Methanosaeta thermophila PT                             archaea>euryarchaeota  NEIFLAOT\_00025\_Nfla\_225075026                         RFDDLDKRLRQYETAYD-FCVPQENFIVVRLDGRGFTR-LTKEIWQFEAP--FDIRFRDLMAHTVQHLMKC-GF--NVAYGYSESDEISLLLRRD----------------DDTFKRKTRKIISVLAGEASAAFSVAHGQ-----------------------------------------------PAAFDARVCVLPN--EKLVVDYFRWRH-EDA--HRNALNAHCYWMLR-KKGESVSRATEAVSGLTRAQKHDLLFEH-NINFNELP-AWQKRGFSVYF----------------------------------------------------------------------qttlkegfnpqtg--etaqaerqilht-DFELPLG------------DDY---GAFVLERIG----    225075026       Neisseria flavescens NRL30031/H210                      bacteria>proteobacteria>betaproteobacteria  NEIELOOT\_02904\_Nelo\_294671196                         RFDDLDKRLRQYETAYD-FCVPQENFIVVRLDGRGFTR-LTKEIWQFEAP--FDIRFRDLMAHTVRHLMQC-GF--NVAYGYSESDEISLLLRRD----------------DDTFKRKTRKIISVLAGEASAAFSVAHGQ-----------------------------------------------PAAFDARVCVLPN--EKLVVDYFRWRH-EDA--HRNALNAHCYWMLR-KKGESVSRATDAVSGLTRAQKHDLLFEN-GINFNELP-AWQKRGFGVYF----------------------------------------------------------------------qttlkegfnpqtg--etvqverpilqt-DFELPLG------------DDY---GAFVLERIG----    294671196       Neisseria elongata subsp. glycolytica ATCC 29315        bacteria>proteobacteria>betaproteobacteria  NEISICOT\_02755\_Nsic\_255067726                         RFDDLDKRLRQYETAYD-FCVPQENFIVVRLDGRGFTR-LTKEIWQFEAP--FDIRFRDLMAHTVRHLMQC-GF--NVAYGYSESDEISLLLRRD----------------DDTFKRKTRKIISVLAGEASAAFSVAHGQ-----------------------------------------------SAAFDARVCVLPN--EKLVVDYFRWRH-EDA--HRNALNAHCYWMLR-KKGESVSRATDAVSGLTRAQKHDLLFEN-NINFNELP-AWQKRGFGVYF----------------------------------------------------------------------qttlkegfnpetg--etvqaerqilht-DFELPLG------------DDY---GAFVLERMV----    255067726       Neisseria sicca ATCC 29256                              bacteria>proteobacteria>betaproteobacteria  PlimDRAFT\_36970\_Plim\_229539832                        ----LDNKMRVFETAADLCVLP-GMFMVARLDGRSFTRLTKDVCPFEAP---FDERFRDLMVSTTESLMNC-GF--RVLYAYTQSDEISLLFDLE----------------EQLFGRKLRKYNSLLAGEASAQFSLKLRQ-----------------------------------------------PACFDCRISQLPT--SELVVDYFRWRN-EDA--ARNALSAWCYWTLR-KDGQNEQQATKRLLGLSVSQKNQLLFQS-GINFNDLP-NWQKRGVGFYW----------------------------------------------------------------------eeydrpamnpitk--ekvtarrrrlht-DFDLPMQ------------DDY---SQFVRLIVS----    229539832       Planctomyces limnophilus DSM 3776                       bacteria>planctomycetes  sce6062\_Scel\_162454339                                ----MDAKMRLGECFHELRALR-GAYIVIRVDGRSFSR--LTERTCEKP---FDTGFHHKMTDAAKAVLTS--L--HGSYAHTQSDEISILLPHE----------------TDVFDREVEKLVSIAASSATASLSLALSA-----------------------------------------------PVEFDARLWLGAR--RGDVVDYFRWRQ-ADA--ARSALNNWCYWTLR-KEGKSVKEATHAMLGLSVAGKNELLFSR-GINFNDVP-AWQRRGSGVYW----------------------------------------------------------------------etiakvgrnprtg--esvpatrrrlrv-DDDLPMK------------EAY---GKFVRQFLK----    162454339       Sorangium cellulosum 'So ce 56'                         bacteria>proteobacteria>deltaproteobacteria  DDB\_G0289191\_Ddis\_66805193                            GNFRNYNKFELFEKAFDFVLPP-YQPIIIRLDGNSFSK-LNKQLKLER----HDERFHESMKETSNNLFSH--F-IGCKFIYSFSDEINVIYNQYPD--------------NQFLSNRIQKLISTTSSITSLNFSINLSKKLNNNNNNNNV------------------------------------FSYFDCRAFVLPI---NEVKDYFIKRQ-GRC--YTNFLSSIASDNGF-NTDSIFNNDSGLDGSKKNIYAREYYLEKQGVNLMEIP-EHFKSGFLVFK-----------------------------------------------------------------------nst-----------nel----------DYKSPI-------------NFKS--DTIIDYLLN----    66805193        Dictyostelium discoideum AX4                            eukaryota>amoebozoa>mycetozoa>dictyosteliida  Mbur\_1464\_Mbur\_91773429                               --------MKKREVYSDLRCIP---PVIVRLDGRTFKH-TLSRLGCEKP---YDEKFASAMADSLELFFKKSGI--NAALAYTFSDEASILFF------------------DLPFDGRVEKLDSVISSYLSSAFTIKMGLDE---------------------------------------------PVSFDSRIVPVNK---ENVAEYLIWRQ-SEA--WRNCVSSYGYYTLL-SEGMSKKDAASAIKGKKAQGIHELLFQR-GINLDKVP-MWQRRGILVYK----------------------------------------------------------------------ddykvagfdpvrk--eirsstrnkvvq-DWEVPQFSS----------EEG---TDILKKHLI----    91773429        Methanococcoides burtonii DSM 6242                      archaea>euryarchaeota  CMU\_028750\_Cmur\_209557756                             MACSKYEYVKSYEQS--NRVLN-NSWFVVRIDGCSFHE-FTRSHNYDKP---NDKNGLDLMNRAAESVMRK--I-SDIIIAYGQSDEYSFVFRRK----------------TDLWGRRYEKILTYTVSLFTSSFVYYWDNFFPKIRLTY--------------------------------------PPTFDGRIIIYPT--DKDIRDYLSWRQ-VDC--HINNLYNTCFWALVKFRNISEKEATELLKHSVSSDKNELLFSEFNINYSKIP-KQFRKGTVLYR--------------------------------------------------------------------prqktkktsseyfn------mrdird----KEVMIDDTKDSSDE-----DIT---HPIWSCK------    209557756       Cryptosporidium muris RN66                              eukaryota>alveolata>apicomplexa  C4\_0009\_Uarc\_82617278                                 ---MRVSDFKDHEIYADLRATP-P--VVIRVDGRSFKN-LLRKHRFEKP---FDHRFASAMADATESFFQQSGL--YPVVAYTFSDEINILFR-D----------------ALPFDGRIEKLVSVVPSSISSALTRSLKIS----------------------------------------------PIAFDGRVIPLHP---EQIIEYLVWRQ-AEA--WRNCINGYGYYTLR-SNGLSGKDAASRMRGLRASDIHELCFQH-GINLGKVP-LWQRRGVLVYW----------------------------------------------------------------------eeytkpgydpvrd--ievvvnrkrvaq-NWDLPIFAS----------DEG---GVMVRGLVG----    82617278        uncultured archaeon                                     archaea  BSM\_02380\_Uarc\_268323173                              NQARHKYKAQDREIYANMKAIT---PLFVRADGRNFKR-VLST--FGKP---YDARLAKGIVKAVELLFLNSGF--NPKLAYIFSDEINLYFD------------------DVPFKGRIEKLDSVLASFLASALTIILDFKD---------------------------------------------AIAFDARVIPVCG--EADVLEYLAQRQ-AEA--WRNHINAYGYYGLQ-DTGLSEKEAEKRLKGMKAAEVHEMLFRL-GINLNETP-KWQRRGILIAR----------------------------------------------------------------------qrhekegydpkla--ekvtatrykvvs-LWDPPLFGS----------EEG---RNLIHQLLD----    268323173       uncultured archaeon                                     archaea  MTH972\_Mthe\_15678990                                  ---MAGHSMREHEVYSKLRAPP-SSRMILRLDGRGFHR-LTESLDFDRP---YDEAFRDLMIRTCLDLMEE--F--SPSLIYTFSDEINVLLD------------------SVPFAGRVEKLDSVFSGFASSSFTRGALEAGFSPVK----------------------------------------PVSFDCRVIPISS---DIVPEYFRSRQ-DES--WRNCLNSYAYWTLR-RE-VGARRAAERLRGLKSDSLHDLLFER-GVNISRVP-AWQRRGVGVYR----------------------------------------------------------------------vphrvrgynpird--eevsavrmrvkv-DLELPLFTD----------EFF---EGLMR--------    15678990        Methanothermobacter thermautotrophicus str. Delta H     archaea>euryarchaeota  MXAN\_5968\_Mxan\_108762077                              -----AARARQGEVFHGQRMLP-GAWVVLRVDGRGFSR-FTEA-RYEKP---FDPVFHQFMVRTASVMLEE--L--QGVYAYTQSDEISVLFRPD----------------WALFDRSVEKVVSLAAGLASATFTHAAGV-----------------------------------------------PAVFDGRAWLGAS--ERAVLDYFIWRQ-ADG--SRCSLHGWCYWTLR-KEGRSAAQATRELDGKPVSYKNELLFQR-GINFNDVP-LWQRRGSGVWW----------------------------------------------------------------------eayqkegvdprdg--rrtqtlrrrlkv-DSELPMK------------EAY---EHLVRGLLA----    108762077       Myxococcus xanthus DK 1622                              bacteria>proteobacteria>deltaproteobacteria  Haur\_4049\_Haur\_159900562                              NFDQLDQLMRVYETSNDLAVLP-NIWMVVRIDGRSFTR-LTKDVMNYQRP--FDQRFRDAMISTVKHLMEC-GF--PVLYGYTQSDEISLLLSYG----------------CDVFGRKLRKYCSILAGESSGHFSLAIQQ-----------------------------------------------QASFDCRICQLPT--DQLVHDYFRWRM-EDA--HRNALNAYCYWSLR-DQALDPQTAHMQIANLSVAQKNELLFQH-GINFNDLV-AWQKRGIGFYW----------------------------------------------------------------------qtvdlqgynpqtq--mptltkrrkihv-EFELPQR------------EAY---SNLLASHFD----    159900562       Herpetosiphon aurantiacus ATCC 23779                    bacteria>chloroflexi  BGP\_4908\_Bsp.\_153872919                               ----FDARMRIFETTHDCCVLP-CLYMVARLDGRNFTR-LTKEVCQFEVP--FDFKFRDIMIETVKHLMNC-GF--RINYGYTQSDEISLLFHL----------------NEDTFNRKLRKFNSVLAAEASAKFSLLLGK-----------------------------------------------MGCFDCRISQLPT--KELVSDYFRWRQ-ADA--QRNALNAHCYWLLR-KEGQSQQDATTALLNLSVSDKNEFLFSH-GVNFNELP-HWQKEVLVYIGklmknkp---------------------------------------------------------------------------------------------------------------------------------    153872919       Beggiatoa sp. PS                                        bacteria>proteobacteria>gammaproteobacteria  thg1\_Ppal\_281212173                                   MANSKYEYVKLFEQP--DSLLP-NVWIVVRVDGRGFHK-FTQKHDYAKP---NDDRGLNLMNRCALEVCKE--F-TDIVIAFGESDEYSFVLKKQ----------------CTLFERRESKITSSIVSYFSSQFVYRWKEYFGDFELKY--------------------------------------PPTFDSRAVCYPS--DQNLRDYLSWRQ-ADT--HINNLYNTAYWALVLKGGKTPNEAELELRGTLSEQKNEILFSRFAINYNNLP-QMYRKGSVIYR--------------------------------------------------------------------kmvaeeridsrtgq------pttkqkk---RPITKRTKNNDFGGKNRKKKGD-VDDDVWLWSTIAG--    281212173       Polysphondylium pallidum PN500                          eukaryota>amoebozoa>mycetozoa>dictyosteliida  MK0093\_Mkan\_20093533                                  ---MKPVEPCDLEVYADLRVPP-NTHLVLRIDGRAFTK-LTRRLGLKKP---YDRRFAEAMAETAVRMIRDAGL--GITLVYTFSDELNALIPRG----------------NVPFSGRVEKLTSVSASCASTYFFRALQRHGIDPTGE---------------------------------------TVSFDSRCVVLTD---DDLVDYFKWRQ-DEA--WRNHLNSYAYWALR-ERGLKPKEAAERLRGMKAHDVHELLYREFGINLGRTP-AWQRRGILAYR----------------------------------------------------------------------vavnedgvq------------rrrvtr-DWAPPFFD-----------ESE--GERLIRACASQGY-    20093533        Methanopyrus kandleri AV19                              archaea>euryarchaeota  Msp\_1147\_Msta\_84489939                                ---------KEYEQFSQLKIVP-DLPIIIRIDGRSFSK-YTKQLGLKKP---FDKRLRNLFIEVSKDVLGE--F--NTKYIYTFSDEINILLE------------------QIPFNGRVEKIDSVICSYVSSSFMKHLFISKDQFDVDITTLK----------------------------------PASFDSRII-ITS---KNIKNYFKWRQ-DES--WRNCLNSYAQYVLN-KE-NKPEKTAKILYKLNKSNIHEL-LFNHGINIAHVP-TWQKRGVAIYK-----------------------------------------------------------------------------------ikeekegynpklk---KKTLYFR-----------N-----KVYVDKNISLFK-    84489939        Methanosphaera stadtmanae DSM 3091                      archaea>euryarchaeota  Memar\_0402\_Mmar\_126178352                             ------MDTKDREIFSTLTIFP---PVFVRLDGRAFHR-LARARNLKKP---FDPAFNESMRAVCRYLLTGSGL--SPAFAYTFSDEISLYFN------------------ALPFSGRVEKLDSVTAAVAASKLTIELGCTE---------------------------------------------PLAFDARTIPAAG---EFAVEYLVSRQ-NEA--WRNHINAYCQNALV-EEGMTAREAAAMLRGMQSEAMHEMMFER-GVNLAATP-AWQRRGTLLYR----------------------------------------------------------------------decikegynpltg--etveavrtcire-PEETPLFSTP---------E----GEALIRSLTG----    126178352       Methanoculleus marisnigri JR1                           archaea>euryarchaeota  Kcr\_1204\_CKor\_170290817                               NASIRSVDWKEREIFSGLR-VPSDAPVMVRIDGWRFHK-VADELGLERP---FDRRLIEALIQAPLSLMRM-GF--PLALSFAFSDEISFLIY-----------------PPIPWSGRVEKLISVIPSHSSAIVSMFLNY-----------------------------------------------PVCFDARIIILRD--LDEILGYLSWRQ-SEA--WRNALNSYALFALE-RSGMNREDAVKELRNRKADSLHDIIFTKLGINIATVP-SWQRRGVIVRK----------------------------------------------------------------------------ryeekhc--esgkvvrrvpev-DWDIPLFSTP---------E----GRDYLLEALR----    170290817       Candidatus Korarchaeum cryptofilum OPF8                 archaea>korarchaeota  thgL\_Mrum\_288559933                                   --------MKDYEIYSNL-KVPKNSNIILRLDGRKFHS-LAKYLALKKP---YDNNFAKLMSEVSLDIFNQ--F--SPKFIYTFSDEISILLD------------------EIPFLGRVEKLNSVFSSIASSSFTYHLLNDFKDDFNMDKLDEDDRNVIF---------------------------PVSFDSRVIPIVN---EDIYDYFKWRQ-DEA--WRNCVNAYGIWALK-KE-FSPQIANEKIKGLKSSEIHELLFKK-GINLNDVD-TWKKRGIGIYK----------------------------------------------------------------------ienqiegfnpvke--ektvsyrsevfv-DYELELF------------N----KEYFYRF-------    288559933       Methanobrevibacter ruminantium M1                       archaea>euryarchaeota  RCIX455\_Umet\_147920979                                --------MKRKEIFSAIRAAP---PVIVRLDGRNFKE-SLSRLGFAKP---YDLRFQQGMVAAARMLVEQSSL--APEWAFTFSDEVSLLFK------------------KLPFDGRLEKLDSVIPSYMASALTIALKVET---------------------------------------------PLAFDSRVIPVHP---EEIPEYLAGRQ-AET--WRNHMQSYGFYTLV-SEGMGEKEAAAKMKGMKFEDIHEL-MWQRGVNLNETP-GWQRKGVFIYR--------------------------------------------------------------------kk--ttregynplkg--ekvsverrev-v-EDWDPSLFTS---------DE---GKAYLKQALG----    147920979       uncultured methanogenic archaeon RC-I                   archaea>euryarchaeota  Arcpr\_1732\_Apro\_284162827                             RQKRKRINWKDRELYAHLLAPN---VFILRIDGRNFTN-VLKD--FEKP---YDIRFARAMVETCREIMRE--F--NPAFAYTFSDEVSFLFR------------------D-LFGCRVEKIDSIIASEFSSRLSLKLGF-----------------------------------------------PVSFDSRIIYASF---DEISDYLKSRQ-DEC--WRNHINSYAFYTLL-KEIKDRRKTQEFLSGKKSSEIHDLLFER-GINISKTP-AWQRRGIMLYW----------------------------------------------------------------------eevefe--kefeg--rkvrfkrrrive-EWNLPLFDS----------ED---GKKLIEKVLS----    284162827       Archaeoglobus profundus DSM 5631                        archaea>euryarchaeota  CTRG\_05039\_Ctro\_255731634                             MANSKYEYVKLFEKE--NYLLP-DTYIIIRVDGKGFHK-FSQFYQFEKP---NDLKALQVMNKAAEKIMQK--Y-SDIMLAYGDSDEYSFLLRKN----------------CDLYERREMKLTTLFASLMSTFYMHFWQQIFPDKPLVIDH------------------------------------LPTFDARAVLYPS--FKHIRDYFSWRQ-VDC--HINNLYNTSFWSLVLKLNMTPQEAEQKLMGTVSSDKNEILFKECGINYNNEL-EIFKKGTIFVR-------------------------------------------------------------------efenyevqietglssrqkqrlekkrkk----AQVKEYHV-----------DIIN--NSWWETRPWLEE-    255731634       Candida tropicalis MYA-3404                             eukaryota>fungi>ascomycota  LELG\_05489\_Lelo\_149234768                             MANSKYEYVKAFERE--NYLLP-ETYIVIRVDGKGFHK-FSKYYDFAKP---NDLGALQVMNAAAMQIMHR--Y-SDVLLAYGDSDEYSFLLRRS----------------CELYERREMKLCTLFASLMSTYYMHFWNQRYPEKLIQLEM------------------------------------IPTFDARAVTYPN--FRTVRDYFSWRQ-VDC--HINNLYNTTFWNLVEKLGLTGQEAENKLMGTVSSDKNEILFKECGINYNNEP-EIFRKGTIFVR---------------------------------------------------------------eivnskecndddeikqlsarqkqrqekmrkk----AEIKEYHV-----------DIIN-DDNWWESRSWLER-    149234768       Lodderomyces elongisporus NRRL YB-4239                  eukaryota>fungi>ascomycota  At2g32330\_Atha\_3831463                                KDIGKIEPDYVKSFQFESRLLP-LTWVVVRIDGCHFHR-FSEVHEFEKP---NDEQALKLMNSCAVAVLEE--F-QDIAFAYGVSDEFSFVLKNK----------------SELYKRQSSKIISAVVSFFTSTYMMRWGDFFPHKKLKY--------------------------------------PPSFDGRAVCYPT--SDILLDYLAWRQ-VDC--HINNQYNTCFWMLV-KSGKSKIQAQDYLKGTQTREKNELLSQQFGIEYNSLP-VIFRMGSSVFR----------------------------------------------------------------------lkv--------------------------LLLLSYS-----------K-----ILLVFHSRFLLL-    3831463         Arabidopsis thaliana                                    eukaryota>viridiplantae  RCOM\_0075620\_Rcom\_255578805                           IGKVNPDYIRSFLFK--SKLLQ-STWVVIRIDGCHFHR-FSDGHEFEKP---NDEQALNLMNSCAVAVLKE--F-QDVVFAYGVSDEYSFVLKKD----------------SKFYSRQASDIVSVIVSFFSSMYVMNWKSFFPQKDLKY--------------------------------------PPSFDGRAICYPS--SEILQDYLAWRQ-VDC--HINNQYNTCFWALV-KSGKSKTDAQSTLKGTQVREKNE-ILAQFGIDYNNLP-LIFRQGSSVFR--------------------------------------------------------------------------vkedivihe--ngasaknlr----TKFL---------------EGAP-RHPWLEAPATRTN-    255578805       Ricinus communis                                        eukaryota>viridiplantae  Pars\_0105\_Pars\_145590370                              ------MRYREREVV----CEPASPPFAVRLDGVGFGS---RLRDFPHP---RSRLVHNALVEVARTIAQT--Y--GADFAHVVSDEINLFFHR-----------------LAPYGGRTFKIISVLAGHASAEATALLGR-----------------------------------------------PLYFDGRVVKLRD--LCDAATYFLFRA--RV--GLNNYAVQIA-----RGMGLLRERTPPIGEVVAKIKIDDYELAWGAFLEKER-G-YRKEVDVCN-------------------------------------------------------------------------------------------------aLSSLCNVC------------------------------    145590370       Pyrobaculum arsenaticum DSM 13514                       archaea>crenarchaeota  Tneu\_0097\_Tneu\_171184582                              NPRHLEMRYREREAV----CEPASPPVAVRLDGVGFGK---RLRDFPAP---RSRLVHTAIAEVARDLAAQ--H--GAEFVHVVSDEINLLFL-----------------TSVPYGGRTFKLASVLAAQAAAGVTAKLGR-----------------------------------------------PLYFDGRVVKLAD--RCDAARYVLFRA--RV--GLNNYVVKAA-----RLAGVVGAETPRIEELLAAVKIEDFELAWGSFMYKEG-G-YARSGDLCN-------------------------------------------------------------------------------------------------aVSQLCEMC------------------------------    171184582       Thermoproteus neutrophilus V24Sta                       archaea>crenarchaeota  PAE0886\_Pyae\_18312250                                 NPRLLEMRFREREAV----CEPATVPFAVRLDGVGFGK---RLKDFPPP---RSRLVHNALVEVAKSLALT--Q--GADYVHVVSDEINLLFFR-----------------AAPYGGRTFKIISVLASQASAELTAKLGR-----------------------------------------------PLYFDGRVIKLRD--NCDAASYVLFRA--RV--GLNNYVIQLA-----RGAGLIREYTPPIEDMLKSVVIEDYELAWGTFMRRED-G-FKKGVDMCS-------------------------------------------------------------------------------------------------aLSRLCNVC------------------------------    18312250        Pyrobaculum aerophilum str. IM2                         archaea>crenarchaeota  Pcal\_0070\_Pcal\_126458698                              NPKALEASYREREAV----CEPAAPPFAVRLDGVGFGK---RLRDFPHP---RSRVVHEALVDTAKTLAAT--Y--GAELVHVVSDEINLIFL-----------------GQAPYGGRTFKIVSVLAAHAASELTARLAR-----------------------------------------------PLHFDGRVIRLRD--RCDAATYVLYRA--RV--GLNNYVVQLA-----RGAGLINSHTPHIEELLPKVEIADFELAWGSTMKKEG-G-YRREG-LCE-------------------------------------------------------------------------------------------------aLVALCDVCTSGT--------------------------    126458698       Pyrobaculum calidifontis JCM 11548                      archaea>crenarchaeota  Pisl\_1144\_Pisl\_119872651                              NPRLLEMRYREREAV----CEPSSPPIAVRLDGVGFGK---RLKDFPAP---RSKLVHAALVEVAKNLAMQ--H--GASFVHVVSDEINLVFL-----------------NVVPYGGRTFKIISVLAAQAAAELTAKLGR-----------------------------------------------PLYFDGRVIKLDS--SCDAAKYILYRA--RV--GLNNYVVQLA-----RATGLIKTQTPHIEELLPKVEIGDFELAWGSFMSKED-G-YLKNSDLCR-------------------------------------------------------------------------------------------------aVSTLCEIC------------------------------    119872651       Pyrobaculum islandicum DSM 4184                         archaea>crenarchaeota  Achl\_4028\_Achl\_219882632                              --DQTTTDMKNREKQYRTFL-PAKSYAVIRVDGKGFSK-YTR--GLQRP---FDPKFTADMQATALYLCEN--I-DGAQFAYTQSDEISVIISDLGSANT-----------QAWFGGQVQKIVSTSAALATAKFNRIRPEIDA--------------------------------------------LAFFDGRTHHLEG--SAGVLEYLQWRQ-ADA--MKNSVGMLA------SHHFSHRELT----GVSVRRRKEMLAGR-GILWEDLG-QEVKQGTFVRR----------------------------------------------------------------------------------------------vlt-ERSISYL------------HK---KEQVMKTSD-----    219882632       Arthrobacter chlorophenolicus A6                        bacteria>actinobacteria  CORMATOL\_02529\_Cmat\_225022489                         --MNLDTRMKKYEYVTRTYLTCRMP-VIVRLDGKAFHT-FTR--GLKKP---FDSVFNSAMDDTMLYLAQN--S-QNCMLAYRQSDEISLLLVDYATFE-----------TAAWFDNNISKIVSITASMATAVFNESFKKHALAQLESETDQKYCNALRRCIDN-----------------------LALFDSRAFNIPR--E-EVANCFWWRQ-KDA--IKNSIASLG------QAHFSPRELH----GKHGQQIQEM-LETKGISWEDAP-TPQKRGACALR-----------------------------------------------------------------------------------------ndegqwyv-DHNIPVFT-----------E----DWNYIDRFVD----    225022489       Corynebacterium matruchotii ATCC 33806                  bacteria>actinobacteria  CORMA0001\_0334\_Cmat\_252128517                         --MNLDTRMKKYEYVTRTYLTCRMP-VIVRLDGKAFHT-FTR--GLKKP---FDPVFNSAMDDTMLYLAQN--S-QNCMLAYRQSDEISLLLVDYATFE-----------TAAWFDNNISKIVSITASMATAVFNESFKKHALAQLETETDQKYCNALRRCIDN-----------------------LALFDSRAFNIPR--E-EVANCFWWRQ-KDA--IKNSIASLG------QAHFSPRELH----GKHGQQIQEM-LETKGISWEDAP-TPQKRGACALR-----------------------------------------------------------------------------------------ddegqwhv-DHNIPVFT-----------E----DWNYIDRFVD----    252128517       Corynebacterium matruchotii ATCC 14266                  bacteria>actinobacteria  DDB\_G0279743\_Ddis\_66814690                            FSALGDRMKSYEDGMKITIEKNN--SFIIRLDGHSFSK-FSK--VFKKPGIAWDIRIHQAMVETATALMKT--F--LPTVVYTFSDEITMCFPSIDKEAIDDG-EIP----QLAYNGKVQKLISLTAGLASTVFYKSITQALYDTDKEEKIIDLLKTA-----------------------------TPSFDSRLFVLPS--NDEIRHNLIWRSIIDC--KRNSVSQVG------QSHFLPKQIH----GLSGQEIKKKLLLEKGIDFNDEP-DWYKYGVYLKK----------------------------------------------------------------------qnythkgfspikpt-qeitvlr------NKVVPFSF-----------DIT--KLSNSNDFITK---    66814690        Dictyostelium discoideum AX4                            eukaryota>amoebozoa>mycetozoa>dictyosteliida  ERE\_34100\_Erec\_291529569                              DLETRMKTYENVPKNKLMRRCP----VAIRLDGCHFKS-FTK--GFDKP---FDNVFMKSMQETMKYLCEN--V-QGCVMGYTQSDEITLILVDYEHLN-----------SEAWFDDEVEKICSVTAGMASMAFYRIFCKNAHGFLSENGYPHVHMLKNEKLINAYRRAWRQ---------------GAYFDARCFNIPK--E-EVTNLIYWRQ-CCG--HRNAIQAAG------CTYFSNKELL----NKSGDEIIKM-LKEKGIEWSAYS-NDAVWGSCCVR--------------------------------------------------------------------nnrpvimpdgrrtyy--mydknkrtkwii-DYHIPVFTG----------A----GRLYINALVY----    291529569       Eubacterium rectale M104/1                              bacteria>firmicutes  nfa39460\_Nfar\_54025916                                --MNLGDRIKAYEAASNYRLTP-NSCVFLRVDGKAFHT--FTR-GMQRP---FDPALMQTMVDAAVETARE--M-QGFKLGYVQSDEATFLLTDFDTHD-----------TAGWFGYEVNKLVSISASTMTMHFNRLFREKP---------------------------------------------MAVFDSRAFVVPR--H-DAPNAFVWRQ-QDW--ARNSLQMLA------RAHFSHRELH----GKGRAELHEM-LMERGVNWAALS-AREKNGTFVLA--------------------------------------------------------------------------------------dksvisekw---GYE----------------DID----VYLTGLMP----    54025916        Nocardia farcinica IFM 10152                            bacteria>actinobacteria  RBTH\_06728\_Bthu\_75758411                              -MDSIGDRMKRYENAYR-IKLPERMPVIVRIDGAHFHT-YTK--GCAKP---FDQDLAEAFWETCKYLAQN--I-MGAKLVYHQSDEISILITNYDKLT-----------TQSWFENNLQKIASVSASMATAKFNEVMREKYPDKP-----------------------------------------LATFDGRAQVLPQ--D-EVANYFIWRQ-QDA--SKNSISMVA------QANFPHKQLQ----GLNGKDMQDKLMTEKNINWNDLP-VWQKRGICIIK--------------------------------------------------------------------------------efy--ekngalrsrwsv-DHETPIISK----------D-----REYVEQFVY----    75758411        Bacillus thuringiensis serovar israelensis ATCC 35646   bacteria>firmicutes  DDB\_G0275625\_Ddis\_66819813                            ELLGDRMKSYEDDMKIQ--IEK-NKPFIIRLDGHSFSK-FTK--NFNKP---HDIRIHNAMIETSTVLLKT--F--MPTCIYTFSDEITMCFPSIDESTLEEGKEIP----NLAYSGKVQKLISLSSGLASTVFFKSITNAQYDKDTELNLIKLLETC-----------------------------TPHFDARIFTLPS--NQEIVNNLIWRSLVDC--KRNSVANLG------FAHFTPKQML----GLNNTEVKKKLLEEKSIIYENEP-AWYRFGTYLKK----------------------------------------------------------------------eyytlttvspnepe-kqitair------SKVNCFSF-----------DITK-LSNSLNFISF----    66819813        Dictyostelium discoideum AX4                            eukaryota>amoebozoa>mycetozoa>dictyosteliida  Sterm\_2087\_Ster\_269120695                             VHDDFGKRMKTYENSYR-FTLPRRMPVILRIDGCHFHT-FTK--GMDKP---FDDKLIEAFWETCKFLGEN--I-MGAKLIYHQSDEISILITNYDTIQ-----------TDSWFSNNLQKMASVSASMAAAKFNEVIRKSYSDKE-----------------------------------------LAFFDSRAWVIPQ--D-EVNNYFTWRQ-QDA--SKNSISMAA------FANFAHKDLH----GLSGNQLQEKLFSEKGINWDKFP-TWKKRGACIIK--------------------------------------------------------------------------------key--lkenairrrwet-DLDIPLFSK----------D-----RNYVERFVY----    269120695       Sebaldella termitidis ATCC 33386                        bacteria>fusobacteria  PPSIR1\_23574\_Ppac\_149920560                           ---ALGDRMKLYERAEAGRRFMPLLPICARIDGKRFSR-WTK--GLARP---YDERLSATMVAVTQALVED----THARVGYTQSDEISLVFYSESADS------------QVFLDGRVQKLTSILASMATARFNTEARARVPERADA---------------------------------------PALFDCRCWTVPN--LDEAANALLWRE-RDA--TKNSLSMAA------RSHYSHAQLD----GKRGPDMHEMLHAA-GVNWNDYP-SFFKRGTFVRR--------------------------------------------------------------ervsrpfsteelerlpprhaaranpdlviertvvr-AVDMPPLNRI---------RNR--VAALFEGAE-----    149920560       Plesiocystis pacifica SIR-1                             bacteria>proteobacteria>deltaproteobacteria  201phi2-1p319\_BP201phi2-1\_189490470                   --DQLGDRMKMFENDFIVKRFMPGLPIVARIDGRGFSR-FTR--GMKRP---YDPDMSAAMIHTTRELVKH----TQATVGYTQSDEITLIWYSNDYKS------------MNWFDGRVQKMVSLLGSHATLYFNQYIMQYMPQYAKR---------------------------------------NPTFDARVWNVPS--LEEAANVLVWRE-WDA--TKNSIQMAG------HHYFSNKELH----KKNTSEIQEMLWSQHDVNWHHYP-VFFKRGTYIGW-----------------------------------------------------------kqfragrrltdeemlllppkhhahkndgwieketrvlt-ELSLPPIT-----------QIEN-RVEFFFGAKT----    189490470       Pseudomonas phage 201phi2-1                             dsDNA viruses, no RNA stage>caudovirales  Igni\_0485\_Ihos\_156937279                              ---------MRVEG-------P----VIVRMDGVSFGK-YSK--LLGKH---RDERLHNALVSSAKELVEY--Y--SCDSAYVSSDEVSVYCK------------------LPPFGGRVEKLVSVFSSFLGSHFSVKVSPLP---------------------------------------------PGWFDGRVVLAGD-----WKAYVMWRL--KV--TVCNY----------ASSVARKPCSQALKEVRLPPEA------FGTLLERVE-VLKKGYTR-------------------------------------------------------------------------------------------------------------------------------------------    156937279       Ignicoccus hospitalis KIN4/I                            archaea>crenarchaeota  MIMI\_R259\_APMV\_55819137                               INEPIGDRMKRFEAKYDFKIEP-QNYFCVRLDGNKFSN-FTR--KFEKP---YDVRFSQAMVMTTIDTINK--F--GARTGFTQSDEITLIFDKAIPDDFKKHITY-----NHLFNGRVSKLLSIVSSYVSVRFNHHFRLLTSNLTNIYSQETLELINGG---------------------------TAIFDARILEFDENNKYEMLNHLIWRSVKDC--YRNAVQTYA------HHIFGPAKIK----YLNREQMIQLIEENTNIVWSNIP-LWQKYGVIIKK----------------------------------------------------------------------qliktdnvkn--------svit------SSFKVFSL-----------KLSY-NDTMLKFLFD----    55819137        Acanthamoeba polyphaga mimivirus                        dsDNA viruses, no RNA stage  bthur0002\_61310\_Bthu\_228982997                        SSNKLGARMKEYENKTKLTKRSP---VIIRIDGTHFHT-YTK--NMKKP---FDEVLAKAFWETAKYLAQN--I-MGCKMVYHQSDEISLLLTNYEKVT-----------TQSWFGNDLQKMVSVSASMATAKFNEIMFPITGT-------------------------------------------LAFFDSRAFVLPK--E-EVTNYFLWRQ-QDA--TKNSIAMVA------QANFKHKELQ----GYNGSQLQEKLFTEKNINWNNLP-IWQKRGVCITK--------------------------------------------------------------------------------sqy--lkgtatrtkwdv-DFNTPQFS-----------KD----RDYIDQHVF----    228982997       Bacillus thuringiensis Bt407                            bacteria>firmicutes  NECHADRAFT\_87153\_Nhae\_256722828                       ----LASRMKEYEAITEIHLDP-SKPAILRLDGHSFSK-FTA--SFAKP---FDERLHTAMVKTCADLLGA--Y-SSASLAYTQSDEITLVFPDGV---------------GSQFNGRVAKIASLAAGRCSVHFYSHLVAAVLETPEPPVRGFSSVPFPHFDQQR----------------------LPHFDGRLFNVPS--VEECLSNVIWRCRGDA--IRNSVSGFA------RSLFTTEELH----GKNKEDMLEMVKKK-GFPYEQSVPNWALEGSMVKR----------------------------------------------------------------------tlvrmaavdqktg--etvevvrtrtr--CKDRGITEFS---------DEN---LALVRD-------    256722828       Nectria haematococca mpVI 77-13-4                       eukaryota>fungi>ascomycota  SNOG\_06471\_Pnod\_169606842                             PNIPLAERMKKYEAVYDTTLPS-NSPIILRLDGHNFSR-FTS--HFARP---FDERIHSAMLSTCTSLLTF--F-PSATLAYTQSDEITLIFPSG----------------VGAFGERVQKLSSLAASYTSVNFVKHLIAAVDAQPEPALKGEGGKDVLW---------------------------TAHFDARIFAVPS--IEEALNNLLWRCRNDA--VRNSVSSFA------RTMYSTKEMH----GKRAKELVAMMREEKGVVFEDAVPKWAIEGCLIKR----------------------------------------------------------------------eqvdhwgrngktg--ememtfrarvr--VEERGVREFG---------EEG---LRLVQE-------    169606842       Phaeosphaeria nodorum SN15                              eukaryota>fungi>ascomycota  PTRG\_02487\_Ptri\_189192963                             TTQSLASRMKSYESTFDHTLPL-TSPIILRLDGHGFSR-FTA--HFARP---FDQRIHLAMTRTSSDLLSY--F-PSATLAYTQSDEITLVFPSG----------------VQTFNSRVQKLSSIAASYCSVRFNKHLSAALRELTEPRVSGDVEEWLG----------------------------TAHFDARFFPVPN--VEEALNNLIWRCRNDA--VRNAVSGFA------RTMYTTAEMH----GKKTNELIEMMLQDKGVRFEEAVPKWAIEGCLIKR----------------------------------------------------------------------eqyeheglnmktg--ekektfrtrtr--VEERGVREFN---------DEG---LRLITD-------    189192963       Pyrenophora tritici-repentis Pt-1C-BFP                  eukaryota>fungi>ascomycota  PPL\_08738\_Ppal\_281203894                              DSVTLGDRMKQYEVSMNTLHITDNTPFIIRLDGHGFSK-FTK--NFVKP---WDIRVHNAMVETATVLMKE--F--NPTLVYTFSDEITLCFSSLPDQEYQERLIATQSQSLLPYNGKVQKLITLAAGIASTTFYKVITSQTYDSATEPKLTQYLAES-----------------------------LPHFDARIFTLPD--NQEIINNLVWRSVIDC--KRNSISGLA------QAHFPHKQIQ----GKGGKEMK-SMLLAKGIDYYKEP-MWYRFGVFLKK----------------------------------------------------------------------qyytldsvspvnn--qsvqsirskir--RDSFNIQHF----------PNA---LDYLTI-------    281203894       Polysphondylium pallidum PN500                          eukaryota>amoebozoa>mycetozoa>dictyosteliida  TTHERM\_00373760\_Tthe\_118352666                        SKKSIGDRMKEYELSARSKIDP-SLPAIMRIDGHSFSK-FTK--GLKKP---YDEWLHKLMVETTAALISE--F--SFNIGYTQSDEITLVYLPSFDKSGKLN--------DYPYANQIMKLVSLSSAFATNYFTRGIQRGIQDKTINLEDYQESTITKLQNP------------------------KCYFDSRIFNVPS--IQEVYSNIYWRSCYDC--IKNSVSMVA------YVHFPVKLTD----GLHTQAKIKKLLEEKNIDWNKDFSNHFKYGTFIKK----------------------------------------------------------------rqfeidlpleyqkfkkdss--nqkvnrt------ETVAFSYNFSGNKFN----EDT---KNILFE-------    118352666       Tetrahymena thermophila                                 eukaryota>alveolata>ciliophora  MA0817\_Mace\_20089701                                  ---------KTREIYAEMRCIP---PVVLRADGRNFKN-TLSGLGFEKP---YDKTFARAMADTAELFIKKSGL--SPLFAYTFSDEISFLFT------------------DLPFDGRVEKIDSVVASFLGSALTIKLRLEE---------------------------------------------PIAFDSRLVALQK---EEIPEYFHRRQ-LEA--WRNFVAS-------------------------------------------------------------------------------------------------------------------------------------------------------------------------------------------------    20089701        Methanosarcina acetivorans C2A                          archaea>euryarchaeota  Cyan7822DRAFT\_2032\_Csp.\_196256337                     KFEELDAKLRVFETGHDFCVLP-EIFMIARLDGRNFTRLTKDTHQFEAP---FDLKFRDYMVATVEHLMNC-GF--RVVYGYTQSDEISLLLHRDE---------------TT-FGRKLRKLNSVLAGEASAKLSLLLGT-----------------------------------------------LAAFDCRISQLPT--LNLVVDYFRI----------------------------------------------------------------------------------------------------------------------------------------------------------------------------------------------------------------    196256337       Cyanothece sp. PCC 7822                                 bacteria>cyanobacteria  COPEUT\_02780\_Ceut\_163816590                           -------MKDFYEQIPKTKLMR-RCPVAIRIDGKAFHT-YTR--NFKRP---FDSVFMRAMQETMKYLCEN--IQ-GCVLGYTQSDEITLILVDYEKIN-----------SSAWFDYEVQKLCSVSASMATMRFNQVFARLGR--------------------------------------------CLIQDASIYRRKR----------------------------------------------------------------------------------------------------------------------------------------------------------------------------------------------------------------------------    163816590       Coprococcus eutactus ATCC 27759                         bacteria>firmicutes  NAEGRDRAFT\_61112\_Ngru\_284097001                       ------LFEGQYDSKVNFDIAD-QQPIVMRFDGFHFKS-FTTNGDIFEKP--FDANIHWALLLAVRDLCLK-NLSMNPTLIYNCSDELTLLFSGQTSNNEKQKV------PNGIYRGRAQKLVSISTSFLTITFNYYLRKLLRANADSFESRLEYDMHNSNISKKSYQTEDFEKRRLVRQKLLDGDIFGNFDCRIYQLPT--ISHAIDYMIWRQ-IDCIRNSKTTLGSKFYA---QNEMNFLSAS-KIVEMVEQEKVHLLNMI--IPLRIDG-DVLDRAIRLV-----------------------------------------------------------------------------------------------------------------------------------------    284097001       Naegleria gruberi                                       eukaryota>heterolobosea  NEMVEDRAFT\_v1g147966\_Nvec\_156340600                   --------MRKNETLFDQHVLP-DNYIVVRLDGKGFTK-LTKESLDLEKP--FDIRFHDAMVATTKHLLTV-GF--KVIYAYTQSDEISLLIDK----------------DDNTFNRKVRKINSVLAGEASAFFSMYFNK-----------------------------------------------LSVLDCRTICIPN--IEMLLDYFCWRQ-EDA--HRNSLSAYCYWTLR-NNGNSYIEATKKTEKLSVSDKNELLFQH-GINYNSVP-SWQKRGVGMFYesidrkgin-------------------------------------------------------------------------------------------------------------------------------    156340600       Nematostella vectensis                                  eukaryota>metazoa>cnidaria  consensus/90%                                         .......b.b.......--h..-...hllRlDGp.Fpp.hsp...h.+P....D..h.phMs.ss..lh.p..h.....huas.SDEhohhh......................h..p..Kh.o..su.hss.a...h..................................................s.FDuRhh.hsp...p.l.sYh.WRp..-s....Nsb.s.s...h.....bs..ph.....s....pbp-hlbpp.slshsp.s...b+bG..h.b.....................................................................................................................p.......h..........  Mutated Residues with effect                          ............@................\*.................@............................#.....$..\*\*..............................\*..\*................$.....................................................\*.\*..............$...\*...#...\*$\*..\*@............................\*.@........@..........@...    The Mutated Residues marked are from the paper Jackman JE, Phizicky EM: **Identification of critical residues for G-1 addition and substrate recognition by tRNA(His) guanylyltransferase**. *Biochemistry* 2008, **47**(16):4817-4825.  \* (R27, D77, E78, R93, K96, D131, R133, R150, H155, N157, Y160, and K190),corresponding variants have defects in both G-1 addition and complementation  # (D153A) causes a significant growth defect that does not correlate with decreased G-1 addition activity, and the high level of activity  measured with this variant.   @ (E13, K44, N161, E192, N201, and K211) the alanine variants have substantially reduced activity in vitro, yet there is no identifiable phenotype of the corresponding yeast thg1 mutant strains.  $ Alterations at (G74, W113/I156, and Y146) result in a temperature-sensitive growth of the thg1 mutant strains.   # a single variant (D68A) demonstrated dramatically altered substrate specificity compared to the wild-type enzyme, which is rigorously specific for tRNAHis. |

  
  
  
  
  
Top  


---

Family
clusters of Thg1 domain containing proteins with domain
architectures   
  

```
# 223; Thg1 solos  
82617278 C4_0009 262 uncultured archaeon archaea conserved hypothetical protein [uncultured archaeon].   
268323173 BSM_02380 253 uncultured archaeon archaea conserved hypothetical protein containing tRNAHis   
126458698 Pcal_0070 225 Pyrobaculum calidifontis JCM 11548 archaea>crenarchaeota hypothetical protein Pcal_0070 [Pyrobaculum calidifontis JCM   
145590370 Pars_0105 196 Pyrobaculum arsenaticum DSM 13514 archaea>crenarchaeota hypothetical protein Pars_0105 [Pyrobaculum arsenaticum DSM 13514].   
156937279 Igni_0485 162 Ignicoccus hospitalis KIN4/I archaea>crenarchaeota hypothetical protein Igni_0485 [Ignicoccus hospitalis KIN4/I].   
18312250 PAE0886 212 Pyrobaculum aerophilum str. IM2 archaea>crenarchaeota hypothetical protein PAE0886 [Pyrobaculum aerophilum str. IM2].   
119872651 Pisl_1144 212 Pyrobaculum islandicum DSM 4184 archaea>crenarchaeota hypothetical protein Pisl_1144 [Pyrobaculum islandicum DSM 4184].   
124027917 Hbut_1048 238 Hyperthermus butylicus DSM 5456 archaea>crenarchaeota hypothetical protein Hbut_1048 [Hyperthermus butylicus DSM 5456].   
171184582 Tneu_0097 212 Thermoproteus neutrophilus V24Sta archaea>crenarchaeota hypothetical protein Tneu_0097 [Thermoproteus neutrophilus V24Sta].   
20093533 MK0093 262 Methanopyrus kandleri AV19 archaea>euryarchaeota hypothetical protein MK0093 [Methanopyrus kandleri AV19].   
288559933 thgL 252 Methanobrevibacter ruminantium M1 archaea>euryarchaeota tRNA(His) guanylyltransferase ThgL [Methanobrevibacter ruminantium   
282165226 MCP_2556 247 Methanocella paludicola SANAE archaea>euryarchaeota hypothetical protein MCP_2556 [Methanocella paludicola SANAE].   
116754185 Mthe_0875 235 Methanosaeta thermophila PT archaea>euryarchaeota hypothetical protein Mthe_0875 [Methanosaeta thermophila PT].   
84489939 Msp_1147 237 Methanosphaera stadtmanae DSM 3091 archaea>euryarchaeota hypothetical protein Msp_1147 [Methanosphaera stadtmanae DSM 3091].   
15678990 MTH972 246 Methanothermobacter thermautotrophicus str. Delta H archaea>euryarchaeota hypothetical protein MTH972 [Methanothermobacter thermautotrophicus   
20089701 MA0817 141 Methanosarcina acetivorans C2A archaea>euryarchaeota hypothetical protein MA0817 [Methanosarcina acetivorans C2A].   
154151809 Mboo_2270 250 Candidatus Methanoregula boonei 6A8 archaea>euryarchaeota hypothetical protein Mboo_2270 [Candidatus Methanoregula boonei   
261349483 METSMIF1_02117 239 Methanobrevibacter smithii DSM 2374 archaea>euryarchaeota tRNAHis guanylyltransferase family protein [Methanobrevibacter   
222445979 METSMIALI_01627 239 Methanobrevibacter smithii DSM 2375 archaea>euryarchaeota hypothetical protein METSMIALI_01627 [Methanobrevibacter smithii   
148642523 Msm_0463 239 Methanobrevibacter smithii ATCC 35061 archaea>euryarchaeota tRNA(His) guanylyltransferase [Methanobrevibacter smithii ATCC   
124485581 Mlab_0759 240 Methanocorpusculum labreanum Z archaea>euryarchaeota hypothetical protein Mlab_0759 [Methanocorpusculum labreanum Z].   
88601907 Mhun_0610 241 Methanospirillum hungatei JF-1 archaea>euryarchaeota hypothetical protein Mhun_0610 [Methanospirillum hungatei JF-1].   
284162827 Arcpr_1732 246 Archaeoglobus profundus DSM 5631 archaea>euryarchaeota protein of unknown function DUF549 [Archaeoglobus profundus DSM   
147920979 RCIX455 241 uncultured methanogenic archaeon RC-I archaea>euryarchaeota hypothetical protein RCIX455 [uncultured methanogenic archaeon   
91773429 Mbur_1464 243 Methanococcoides burtonii DSM 6242 archaea>euryarchaeota hypothetical protein Mbur_1464 [Methanococcoides burtonii DSM   
73669253 Mbar_A1746 243 Methanosarcina barkeri str. Fusaro archaea>euryarchaeota hypothetical protein Mbar_A1746 [Methanosarcina barkeri str.   
294496410 Mmah_1763 243 Methanohalophilus mahii DSM 5219 archaea>euryarchaeota tRNA(His)-5'-guanylyltransferase [Methanohalophilus mahii DSM   
219850835 Mpal_0151 243 Methanosphaerula palustris E1-9c archaea>euryarchaeota protein of unknown function DUF549 [Candidatus Methanosphaerula   
21228066 MM_1964 243 Methanosarcina mazei Go1 archaea>euryarchaeota hypothetical protein MM_1964 [Methanosarcina mazei Go1].   
126178352 Memar_0402 244 Methanoculleus marisnigri JR1 archaea>euryarchaeota hypothetical protein Memar_0402 [Methanoculleus marisnigri JR1].   
170290817 Kcr_1204 249 Candidatus Korarchaeum cryptofilum OPF8 archaea>korarchaeota hypothetical protein Kcr_1204 [Candidatus Korarchaeum cryptofilum   
196256337 Cyan7822DRAFT_2032 141 Cyanothece sp. PCC 7822 bacteria>cyanobacteria tRNAHis guanylyltransferase family protein [Cyanothece sp. PCC   
171913885 VspiD_010100021940 159 Verrucomicrobium spinosum DSM 4136 bacteria>chlamydiae/verrucomicrobia tRNAHis guanylyltransferase family protein [Verrucomicrobium   
153872919 BGP_4908 214 Beggiatoa sp. PS bacteria>proteobacteria>gammaproteobacteria protein of unknown function DUF549 [Beggiatoa sp. PS].   
54025916 nfa39460 229 Nocardia farcinica IFM 10152 bacteria>actinobacteria hypothetical protein nfa39460 [Nocardia farcinica IFM 10152].   
219882632 Achl_4028 238 Arthrobacter chlorophenolicus A6 bacteria>actinobacteria hypothetical protein Achl_4028 [Arthrobacter chlorophenolicus A6].   
269120695 Sterm_2087 241 Sebaldella termitidis ATCC 33386 bacteria>fusobacteria hypothetical protein Sterm_2087 [Sebaldella termitidis ATCC 33386].   
75758411 RBTH_06728 245 Bacillus thuringiensis serovar israelensis ATCC 35646 bacteria>firmicutes Hypothetical protein RBTH_06728 [Bacillus thuringiensis serovar   
162454339 sce6062 248 Sorangium cellulosum 'So ce 56' bacteria>proteobacteria>deltaproteobacteria hypothetical protein sce6062 [Sorangium cellulosum 'So ce 56'].   
124008957 M23134_06676 250 Microscilla marina ATCC 23134 bacteria>bacteroidetes/chlorobi conserved protein [Microscilla marina ATCC 23134].   
252128517 CORMA0001_0334 250 Corynebacterium matruchotii ATCC 14266 bacteria>actinobacteria trnahis guanylyltransferase family protein [Corynebacterium   
225022489 CORMATOL_02529 250 Corynebacterium matruchotii ATCC 33806 bacteria>actinobacteria hypothetical protein CORMATOL_02529 [Corynebacterium matruchotii   
255067726 NEISICOT_02755 251 Neisseria sicca ATCC 29256 bacteria>proteobacteria>betaproteobacteria tRNA(His) guanylyltransferase family protein [Neisseria sicca ATCC   
225075026 NEIFLAOT_00025 252 Neisseria flavescens NRL30031/H210 bacteria>proteobacteria>betaproteobacteria hypothetical protein NEIFLAOT_00025 [Neisseria flavescens   
159900562 Haur_4049 252 Herpetosiphon aurantiacus ATCC 23779 bacteria>chloroflexi hypothetical protein Haur_4049 [Herpetosiphon aurantiacus ATCC   
158336895 AM1_3767 253 Acaryochloris marina MBIC11017 bacteria>cyanobacteria tRNAHis guanylyltransferase family protein [Acaryochloris marina   
239627528 CBFG_01250 253 Clostridiales bacterium 1_7_47FAA bacteria>firmicutes tRNAHis guanylyltransferase family protein [Clostridiales bacterium   
294671196 NEIELOOT_02904 255 Neisseria elongata subsp. glycolytica ATCC 29315 bacteria>proteobacteria>betaproteobacteria hypothetical protein NEIELOOT_02904 [Neisseria elongata subsp.   
256424765 Cpin_5797 255 Chitinophaga pinensis DSM 2588 bacteria>bacteroidetes/chlorobi tRNA(His)-5'-guanylyltransferase [Chitinophaga pinensis DSM 2588].   
254412407 MC7420_6362 255 Microcoleus chthonoplastes PCC 7420 bacteria>cyanobacteria tRNAHis guanylyltransferase superfamily [Microcoleus chthonoplastes   
258545199 HMPREF0198_1468 256 Cardiobacterium hominis ATCC 15826 bacteria>proteobacteria>gammaproteobacteria tRNAHis guanylyltransferase family protein [Cardiobacterium hominis   
229539832 PlimDRAFT_36970 258 Planctomyces limnophilus DSM 3776 bacteria>planctomycetes tRNA(His)-5'-guanylyltransferase [Planctomyces limnophilus DSM   
108762077 MXAN_5968 267 Myxococcus xanthus DK 1622 bacteria>proteobacteria>deltaproteobacteria tRNAHis guanylyltransferase family protein [Myxococcus xanthus DK   
149920560 PPSIR1_23574 274 Plesiocystis pacifica SIR-1 bacteria>proteobacteria>deltaproteobacteria hypothetical protein PPSIR1_23574 [Plesiocystis pacifica SIR-1].   
168698293 GobsU_010100002157 279 Gemmata obscuriglobus UQM 2246 bacteria>planctomycetes hypothetical protein GobsU_02157 [Gemmata obscuriglobus UQM 2246].   
291529569 ERE_34100 285 Eubacterium rectale M104/1 bacteria>firmicutes Uncharacterized conserved protein [Eubacterium rectale M104/1].   
228982997 bthur0002_61310 353 Bacillus thuringiensis Bt407 bacteria>firmicutes hypothetical protein bthur0002_61310 [Bacillus thuringiensis   
  
55819137 MIMI_R259 292 Acanthamoeba polyphaga mimivirus dsDNA viruses, no RNA stage hypothetical protein MIMI_R259 [Acanthamoeba polyphaga mimivirus].   
189490470 201phi2-1p319 270 Pseudomonas phage 201phi2-1 dsDNA viruses, no RNA stage>caudovirales putative Thg1 [Pseudomonas phage 201phi2-1].   
  
221052852 PKH_031670 282 Plasmodium knowlesi strain H eukaryota>alveolata>apicomplexa tRNA guanylyltransferase [Plasmodium knowlesi strain H].   
68070617 PB000744.00.0 148 Plasmodium berghei str. ANKA eukaryota>alveolata>apicomplexa hypothetical protein [Plasmodium berghei strain ANKA].   
209557756 CMU_028750 288 Cryptosporidium muris RN66 eukaryota>alveolata>apicomplexa tRNA(His) guanylyltransferase, putative [Cryptosporidium muris   
156093512 PVX_096205 364 Plasmodium vivax SaI-1 eukaryota>alveolata>apicomplexa hypothetical protein [Plasmodium vivax SaI-1].   
83285854 PY02052 356 Plasmodium yoelii yoelii str. 17XNL eukaryota>alveolata>apicomplexa hypothetical protein [Plasmodium yoelii yoelii str. 17XNL].   
124511996 PF07_0095 299 Plasmodium falciparum 3D7 eukaryota>alveolata>apicomplexa hypothetical protein [Plasmodium falciparum 3D7].   
84999512 TA19625 330 Theileria annulata strain Ankara eukaryota>alveolata>apicomplexa hypothetical protein [Theileria annulata].   
156086032 BBOV_IV004960 324 Babesia bovis T2Bo eukaryota>alveolata>apicomplexa tRNA-His guanylyltransferase [Babesia bovis T2Bo].   
71032267 TP01_0248 293 Theileria parva strain Muguga eukaryota>alveolata>apicomplexa hypothetical protein [Theileria parva strain Muguga].   
126643977 cgd1_1990 296 Cryptosporidium parvum Iowa II eukaryota>alveolata>apicomplexa hypothetical protein [Cryptosporidium parvum Iowa II].   
118358858 TTHERM_00085230 334 Tetrahymena thermophila eukaryota>alveolata>ciliophora tRNAHis guanylyltransferase family protein [Tetrahymena   
118352666 TTHERM_00373760 322 Tetrahymena thermophila eukaryota>alveolata>ciliophora hypothetical protein TTHERM_00373760 [Tetrahymena thermophila].   
145533455 GSPATT00002474001 290 Paramecium tetraurelia strain d4-2 eukaryota>alveolata>ciliophora hypothetical protein [Paramecium tetraurelia strain d4-2].   
66814690 DDB_G0279743 415 Dictyostelium discoideum AX4 eukaryota>amoebozoa>mycetozoa>dictyosteliida hypothetical protein DDB_G0279743 [Dictyostelium discoideum AX4].   
66819813 DDB_G0275625 291 Dictyostelium discoideum AX4 eukaryota>amoebozoa>mycetozoa>dictyosteliida hypothetical protein DDB_G0275625 [Dictyostelium discoideum AX4].   
66805193 DDB_G0289191 278 Dictyostelium discoideum AX4 eukaryota>amoebozoa>mycetozoa>dictyosteliida hypothetical protein DDB_G0289191 [Dictyostelium discoideum AX4].   
281203894 PPL_08738 308 Polysphondylium pallidum PN500 eukaryota>amoebozoa>mycetozoa>dictyosteliida hypothetical protein PPL_08738 [Polysphondylium pallidum PN500].   
66802352 thg1 256 Dictyostelium discoideum AX4 eukaryota>amoebozoa>mycetozoa>dictyosteliida tRNA-histidine guanylyltransferase 1 [Dictyostelium discoideum   
167518403 MONBRDRAFT_31372 330 Monosiga brevicollis MX1 eukaryota>choanoflagellida hypothetical protein [Monosiga brevicollis MX1].   
119186387 CIMG_03241 287 Coccidioides immitis RS eukaryota>fungi>ascomycota hypothetical protein CIMG_03241 [Coccidioides immitis RS].   
189197179 PTRG_04594 207 Pyrenophora tritici-repentis Pt-1C-BFP eukaryota>fungi>ascomycota histidyl tRNA-specific guanylyltransferase [Pyrenophora   
225677608 PABG_05979 335 Paracoccidioides brasiliensis Pb03 eukaryota>fungi>ascomycota conserved hypothetical protein [Paracoccidioides brasiliensis   
119494994 NFIA_010790 291 Neosartorya fischeri NRRL 181 eukaryota>fungi>ascomycota tRNAHis guanylyltransferase, putative [Neosartorya fischeri NRRL   
145230810 An01g12630 281 Aspergillus niger CBS 513.88 eukaryota>fungi>ascomycota hypothetical protein An01g12630 [Aspergillus niger].   
256722828 NECHADRAFT_87153 276 Nectria haematococca mpVI 77-13-4 eukaryota>fungi>ascomycota hypothetical protein NECHADRAFT_87153 [Nectria haematococca mpVI   
255941868 Pc16g14050 275 Penicillium chrysogenum Wisconsin 54-1255 eukaryota>fungi>ascomycota Pc16g14050 [Penicillium chrysogenum Wisconsin 54-1255].   
226295250 PADG_06749 291 Paracoccidioides brasiliensis Pb18 eukaryota>fungi>ascomycota tRNA(His) guanylyltransferase [Paracoccidioides brasiliensis Pb18].   
149234768 LELG_05489 273 Lodderomyces elongisporus NRRL YB-4239 eukaryota>fungi>ascomycota hypothetical protein LELG_05489 [Lodderomyces elongisporus NRRL   
171695070 PODANSg09507 273 Podospora anserina DSM 980 eukaryota>fungi>ascomycota unnamed protein product [Podospora anserina].   
189192963 PTRG_02487 272 Pyrenophora tritici-repentis Pt-1C-BFP eukaryota>fungi>ascomycota conserved hypothetical protein [Pyrenophora tritici-repentis   
156054634 SS1G_06165 226 Sclerotinia sclerotiorum 1980 UF-70 eukaryota>fungi>ascomycota hypothetical protein SS1G_06165 [Sclerotinia sclerotiorum 1980].   
254566669 PAS_chr1-4_0323 271 Pichia pastoris GS115 eukaryota>fungi>ascomycota tRNAHis guanylyltransferase [Pichia pastoris GS115].   
6321461 THG1 237 Saccharomyces cerevisiae eukaryota>fungi>ascomycota Thg1p [Saccharomyces cerevisiae].   
50303737 KLLA0B06237g 237 Kluyveromyces lactis NRRL Y-1140 eukaryota>fungi>ascomycota unnamed protein product [Kluyveromyces lactis].   
50290577 CAGL0J00209g 237 Candida glabrata CBS 138 eukaryota>fungi>ascomycota unnamed protein product [Candida glabrata].   
45185260 AGOS_ABR031C 237 Ashbya gossypii ATCC 10895 eukaryota>fungi>ascomycota ABR031Cp [Ashbya gossypii ATCC 10895].   
259146527 EC1118_1G1_3257g 237 Saccharomyces cerevisiae EC1118 eukaryota>fungi>ascomycota Thg1p [Saccharomyces cerevisiae EC1118].   
255718999 KLTH0G17270g 237 Lachancea thermotolerans CBS 6340 eukaryota>fungi>ascomycota KLTH0G17270p [Lachancea thermotolerans].   
150865885 PICST_60761 268 Pichia stipitis CBS 6054 eukaryota>fungi>ascomycota hypothetical protein PICST_60761 [Pichia stipitis CBS 6054].   
169606842 SNOG_06471 268 Phaeosphaeria nodorum SN15 eukaryota>fungi>ascomycota hypothetical protein SNOG_06471 [Phaeosphaeria nodorum SN15].   
238883228 CAWG_05412 268 Candida albicans WO-1 eukaryota>fungi>ascomycota hypothetical protein CAWG_05412 [Candida albicans WO-1].   
241957021 CD36_70100 268 Candida dubliniensis CD36 eukaryota>fungi>ascomycota tRNA(His) guanylyltransferase, putative; tRNA-histidine   
240274622 HCDG_07873 292 Ajellomyces capsulatus H143 eukaryota>fungi>ascomycota tRNA(His) guanylyltransferase [Ajellomyces capsulatus H143].   
46442219 THG1 268 Candida albicans SC5314 eukaryota>fungi>ascomycota likely histidyl tRNA-specific guanylyltransferase [Candida albicans   
254577809 ZYRO0A12144g 237 Zygosaccharomyces rouxii CBS 732 eukaryota>fungi>ascomycota ZYRO0A12144p [Zygosaccharomyces rouxii].   
260946739 CLUG_03111 267 Clavispora lusitaniae ATCC 42720 eukaryota>fungi>ascomycota hypothetical protein CLUG_03111 [Clavispora lusitaniae ATCC 42720].   
289622466 SMAC_05003 267 Sordaria macrospora eukaryota>fungi>ascomycota unnamed protein product [Sordaria macrospora].   
261358866 VDBG_07404 288 Verticillium albo-atrum VaMs.102 eukaryota>fungi>ascomycota tRNA(His) guanylyltransferase [Verticillium albo-atrum VaMs.102].   
146422179 PGUG_00408 265 Pichia guilliermondii ATCC 6260 eukaryota>fungi>ascomycota hypothetical protein PGUG_00408 [Pichia guilliermondii ATCC 6260].   
164429701 NCU02105 265 Neurospora crassa OR74A eukaryota>fungi>ascomycota hypothetical protein NCU02105 [Neurospora crassa OR74A].   
190344604 PGUG_00408 265 Pichia guilliermondii ATCC 6260 eukaryota>fungi>ascomycota hypothetical protein PGUG_00408 [Pichia guilliermondii ATCC 6260].   
256734706 NECHADRAFT_30739 265 Nectria haematococca mpVI 77-13-4 eukaryota>fungi>ascomycota hypothetical protein NECHADRAFT_30739 [Nectria haematococca mpVI   
294659006 DEHA2F22880g 265 Debaryomyces hansenii CBS767 eukaryota>fungi>ascomycota DEHA2F22880p [Debaryomyces hansenii CBS767].   
50425487 DEHA0F24321g 265 Debaryomyces hansenii CBS767 eukaryota>fungi>ascomycota hypothetical protein DEHA0F24321g [Debaryomyces hansenii CBS767].   
213409163 SJAG_04233 263 Schizosaccharomyces japonicus yFS275 eukaryota>fungi>ascomycota tRNA(His) guanylyltransferase [Schizosaccharomyces japonicus   
238837882 MCYG_00432 292 Microsporum canis CBS 113480 eukaryota>fungi>ascomycota histidine tRNA 5'-guanylyltransferase [Microsporum canis CBS   
85111778 NCU02105.1 293 Neurospora crassa OR74A eukaryota>fungi>ascomycota hypothetical protein [Neurospora crassa OR74A].   
19075481 SPCC63.07 261 Schizosaccharomyces pombe eukaryota>fungi>ascomycota tRNA guanylyltransferase (predicted) [Schizosaccharomyces pombe   
156843110 Kpol_526p19 238 Vanderwaltozyma polyspora DSM 70294 eukaryota>fungi>ascomycota hypothetical protein Kpol_526p19 [Vanderwaltozyma polyspora DSM   
239612828 BDCG_04935 239 Ajellomyces dermatitidis ER-3 eukaryota>fungi>ascomycota tRNA(His) guanylyltransferase [Ajellomyces dermatitidis ER-3].   
255731634 CTRG_05039 268 Candida tropicalis MYA-3404 eukaryota>fungi>ascomycota hypothetical protein CTRG_05039 [Candida tropicalis MYA-3404].   
212533511 PMAA_074380 312 Penicillium marneffei ATCC 18224 eukaryota>fungi>ascomycota tRNAHis guanylyltransferase Thg1, putative [Penicillium marneffei   
70996154 AFUA_1G14630 372 Aspergillus fumigatus Af293 eukaryota>fungi>ascomycota tRNAHis guanylyltransferase Thg1 [Aspergillus fumigatus Af293].   
159131585 AFUB_014160 374 Aspergillus fumigatus A1163 eukaryota>fungi>ascomycota tRNAHis guanylyltransferase Thg1, putative [Aspergillus fumigatus   
154320945 BC1G_01347 153 Botryotinia fuckeliana B05.10 eukaryota>fungi>ascomycota hypothetical protein BC1G_01347 [Botryotinia fuckeliana B05.10].   
226278784 PAAG_05399 368 Paracoccidioides brasiliensis Pb01 eukaryota>fungi>ascomycota conserved hypothetical protein [Paracoccidioides brasiliensis   
242778279 TSTA_094890 290 Talaromyces stipitatus ATCC 10500 eukaryota>fungi>ascomycota tRNAHis guanylyltransferase Thg1, putative [Talaromyces stipitatus   
46108064 FG00914.1 253 Gibberella zeae PH-1 eukaryota>fungi>ascomycota hypothetical protein FG00914.1 [Gibberella zeae PH-1].   
121701067 ACLA_020800 296 Aspergillus clavatus NRRL 1 eukaryota>fungi>ascomycota tRNAHis guanylyltransferase, putative [Aspergillus clavatus NRRL   
67517047 AN0804.2 255 Aspergillus nidulans FGSC A4 eukaryota>fungi>ascomycota hypothetical protein AN0804.2 [Aspergillus nidulans FGSC A4].   
169770385 AO090003000484 295 Aspergillus oryzae RIB40 eukaryota>fungi>ascomycota hypothetical protein [Aspergillus oryzae RIB40].   
170108182 LACBIDRAFT_252840 256 Laccaria bicolor S238N-H82 eukaryota>fungi>basidiomycota predicted protein [Laccaria bicolor S238N-H82].   
242205948 POSPLDRAFT_87675 283 Postia placenta Mad-698-R eukaryota>fungi>basidiomycota predicted protein [Postia placenta Mad-698-R].   
58265566 CNC02800 285 Cryptococcus neoformans var. neoformans JEC21 eukaryota>fungi>basidiomycota tRNA guanylyltransferase [Cryptococcus neoformans var. neoformans   
242214759 POSPLDRAFT_88866 293 Postia placenta Mad-698-R eukaryota>fungi>basidiomycota predicted protein [Postia placenta Mad-698-R].   
169860017 CC1G_06232 353 Coprinopsis cinerea okayama7#130 eukaryota>fungi>basidiomycota hypothetical protein CC1G_06232 [Coprinopsis cinerea okayama7#130].   
238663948 Smp_073990 113 Schistosoma mansoni eukaryota>metazoa conserved hypothetical protein [Schistosoma mansoni].   
198436739 LOC100179812 279 Ciona intestinalis eukaryota>metazoa PREDICTED: similar to interphase cytoplasmic foci protein 45 [Ciona   
241096199 IscW_ISCW001226 258 Ixodes scapularis eukaryota>metazoa conserved hypothetical protein [Ixodes scapularis].   
260828313 BRAFLDRAFT_60263 270 Branchiostoma floridae eukaryota>metazoa hypothetical protein BRAFLDRAFT_60263 [Branchiostoma floridae].   
221135876 LOC100205752 259 Hydra magnipapillata eukaryota>metazoa>cnidaria PREDICTED: similar to predicted protein [Hydra magnipapillata].   
156403638 NEMVEDRAFT_v1g84802 260 Nematostella vectensis eukaryota>metazoa>cnidaria predicted protein [Nematostella vectensis].   
156340600 NEMVEDRAFT_v1g147966 207 Nematostella vectensis eukaryota>metazoa>cnidaria hypothetical protein NEMVEDRAFT_v1g147966 [Nematostella vectensis].   
225712866 THG1 317 Lepeophtheirus salmonis eukaryota>metazoa>crustacea Probable tRNAHis guanylyltransferase [Lepeophtheirus salmonis].   
225719698 THG1 296 Caligus clemensi eukaryota>metazoa>crustacea Probable tRNAHis guanylyltransferase [Caligus clemensi].   
225711510 THG1 307 Caligus rogercresseyi eukaryota>metazoa>crustacea Probable tRNAHis guanylyltransferase [Caligus rogercresseyi].   
225712230 THG1 300 Lepeophtheirus salmonis eukaryota>metazoa>crustacea Probable tRNAHis guanylyltransferase [Lepeophtheirus salmonis].   
225710266 THG1 285 Caligus rogercresseyi eukaryota>metazoa>crustacea Probable tRNAHis guanylyltransferase [Caligus rogercresseyi].   
Dpul1000000854 Dpul1000000854 257 Daphnia pulex eukaryota>metazoa>crustacea e_gw1.1.708.1   
115921197 LOC587315 265 Strongylocentrotus purpuratus eukaryota>metazoa>echinodermata PREDICTED: hypothetical protein [Strongylocentrotus purpuratus].   
291224944 LOC100374565 341 Saccoglossus kowalevskii eukaryota>metazoa>hemichordata PREDICTED: CG4103-like [Saccoglossus kowalevskii].   
193673956 LOC100165160 272 Acyrthosiphon pisum eukaryota>metazoa>hexapoda PREDICTED: similar to predicted protein isoform 2 [Acyrthosiphon   
195155911 Dper\GL25730 293 Drosophila persimilis eukaryota>metazoa>hexapoda GL25730 [Drosophila persimilis].   
195474109 Dyak\GE24732 287 Drosophila yakuba eukaryota>metazoa>hexapoda GE24732 [Drosophila yakuba].   
19921364 CG4103 286 Drosophila melanogaster eukaryota>metazoa>hexapoda CG4103 [Drosophila melanogaster].   
242016870 Phum_PHUM410370 252 Pediculus humanus corporis eukaryota>metazoa>hexapoda conserved hypothetical protein [Pediculus humanus corporis].   
195579310 Dsim\GD21976 287 Drosophila simulans eukaryota>metazoa>hexapoda GD21976 [Drosophila simulans].   
114051932 LOC692885 301 Bombyx mori eukaryota>metazoa>hexapoda interphase cyctoplasmic foci protein 45 [Bombyx mori].   
289742577 - 306 Glossina morsitans morsitans eukaryota>metazoa>hexapoda uncharacterized conserved protein [Glossina morsitans morsitans].   
195338513 Dsec\GM14588 287 Drosophila sechellia eukaryota>metazoa>hexapoda GM14588 [Drosophila sechellia].   
158300966 AgaP_AGAP011752 307 Anopheles gambiae str. PEST eukaryota>metazoa>hexapoda AGAP011752-PA [Anopheles gambiae str. PEST].   
195030226 Dgri\GH10821 286 Drosophila grimshawi eukaryota>metazoa>hexapoda GH10821 [Drosophila grimshawi].   
195437103 Dwil\GK18307 282 Drosophila willistoni eukaryota>metazoa>hexapoda GK18307 [Drosophila willistoni].   
239788252 ACYPI006122 272 Acyrthosiphon pisum eukaryota>metazoa>hexapoda ACYPI006122 [Acyrthosiphon pisum].   
170028733 CpipJ_CPIJ000684 312 Culex quinquefasciatus eukaryota>metazoa>hexapoda tRNA(His) guanylyltransferase [Culex quinquefasciatus].   
157138643 AaeL_AAEL014040 314 Aedes aegypti eukaryota>metazoa>hexapoda hypothetical protein AaeL_AAEL014040 [Aedes aegypti].   
156547518 LOC100117826 323 Nasonia vitripennis eukaryota>metazoa>hexapoda PREDICTED: similar to interphase cyctoplasmic foci protein 45   
195385629 Dvir\GJ16007 278 Drosophila virilis eukaryota>metazoa>hexapoda GJ16007 [Drosophila virilis].   
194857325 Dere\GG24229 287 Drosophila erecta eukaryota>metazoa>hexapoda GG24229 [Drosophila erecta].   
91083329 LOC663767 291 Tribolium castaneum eukaryota>metazoa>hexapoda PREDICTED: similar to interphase cyctoplasmic foci protein 45   
195115597 Dmoj\GI17333 284 Drosophila mojavensis eukaryota>metazoa>hexapoda GI17333 [Drosophila mojavensis].   
194758495 Dana\GF14997 286 Drosophila ananassae eukaryota>metazoa>hexapoda GF14997 [Drosophila ananassae].   
125987191 Dpse\GA17959 293 Drosophila pseudoobscura pseudoobscura eukaryota>metazoa>hexapoda GA17959 [Drosophila pseudoobscura pseudoobscura].   
190579964 TRIADDRAFT_32525 245 Trichoplax adhaerens eukaryota>metazoa>placozoa hypothetical protein TRIADDRAFT_32525 [Trichoplax adhaerens].   
196003010 TRIADDRAFT_55308 301 Trichoplax adhaerens eukaryota>metazoa>placozoa hypothetical protein TRIADDRAFT_55308 [Trichoplax adhaerens].   
149412544 LOC100075219 269 Ornithorhynchus anatinus eukaryota>metazoa>vertebrata PREDICTED: hypothetical protein [Ornithorhynchus anatinus].   
12853824 - 238 Mus musculus eukaryota>metazoa>vertebrata unnamed protein product [Mus musculus].   
57085243 LOC479315 398 Canis lupus familiaris eukaryota>metazoa>vertebrata PREDICTED: similar to interphase cyctoplasmic foci protein 45   
147904555 thg1l 286 Xenopus laevis eukaryota>metazoa>vertebrata tRNA-histidine guanylyltransferase 1-like [Xenopus laevis].   
71895317 THG1L 269 Gallus gallus eukaryota>metazoa>vertebrata interphase cytoplasmic foci protein 45 [Gallus gallus].   
126290678 LOC100025355 269 Monodelphis domestica eukaryota>metazoa>vertebrata PREDICTED: hypothetical protein [Monodelphis domestica].   
12840654 - 150 Mus musculus eukaryota>metazoa>vertebrata unnamed protein product [Mus musculus].   
224067593 LOC100223262 269 Taeniopygia guttata eukaryota>metazoa>vertebrata PREDICTED: interphase cytoplasmic foci protein 45 [Taeniopygia   
109079563 LOC715853 298 Macaca mulatta eukaryota>metazoa>vertebrata PREDICTED: similar to interphase cyctoplasmic foci protein 45   
114603135 LOC462226 298 Pan troglodytes eukaryota>metazoa>vertebrata PREDICTED: interphase cytoplasmic foci protein 45 [Pan   
122692489 THG1L 298 Bos taurus eukaryota>metazoa>vertebrata probable tRNA(His) guanylyltransferase [Bos taurus].   
124377988 Thg1l 298 Mus musculus eukaryota>metazoa>vertebrata probable tRNA(His) guanylyltransferase [Mus musculus].   
149726693 LOC100071194 298 Equus caballus eukaryota>metazoa>vertebrata PREDICTED: similar to Probable tRNA(His) guanylyltransferase   
281345114 PANDA_000301 298 Ailuropoda melanoleuca eukaryota>metazoa>vertebrata hypothetical protein PANDA_000301 [Ailuropoda melanoleuca].   
291387726 LOC100353509 298 Oryctolagus cuniculus eukaryota>metazoa>vertebrata PREDICTED: CG4103-like [Oryctolagus cuniculus].   
62078661 Thg1l 298 Rattus norvegicus eukaryota>metazoa>vertebrata probable tRNA(His) guanylyltransferase [Rattus norvegicus].   
62530979 Thg1l 298 Mus musculus eukaryota>metazoa>vertebrata Thg1l protein [Mus musculus].   
62897799 - 298 Homo sapiens eukaryota>metazoa>vertebrata interphase cyctoplasmic foci protein 45 variant [Homo sapiens].   
26383091 - 279 Mus musculus eukaryota>metazoa>vertebrata unnamed protein product [Mus musculus].   
7020726 - 298 Homo sapiens eukaryota>metazoa>vertebrata unnamed protein product [Homo sapiens].   
89242148 THG1L 298 Homo sapiens eukaryota>metazoa>vertebrata probable tRNA(His) guanylyltransferase [Homo sapiens].   
229366028 THG1 299 Anoplopoma fimbria eukaryota>metazoa>vertebrata>actinopterygii Probable tRNAHis guanylyltransferase [Anoplopoma fimbria].   
47216813 GSTEN:00031660:G:001 277 Tetraodon nigroviridis eukaryota>metazoa>vertebrata>actinopterygii unnamed protein product [Tetraodon nigroviridis].   
259089291 yg1g 299 Oncorhynchus mykiss eukaryota>metazoa>vertebrata>actinopterygii YGR024C [Oncorhynchus mykiss].   
55925403 zgc:101609 269 Danio rerio eukaryota>metazoa>vertebrata>actinopterygii interphase cytoplasmic foci protein 45 [Danio rerio].   
225716354 THG1 299 Esox lucius eukaryota>metazoa>vertebrata>actinopterygii Probable tRNAHis guanylyltransferase [Esox lucius].   
213514008 thg1 299 Salmo salar eukaryota>metazoa>vertebrata>actinopterygii Probable tRNAHis guanylyltransferase [Salmo salar].   
48309991 - 98 Arabidopsis thaliana eukaryota>viridiplantae At2g32320 [Arabidopsis thaliana].   
3831464 At2g32320 190 Arabidopsis thaliana eukaryota>viridiplantae hypothetical protein [Arabidopsis thaliana].   
3831463 At2g32330 297 Arabidopsis thaliana eukaryota>viridiplantae unknown protein [Arabidopsis thaliana].   
168038223 PHYPADRAFT_217293 258 Physcomitrella patens subsp. patens eukaryota>viridiplantae predicted protein [Physcomitrella patens subsp. patens].   
158283078 THG1 258 Chlamydomonas reinhardtii eukaryota>viridiplantae>chlorophyta histidine tRNA 5'-guanylyltransferase [Chlamydomonas reinhardtii].   
123437995 TVAG_470220 237 Trichomonas vaginalis G3 eukaryota>parabasalia tRNAHis guanylyltransferase family protein [Trichomonas vaginalis   
284097001 NAEGRDRAFT_61112 316 Naegleria gruberi eukaryota>heterolobosea predicted protein [Naegleria gruberi].   
# 12; Thgi+Thgi  
42569521 AT2G31580 567 Arabidopsis thaliana eukaryota>viridiplantae unknown protein [Arabidopsis thaliana].   
238479416 AT2G32320 537 Arabidopsis thaliana eukaryota>viridiplantae unknown protein [Arabidopsis thaliana].   
238479418 AT2G32320 532 Arabidopsis thaliana eukaryota>viridiplantae unknown protein [Arabidopsis thaliana].   
147819926 VITISV_031884 530 Vitis vinifera eukaryota>viridiplantae hypothetical protein [Vitis vinifera].   
225438521 LOC100264494 528 Vitis vinifera eukaryota>viridiplantae PREDICTED: hypothetical protein [Vitis vinifera].   
186504734 AT2G32320 525 Arabidopsis thaliana eukaryota>viridiplantae unknown protein [Arabidopsis thaliana].   
218197162 OsI_20769 524 Oryza sativa Indica Group eukaryota>viridiplantae hypothetical protein OsI_20769 [Oryza sativa Indica Group].   
222632362 OsJ_19344 524 Oryza sativa Japonica Group eukaryota>viridiplantae hypothetical protein OsJ_19344 [Oryza sativa Japonica Group].   
255578805 RCOM_0075620 524 Ricinus communis eukaryota>viridiplantae conserved hypothetical protein [Ricinus communis].   
115465113 Os05g0535500 519 Oryza sativa Japonica Group eukaryota>viridiplantae Os05g0535500 [Oryza sativa (japonica cultivar-group)].   
20198069 At2g31580 495 Arabidopsis thaliana eukaryota>viridiplantae unknown protein [Arabidopsis thaliana].   
224065048 POPTRDRAFT_409100 482 Populus trichocarpa eukaryota>viridiplantae predicted protein [Populus trichocarpa].   
45680430 OJ1014_C08.12 399 Oryza sativa Japonica Group eukaryota>viridiplantae putative tRNAHis guanylyltransferase [Oryza sativa Japonica Group].   
# 1; false fusion; glycosyl hydrolase family 17+Thgi+Thgi  
270232206 GSVIVT01022253001 1073 Vitis vinifera eukaryota>viridiplantae unnamed protein product [Vitis vinifera].   
# 1; false fusion;PaaI_Thioesterase+Thgi  
115492265 ATEG_00674 427 Aspergillus terreus NIH2624 eukaryota>fungi>ascomycota hypothetical protein ATEG_00674 [Aspergillus terreus NIH2624].   
# 1; false fusion;Thgi+ST Protein Kinase (2jav)  
281212173 thg1 731 Polysphondylium pallidum PN500 eukaryota>amoebozoa>mycetozoa>dictyosteliida tRNA-histidine guanylyltransferase 1 [Polysphondylium pallidum   
# 1;  
288922849 FrEUN1fDRAFT_6712 247 Frankia sp. EUN1f bacteria>actinobacteria hypothetical protein FrEUN1fDRAFT_6712 [Frankia sp. EUN1f].   
163816590 COPEUT_02780 128 Coprococcus eutactus ATCC 27759 bacteria>firmicutes hypothetical protein COPEUT_02780 [Coprococcus eutactus ATCC   
149908211 PE36_01857 288 Moritella sp. PE36 bacteria>proteobacteria>gammaproteobacteria hypothetical protein PE36_01857 [Moritella sp. PE36].   
284085645 NAEGRDRAFT_72795 112 Naegleria gruberi eukaryota>heterolobosea predicted protein [Naegleria gruberi].   
261194434 BDBG_06470 349 Ajellomyces dermatitidis SLH14081 eukaryota>fungi>ascomycota tRNA(His) guanylyltransferase [Ajellomyces dermatitidis SLH14081].   
169622087 SNOG_14258 413 Phaeosphaeria nodorum SN15 eukaryota>fungi>ascomycota hypothetical protein SNOG_14258 [Phaeosphaeria nodorum SN15].   
50552682 YALI0E09823g 309 Yarrowia lipolytica CLIB122 eukaryota>fungi>ascomycota YALI0E09823p [Yarrowia lipolytica].   
71003728 UM00383.1 714 Ustilago maydis 521 eukaryota>fungi>basidiomycota hypothetical protein UM00383.1 [Ustilago maydis 521].   
  
# 1;mispredicted as Cterminal only due to the pyrolysine being read as stop codon  
20089700 MA0816 92 Methanosarcina acetivorans C2A archaea>euryarchaeota hypothetical protein MA0816 [Methanosarcina acetivorans C2A].   
171913887 VspiD_010100021950 126 Verrucomicrobium spinosum DSM 4136 bacteria>chlamydiae/verrucomicrobia tRNAHis guanylyltransferase family protein [Verrucomicrobium   
196258004 Cyan7822DRAFT_3698 61 Cyanothece sp. PCC 7822 bacteria>cyanobacteria tRNAHis guanylyltransferase family protein [Cyanothece sp. PCC   
196256338 Cyan7822DRAFT_2033 123 Cyanothece sp. PCC 7822 bacteria>cyanobacteria tRNAHis guanylyltransferase family protein [Cyanothece sp. PCC   
163816591 COPEUT_02781 112 Coprococcus eutactus ATCC 27759 bacteria>firmicutes hypothetical protein COPEUT_02781 [Coprococcus eutactus ATCC   
295094970 CCU_28640 125 Coprococcus sp. ART55/1 bacteria>firmicutes Uncharacterized conserved protein [Coprococcus sp. ART55/1].   
52548693 GZ1tured archaeon GZfos18C8 archaea uncharacterized conserved protein [uncultured archaeon GZfos18C8].   
  
# 1;Fragment/Wrong start  
  
149052344 LOC303067 128 Rattus norvegicus eukaryota>metazoa>vertebrata similar to hypothetical protein FLJ20546, isoform CRA_c [Rattus   
270247845 GSVIVT01006301001 296 Vitis vinifera eukaryota>viridiplantae unnamed protein product [Vitis vinifera].   
52549238 GZ26E7_19 212 uncultured archaeon GZfos26E7 archaea uncharacterized conserved protein [uncultured archaeon GZfos26E7].   
196256820 Cyan7822DRAFT_2515 201 Cyanothece sp. PCC 7822 bacteria>cyanobacteria tRNAHis guanylyltransferase family protein [Cyanothece sp. PCC   
164659280 MGL_1763 225 Malassezia globosa CBS 7966 eukaryota>fungi>basidiomycota hypothetical protein MGL_1763 [Malassezia globosa CBS 7966].   
259488914 ANIA_00804 235 Aspergillus nidulans FGSC A4 eukaryota>fungi>ascomycota TPA: tRNAHis guanylyltransferase Thg1, putative (AFU_orthologue;   
110735837 At2g32330 205 Arabidopsis thaliana eukaryota>viridiplantae hypothetical protein [Arabidopsis thaliana].   
67607657 Chro.10228 164 Cryptosporidium hominis TU502 eukaryota>alveolata>apicomplexa hypothetical protein [Cryptosporidium hominis TU502].   
70928155 PC301576.00.0 221 Plasmodium chabaudi chabaudi eukaryota>alveolata>apicomplexa hypothetical protein [Plasmodium chabaudi chabaudi].   
240108585 CPC735_009350 225 Coccidioides posadasii C735 delta SOWgp eukaryota>fungi>ascomycota tRNAHis guanylyltransferase family protein [Coccidioides posadasii   
225561521 HCBG_01446 189 Ajellomyces capsulatus G186AR eukaryota>fungi>ascomycota tRNA guanylyltransferase [Ajellomyces capsulatus G186AR].   
194386006 - 166 Homo sapiens eukaryota>metazoa>vertebrata unnamed protein product [Homo sapiens].   
255606074 RCOM_1875060 185 Ricinus communis eukaryota>viridiplantae conserved hypothetical protein [Ricinus communis].   
218197168 OsI_20778 122 Oryza sativa Indica Group eukaryota>viridiplantae hypothetical protein OsI_20778 [Oryza sativa Indica Group].   
294464625 - 215 Picea sitchensis eukaryota>viridiplantae unknown [Picea sitchensis].   
242088615 SORBIDRAFT_09g026710 152 Sorghum bicolor eukaryota>viridiplantae hypothetical protein SORBIDRAFT_09g026710 [Sorghum bicolor].
```

  
Top  


---

Prokaryotic
Thg1 domain containing proteins with operons  

```
# 1;Thg1  
124027917 Thg1*-> Hbut_1048 238 Hyperthermus butylicus DSM 5456 archaea>crenarchaeota hypothetical protein Hbut_1048 [Hyperthermus butylicus DSM 5456]. 124027914_?->124027915_?-><-124027916_?||124027917_Thg1*->124027918_?->124027919_?->124027920_?->  
156937279 Thg1*-><-HTH Igni_0485 162 Ignicoccus hospitalis KIN4/I archaea>crenarchaeota hypothetical protein Igni_0485 [Ignicoccus hospitalis KIN4/I]. 156937276_?-><-156937277_?||156937278_?->156937279_Thg1*-><-156937280_HTH<-156937281_?||156937282_?->  
18312250 AAA_ATPase->carboxypeptidase_Taq-><-Thg1*||CTP_transf_2_phosphopantetheine_adenylyltransferase-><-peptidase_M28 PAE0886 212 Pyrobaculum aerophilum str. IM2 archaea>crenarchaeota hypothetical protein PAE0886 [Pyrobaculum aerophilum str. IM2]. 18312247_?->18312248_AAA_ATPase->18312249_carboxypeptidase_Taq-><-18312250_Thg1*||18312251_CTP_transf_2_phosphopantetheine_adenylyltransferase-><-18312252_peptidase_M28<-18312253_?  
145590370 AAA_ATPase->carboxypeptidase_Taq-><-Thg1*||CTP_transf_2_phosphopantetheine_adenylyltransferase-><-peptidase_M28 Pars_0105 196 Pyrobaculum arsenaticum DSM 13514 archaea>crenarchaeota hypothetical protein Pars_0105 [Pyrobaculum arsenaticum DSM 13514]. 145590367_?->145590368_AAA_ATPase->145590369_carboxypeptidase_Taq-><-145590370_Thg1*||145590371_CTP_transf_2_phosphopantetheine_adenylyltransferase-><-145590372_peptidase_M28<-145590373_?  
126458698 AAA_ATPase->carboxypeptidase_Taq-><-Thg1*||CTP_transf_2_phosphopantetheine_adenylyltransferase-><-peptidase_M28 Pcal_0070 225 Pyrobaculum calidifontis JCM 11548 archaea>crenarchaeota hypothetical protein Pcal_0070 [Pyrobaculum calidifontis JCM 126458695_?->126458696_AAA_ATPase->126458697_carboxypeptidase_Taq-><-126458698_Thg1*||126458699_CTP_transf_2_phosphopantetheine_adenylyltransferase-><-126458700_peptidase_M28<-126458701_?  
119872651 AAA_ATPase->carboxypeptidase_Taq-><-Thg1*||CTP_transf_2_phosphopantetheine_adenylyltransferase-><-peptidase_M28 Pisl_1144 212 Pyrobaculum islandicum DSM 4184 archaea>crenarchaeota hypothetical protein Pisl_1144 [Pyrobaculum islandicum DSM 4184]. 119872648_?->119872649_AAA_ATPase->119872650_carboxypeptidase_Taq-><-119872651_Thg1*||119872652_CTP_transf_2_phosphopantetheine_adenylyltransferase-><-119872653_peptidase_M28<-119872654_?  
171184582 AAA_ATPase->carboxypeptidase_Taq-><-Thg1*||CTP_transf_2_phosphopantetheine_adenylyltransferase-><-peptidase_M28 Tneu_0097 212 Thermoproteus neutrophilus V24Sta archaea>crenarchaeota hypothetical protein Tneu_0097 [Thermoproteus neutrophilus V24Sta]. 171184579_?->171184580_AAA_ATPase->171184581_carboxypeptidase_Taq-><-171184582_Thg1*||171184583_CTP_transf_2_phosphopantetheine_adenylyltransferase-><-171184584_peptidase_M28<-171184585_?  
  
154151809 isoleucyl-tRNA_synthetase-><-Thg1*<-PRC||S2P+CBS+CBS-><-RADICAL-SAM Mboo_2270 250 Candidatus Methanoregula boonei 6A8 archaea>euryarchaeota hypothetical protein Mboo_2270 [Candidatus Methanoregula boonei 154151806_?-><-154151807_?||154151808_isoleucyl-tRNA_synthetase-><-154151809_Thg1*<-154151810_PRC||154151811_S2P+CBS+CBS-><-154151812_RADICAL-SAM  
126178352 RADICAL-SAM-><-S2P+CBS+CBS||PRC->Thg1*-><-isoleucyl-tRNA_synthetase||Lysine_decarbox-> Memar_0402 244 Methanoculleus marisnigri JR1 archaea>euryarchaeota hypothetical protein Memar_0402 [Methanoculleus marisnigri JR1]. 126178349_RADICAL-SAM-><-126178350_S2P+CBS+CBS||126178351_PRC->126178352_Thg1*-><-126178353_isoleucyl-tRNA_synthetase||126178354_Lysine_decarbox-><-126178355_?  
219850835 RADICAL-SAM-><-S2P+CBS+CBS||PRC->Thg1*-><-isoleucyl-tRNA_synthetase||Lysine_decarbox-> Mpal_0151 243 Methanosphaerula palustris E1-9c archaea>euryarchaeota protein of unknown function DUF549 [Candidatus Methanosphaerula 219850832_RADICAL-SAM-><-219850833_S2P+CBS+CBS||219850834_PRC->219850835_Thg1*-><-219850836_isoleucyl-tRNA_synthetase||219850837_Lysine_decarbox-><-219850838_?  
88601907 isoleucyl-tRNA_synthetase->??30S_ribosomal_protein_S8??-><-Thg1*<-PRC||?->S2P+CBS+CBS-> Mhun_0610 241 Methanospirillum hungatei JF-1 archaea>euryarchaeota hypothetical protein Mhun_0610 [Methanospirillum hungatei JF-1]. 88601904_?->88601905_isoleucyl-tRNA_synthetase->88601906_??30S_ribosomal_protein_S8??-><-88601907_Thg1*<-88601908_PRC||88601909_?->88601910_S2P+CBS+CBS->  
82617278 <-glycyl-tRNA_synthetase<-4HB_Family_1_bi-partite_nucleotidyltransferase_subunit_DUF86<-Nucleotidyltransferase<-Thg1*<-PRC||S2P+CBS+CBS->THUMP+ThiI-> C4_0009 262 uncultured archaeon archaea conserved hypothetical protein [uncultured archaeon]. <-82617275_glycyl-tRNA_synthetase<-82617276_4HB_Family_1_bi-partite_nucleotidyltransferase_subunit_DUF86<-82617277_Nucleotidyltransferase<-82617278_Thg1*<-82617279_PRC||82617280_S2P+CBS+CBS->82617281_THUMP+ThiI->  
52548693 <-glycyl-tRNA_synthetase<-4HB_Family_1_bi-partite_nucleotidyltransferase_subunit_DUF86<-Nucleotidyltransferase<-Thg1_Fragment*<-PRC||S2P+CBS+CBS->THUMP+ThiI-> GZ18C8_19 212 uncultured archaeon GZfos18C8 archaea uncharacterized conserved protein [uncultured archaeon GZfos18C8]. <-52548690_glycyl-tRNA_synthetase<-52548691_4HB_Family_1_bi-partite_nucleotidyltransferase_subunit_DUF86<-52548692_Nucleotidyltransferase<-52548693_Thg1_Fragment*<-52548694_PRC||52548695_S2P+CBS+CBS->52548696_THUMP+ThiI->  
52549238 HTH-><-glycyl-tRNA_synthetase<-Nucleotidyltransferase<-Thg1_Fragment*<-PRC||S2P+CBS+CBS->THUMP+ThiI-> GZ26E7_19 212 uncultured archaeon GZfos26E7 archaea uncharacterized conserved protein [uncultured archaeon GZfos26E7]. 52549235_HTH-><-52549236_glycyl-tRNA_synthetase<-52549237_Nucleotidyltransferase<-52549238_Thg1_Fragment*<-52549239_PRC||52549240_S2P+CBS+CBS->52549241_THUMP+ThiI->  
268323173 RNase_P-><-S2P+CBS+CBS||PRC->Thg1*-> BSM_02380 253 uncultured archaeon archaea conserved hypothetical protein containing tRNAHis 268323170_RNase_P-><-268323171_S2P+CBS+CBS||268323172_PRC->268323173_Thg1*->268323174_?-><-268323175_?||268323176_?->  
284162827 <-Thg1*<-PRC||Ribosomal_L1->Ribosomal_L10-> Arcpr_1732 246 Archaeoglobus profundus DSM 5631 archaea>euryarchaeota protein of unknown function DUF549 [Archaeoglobus profundus DSM 284162824_?->284162825_?->284162826_?-><-284162827_Thg1*<-284162828_PRC||284162829_Ribosomal_L1->284162830_Ribosomal_L10->  
288559933 EMAP->PRC->aspartate_dehydrogenase->Thg1*->JAB-><-2-Hacid_dh+ACT thgL 252 Methanobrevibacter ruminantium M1 archaea>euryarchaeota tRNA(His) guanylyltransferase ThgL [Methanobrevibacter ruminantium 288559930_EMAP->288559931_PRC->288559932_aspartate_dehydrogenase->288559933_Thg1*->288559934_JAB-><-288559935_2-Hacid_dh+ACT||288559936_?->  
148642523 <-JAB<-Thg1*<-aspartate_dehydrogenase<-PRC||EMAP-> Msm_0463 239 Methanobrevibacter smithii ATCC 35061 archaea>euryarchaeota tRNA(His) guanylyltransferase [Methanobrevibacter smithii ATCC 148642520_?->148642521_?-><-148642522_JAB<-148642523_Thg1*<-148642524_aspartate_dehydrogenase<-148642525_PRC||148642526_EMAP->  
261349483 <-JAB<-Thg1*<-aspartate_dehydrogenase<-PRC||EMAP-> METSMIF1_02117 239 Methanobrevibacter smithii DSM 2374 archaea>euryarchaeota tRNAHis guanylyltransferase family protein [Methanobrevibacter 261349480_?-><-261349481_?<-261349482_JAB<-261349483_Thg1*<-261349484_aspartate_dehydrogenase<-261349485_PRC||261349486_EMAP->  
222445979 <-EMAP||PRC->aspartate_dehydrogenase->Thg1*->JAB-> METSMIALI_01627 239 Methanobrevibacter smithii DSM 2375 archaea>euryarchaeota hypothetical protein METSMIALI_01627 [Methanobrevibacter smithii <-222445976_EMAP||222445977_PRC->222445978_aspartate_dehydrogenase->222445979_Thg1*->222445980_JAB-><-222445981_?||222445982_?->  
282165226 <-S2P+CBS+CBS||PRC-><-MFS_transporter||Thg1*-><-Maltose_acetyltransferase MCP_2556 247 Methanocella paludicola SANAE archaea>euryarchaeota hypothetical protein MCP_2556 [Methanocella paludicola SANAE]. <-282165223_S2P+CBS+CBS||282165224_PRC-><-282165225_MFS_transporter||282165226_Thg1*-><-282165227_Maltose_acetyltransferase<-282165228_?||282165229_?->  
91773429 <-tyrosyl-tRNA_synthetase<-Thg1*<-PRC||DNA_PolI-> Mbur_1464 243 Methanococcoides burtonii DSM 6242 archaea>euryarchaeota hypothetical protein Mbur_1464 [Methanococcoides burtonii DSM <-91773426_?<-91773427_?<-91773428_tyrosyl-tRNA_synthetase<-91773429_Thg1*<-91773430_PRC||91773431_DNA_PolI->91773432_?->  
294496410 <-tyrosyl-tRNA_synthetase<-Thg1*<-PRC||DNA_PolI-> Mmah_1763 243 Methanohalophilus mahii DSM 5219 archaea>euryarchaeota tRNA(His)-5'-guanylyltransferase [Methanohalophilus mahii DSM 294496407_?-><-294496408_?<-294496409_tyrosyl-tRNA_synthetase<-294496410_Thg1*<-294496411_PRC||294496412_DNA_PolI->294496413_?->  
20089701 <-tyrosyl-tRNA_synthetase<-Thg1_C<-Thg1*<-PRC<-malate_dehydrogenase||DNA_PolI-> MA0817 141 Methanosarcina acetivorans C2A archaea>euryarchaeota hypothetical protein MA0817 [Methanosarcina acetivorans C2A]. <-20089698_?<-20089699_tyrosyl-tRNA_synthetase<-20089700_Thg1_C<-20089701_Thg1*<-20089702_PRC<-20089703_malate_dehydrogenase||20089704_DNA_PolI->  
20089700 <-tyrosyl-tRNA_synthetase<-Thg1_C*<-Thg1<-PRC<-malate_dehydrogenase MA0816 92 Methanosarcina acetivorans C2A archaea>euryarchaeota hypothetical protein MA0816 [Methanosarcina acetivorans C2A]. <-20089697_?<-20089698_?<-20089699_tyrosyl-tRNA_synthetase<-20089700_Thg1_C*<-20089701_Thg1<-20089702_PRC<-20089703_malate_dehydrogenase  
73669253 <-tyrosyl-tRNA_synthetase<-Thg1*<-PRC<-malate_dehydrogenase||DNA_PolI-> Mbar_A1746 243 Methanosarcina barkeri str. Fusaro archaea>euryarchaeota hypothetical protein Mbar_A1746 [Methanosarcina barkeri str. <-73669250_?<-73669251_?<-73669252_tyrosyl-tRNA_synthetase<-73669253_Thg1*<-73669254_PRC<-73669255_malate_dehydrogenase||73669256_DNA_PolI->  
21228066 <-tyrosyl-tRNA_synthetase<-Thg1*<-PRC<-malate_dehydrogenase||DNA_PolI-> MM_1964 243 Methanosarcina mazei Go1 archaea>euryarchaeota hypothetical protein MM_1964 [Methanosarcina mazei Go1]. <-21228063_?<-21228064_?<-21228065_tyrosyl-tRNA_synthetase<-21228066_Thg1*<-21228067_PRC<-21228068_malate_dehydrogenase||21228069_DNA_PolI->  
124485581 RADICAL-SAM-><-?||PRC->Thg1*->Thioredoxin->RNA-Helicase->TrmB_transcriptional_regulator-> Mlab_0759 240 Methanocorpusculum labreanum Z archaea>euryarchaeota hypothetical protein Mlab_0759 [Methanocorpusculum labreanum Z]. 124485578_RADICAL-SAM-><-124485579_?||124485580_PRC->124485581_Thg1*->124485582_Thioredoxin->124485583_RNA-Helicase->124485584_TrmB_transcriptional_regulator->  
20093533 <-Thg1*<-aspartate_dehydrogenase<-RNA_Me_trans<-PRC MK0093 262 Methanopyrus kandleri AV19 archaea>euryarchaeota hypothetical protein MK0093 [Methanopyrus kandleri AV19]. 20093530_?->20093531_?-><-20093532_?<-20093533_Thg1*<-20093534_aspartate_dehydrogenase<-20093535_RNA_Me_trans<-20093536_PRC  
116754185 <-Thg1*<-PRC Mthe_0875 235 Methanosaeta thermophila PT archaea>euryarchaeota hypothetical protein Mthe_0875 [Methanosaeta thermophila PT]. 116754182_?->116754183_?->116754184_?-><-116754185_Thg1*<-116754186_PRC||116754187_?->116754188_?->  
84489939 2-Hacid_dh+ACT-><-JAB<-Thg1*<-aspartate_dehydrogenase<-PRC||EMAP-> Msp_1147 237 Methanosphaera stadtmanae DSM 3091 archaea>euryarchaeota hypothetical protein Msp_1147 [Methanosphaera stadtmanae DSM 3091]. <-84489936_?||84489937_2-Hacid_dh+ACT-><-84489938_JAB<-84489939_Thg1*<-84489940_aspartate_dehydrogenase<-84489941_PRC||84489942_EMAP->  
15678990 2-Hacid_dh+ACT-><-JAB<-Thg1*<-aspartate_dehydrogenase<-PRC||EMAP-> MTH972 246 Methanothermobacter thermautotrophicus str. Delta H archaea>euryarchaeota hypothetical protein MTH972 [Methanothermobacter thermautotrophicus <-15678987_?||15678988_2-Hacid_dh+ACT-><-15678989_JAB<-15678990_Thg1*<-15678991_aspartate_dehydrogenase<-15678992_PRC||15678993_EMAP->  
147920979 <-TPR<-Thg1*||MFS_transporter-><-PRC||S2P+CBS+CBS-> RCIX455 241 uncultured methanogenic archaeon RC-I archaea>euryarchaeota hypothetical protein RCIX455 [uncultured methanogenic archaeon 147920976_?->147920977_?-><-147920978_TPR<-147920979_Thg1*||147920980_MFS_transporter-><-147920981_PRC||147920982_S2P+CBS+CBS->  
  
170290817 Thg1*-> Kcr_1204 249 Candidatus Korarchaeum cryptofilum OPF8 archaea>korarchaeota hypothetical protein Kcr_1204 [Candidatus Korarchaeum cryptofilum 170290814_?->170290815_?->170290816_?->170290817_Thg1*->170290818_?-><-170290819_?<-170290820_?  
219882632 Thg1*-> Achl_4028 238 Arthrobacter chlorophenolicus A6 bacteria>actinobacteria hypothetical protein Achl_4028 [Arthrobacter chlorophenolicus A6]. <-219882629_?||219882630_?->219882631_?->219882632_Thg1*-><-219882633_?<-219882634_?<-219882635_?  
252128517 <-Thg1* CORMA0001_0334 250 Corynebacterium matruchotii ATCC 14266 bacteria>actinobacteria trnahis guanylyltransferase family protein [Corynebacterium 252128514_?->252128515_?->252128516_?-><-252128517_Thg1*<-252128518_?||252128519_?->252128520_?->  
225022489 Thg1*-> CORMATOL_02529 250 Corynebacterium matruchotii ATCC 33806 bacteria>actinobacteria hypothetical protein CORMATOL_02529 [Corynebacterium matruchotii 225022486_?->225022487_?->225022488_?->225022489_Thg1*-><-225022490_?||225022491_?->225022492_?->  
54025916 <-Thg1* nfa39460 229 Nocardia farcinica IFM 10152 bacteria>actinobacteria hypothetical protein nfa39460 [Nocardia farcinica IFM 10152]. <-54025913_?||54025914_?-><-54025915_?<-54025916_Thg1*<-54025917_?<-54025918_?<-54025919_?  
 - -   
 - -   
254412407 <-Thg1* MC7420_6362 255 Microcoleus chthonoplastes PCC 7420 bacteria>cyanobacteria tRNAHis guanylyltransferase superfamily [Microcoleus chthonoplastes <-254412549_?||254412531_?-><-254412419_?<-254412407_Thg1*<-254412580_?||254412575_?->254412416_?->  
196256337 Thg1*->Thg1_C-> Cyan7822DRAFT_2032 141 Cyanothece sp. PCC 7822 bacteria>cyanobacteria tRNAHis guanylyltransferase family protein [Cyanothece sp. PCC 196256334_?-><-196256335_?||196256336_?->196256337_Thg1*->196256338_Thg1_C-><-196256339_?<-196256340_?  
228982997 Thg1*-> bthur0002_61310 353 Bacillus thuringiensis Bt407 bacteria>firmicutes hypothetical protein bthur0002_61310 [Bacillus thuringiensis <-228982994_?||228982995_?-><-228982996_?||228982997_Thg1*->228982998_?->228982999_?->228983000_?->  
75758411 <-Thg1* RBTH_06728 245 Bacillus thuringiensis serovar israelensis ATCC 35646 bacteria>firmicutes Hypothetical protein RBTH_06728 [Bacillus thuringiensis serovar 75758408_?-><-75758409_?<-75758410_?<-75758411_Thg1*<-75758412_?<-75758413_?<-75758414_?  
291529569 <-Thg1* ERE_34100 285 Eubacterium rectale M104/1 bacteria>firmicutes Uncharacterized conserved protein [Eubacterium rectale M104/1]. <-291529566_?<-291529567_?<-291529568_?<-291529569_Thg1*<-291529570_?<-291529571_?<-291529572_?  
168698293 <-Thg1* GobsU_010100002157 279 Gemmata obscuriglobus UQM 2246 bacteria>planctomycetes hypothetical protein GobsU_02157 [Gemmata obscuriglobus UQM 2246]. <-168698290_?<-168698291_?||168698292_?-><-168698293_Thg1*||168698294_?-><-168698295_?<-168698296_?  
153872919 Thg1*-> BGP_4908 214 Beggiatoa sp. PS bacteria>proteobacteria>gammaproteobacteria protein of unknown function DUF549 [Beggiatoa sp. PS]. 153872919_Thg1*-><-153872920_?  
 - -   
225075026 Thg1*->transcriptional_regulator_RtcR-> NEIFLAOT_00025 252 Neisseria flavescens NRL30031/H210 bacteria>proteobacteria>betaproteobacteria hypothetical protein NEIFLAOT_00025 [Neisseria flavescens 225075023_?->225075024_?-><-225075025_?||225075026_Thg1*->225075027_transcriptional_regulator_RtcR-><-225075028_?<-225075029_?  
294671196 Thg1*->transcriptional_regulator_RtcR->Lysyl-tRNA_synthetase-> NEIELOOT_02904 255 Neisseria elongata subsp. glycolytica ATCC 29315 bacteria>proteobacteria>betaproteobacteria hypothetical protein NEIELOOT_02904 [Neisseria elongata subsp. 294671193_?-><-294671195_?||294671194_?->294671196_Thg1*->294671197_transcriptional_regulator_RtcR->294671198_Lysyl-tRNA_synthetase-><-294671199_?  
255067726 <-transcriptional_regulator_RtcR<-Thg1* NEISICOT_02755 251 Neisseria sicca ATCC 29256 bacteria>proteobacteria>betaproteobacteria tRNA(His) guanylyltransferase family protein [Neisseria sicca ATCC 255067723_?-><-255067724_?<-255067725_transcriptional_regulator_RtcR<-255067726_Thg1*||255067727_?->255067728_?->255067729_?->  
 - -   
258545199 <-transcriptional_regulator_RtcR<-false<-DNA_alkylation<-Thg1*<-Poly-Nucleotide--kinase HMPREF0198_1468 256 Cardiobacterium hominis ATCC 15826 bacteria>proteobacteria>gammaproteobacteria tRNAHis guanylyltransferase family protein [Cardiobacterium hominis <-258545196_transcriptional_regulator_RtcR<-258545197_false<-258545198_DNA_alkylation<-258545199_Thg1*<-258545200_Poly-Nucleotide--kinase||258545201_?->258545202_?->  
256424765 Poly-Nucleotide--kinase->Thg1*-> Cpin_5797 255 Chitinophaga pinensis DSM 2588 bacteria>bacteroidetes/chlorobi tRNA(His)-5'-guanylyltransferase [Chitinophaga pinensis DSM 2588]. <-256424762_?||256424763_?->256424764_Poly-Nucleotide--kinase->256424765_Thg1*-><-256424766_?<-256424767_?||256424768_?->  
124008957 <-Thg1*<-DinB_Hydrolase<-Poly-Nucleotide--kinase M23134_06676 250 Microscilla marina ATCC 23134 bacteria>bacteroidetes/chlorobi conserved protein [Microscilla marina ATCC 23134]. 124008954_?->124008955_?->124008956_?-><-124008957_Thg1*<-124008958_DinB_Hydrolase<-124008959_Poly-Nucleotide--kinase<-124008960_?  
171913887 Poly-Nucleotide--kinase->Thg1->?->Thg1_C*-> VspiD_010100021950 126 Verrucomicrobium spinosum DSM 4136 bacteria>chlamydiae/verrucomicrobia tRNAHis guanylyltransferase family protein [Verrucomicrobium 171913884_Poly-Nucleotide--kinase->171913885_Thg1->171913886_?->171913887_Thg1_C*-><-171913888_?||171913889_?->171913890_?->  
171913885 MACRODOMAIN->ADP-ribosylglycohydrolase->Poly-Nucleotide--kinase->Thg1*->?->Thg1_C-> VspiD_010100021940 159 Verrucomicrobium spinosum DSM 4136 bacteria>chlamydiae/verrucomicrobia tRNAHis guanylyltransferase family protein [Verrucomicrobium 171913882_MACRODOMAIN->171913883_ADP-ribosylglycohydrolase->171913884_Poly-Nucleotide--kinase->171913885_Thg1*->171913886_?->171913887_Thg1_C-><-171913888_?  
159900562 <-Thg1*<-Poly-Nucleotide--kinase Haur_4049 252 Herpetosiphon aurantiacus ATCC 23779 bacteria>chloroflexi hypothetical protein Haur_4049 [Herpetosiphon aurantiacus ATCC 159900559_?->159900560_?-><-159900561_?<-159900562_Thg1*<-159900563_Poly-Nucleotide--kinase||159900564_?->159900565_?->  
158336895 Poly-Nucleotide--kinase->Thg1*-> AM1_3767 253 Acaryochloris marina MBIC11017 bacteria>cyanobacteria tRNAHis guanylyltransferase family protein [Acaryochloris marina 158336892_?->158336893_?->158336894_Poly-Nucleotide--kinase->158336895_Thg1*-><-158336896_?<-158336897_?<-158336898_?  
269120695 Thg1*->Poly-Nucleotide--kinase-> Sterm_2087 241 Sebaldella termitidis ATCC 33386 bacteria>fusobacteria hypothetical protein Sterm_2087 [Sebaldella termitidis ATCC 33386]. 269120692_?->269120693_?->269120694_?->269120695_Thg1*->269120696_Poly-Nucleotide--kinase->269120697_?->269120698_?->  
229539832 <-Thg1*<-Poly-Nucleotide--kinase PlimDRAFT_36970 258 Planctomyces limnophilus DSM 3776 bacteria>planctomycetes tRNA(His)-5'-guanylyltransferase [Planctomyces limnophilus DSM <-229539829_?<-229539830_?<-229539831_?<-229539832_Thg1*<-229539833_Poly-Nucleotide--kinase<-229539834_?||229539835_?->  
162454339 Poly-Nucleotide--kinase->Thg1*-> sce6062 248 Sorangium cellulosum 'So ce 56' bacteria>proteobacteria>deltaproteobacteria hypothetical protein sce6062 [Sorangium cellulosum 'So ce 56']. 162454336_?->162454337_?->162454338_Poly-Nucleotide--kinase->162454339_Thg1*->162454340_?->162454341_?->162454342_?->  
149920560 Poly-Nucleotide--kinase->Thg1*-> PPSIR1_23574 274 Plesiocystis pacifica SIR-1 bacteria>proteobacteria>deltaproteobacteria hypothetical protein PPSIR1_23574 [Plesiocystis pacifica SIR-1]. 149920557_?->149920558_?->149920559_Poly-Nucleotide--kinase->149920560_Thg1*-><-149920561_?||149920562_?->149920563_?->  
108762077 <-Thg1*<-Poly-Nucleotide--kinase MXAN_5968 267 Myxococcus xanthus DK 1622 bacteria>proteobacteria>deltaproteobacteria tRNAHis guanylyltransferase family protein [Myxococcus xanthus DK 108756771_?->108758036_?->108761385_?-><-108762077_Thg1*<-108763390_Poly-Nucleotide--kinase||108762242_?-><-108761245_?  
 - -   
 - -   
163816591 RNA_ligase->Thg1->Thg1_C*->MACRODOMAIN-> COPEUT_02781 112 Coprococcus eutactus ATCC 27759 bacteria>firmicutes hypothetical protein COPEUT_02781 [Coprococcus eutactus ATCC 163816588_?->163816589_RNA_ligase->163816590_Thg1->163816591_Thg1_C*->163816592_MACRODOMAIN->163816593_?->163816594_?->  
163816590 <-HD||?->RNA_ligase->Thg1*->Thg1_C->MACRODOMAIN-> COPEUT_02780 128 Coprococcus eutactus ATCC 27759 bacteria>firmicutes hypothetical protein COPEUT_02780 [Coprococcus eutactus ATCC <-163816587_HD||163816588_?->163816589_RNA_ligase->163816590_Thg1*->163816591_Thg1_C->163816592_MACRODOMAIN->163816593_?->  
295094970 <-MACRODOMAIN<-Thg1_C* CCU_28640 125 Coprococcus sp. ART55/1 bacteria>firmicutes Uncharacterized conserved protein [Coprococcus sp. ART55/1]. <-295094967_?<-295094968_?<-295094969_MACRODOMAIN<-295094970_Thg1_C*<-295094971_?<-295094972_?<-295094973_?  
 - -   
# 1; Thg1  
288922849 Thg1*-> FrEUN1fDRAFT_6712 247 Frankia sp. EUN1f bacteria>actinobacteria hypothetical protein FrEUN1fDRAFT_6712 [Frankia sp. EUN1f]. 288922846_?->288922847_?->288922848_?->288922849_Thg1*-><-288922850_?||288922851_?->288922852_?->  
149908211 <-S1COLD<-Thg1* PE36_01857 288 Moritella sp. PE36 bacteria>proteobacteria>gammaproteobacteria hypothetical protein PE36_01857 [Moritella sp. PE36]. <-149908208_?<-149908209_?<-149908210_S1COLD<-149908211_Thg1*<-149908212_?<-149908213_?<-149908214_?  
 - -   
196258004 Thg1_C*-> Cyan7822DRAFT_3698 61 Cyanothece sp. PCC 7822 bacteria>cyanobacteria tRNAHis guanylyltransferase family protein [Cyanothece sp. PCC 196258001_?->196258002_?->196258003_?->196258004_Thg1_C*-><-196258005_?<-196258006_?<-196258007_?  
196256338 Thg1->Thg1_C*-> Cyan7822DRAFT_2033 123 Cyanothece sp. PCC 7822 bacteria>cyanobacteria tRNAHis guanylyltransferase family protein [Cyanothece sp. PCC <-196256335_?||196256336_?->196256337_Thg1->196256338_Thg1_C*-><-196256339_?<-196256340_?||196256341_?->  
# 1;Thg1_Frag  
196256820 <-Thg1_Fragment*<-Thg1 Cyan7822DRAFT_2515 201 Cyanothece sp. PCC 7822 bacteria>cyanobacteria tRNAHis guanylyltransferase family protein [Cyanothece sp. PCC <-196256817_?||196256818_?->196256819_?-><-196256820_Thg1_Fragment*<-196256821_Thg1<-196256822_?||196256823_?->
```

Top  


---

Phylogenetic
tree of Thg1 domain containing proteins  
  
The tree was constructed with the Thg1 RRM-Palm domain alone (without
the C terminal extension)  
Archaea - Blue  
Bacteria - Violet  
Eukaryotes: Plants- Green; Animals- Red; Fungi- Grey; Apicomplexans-
Brown; Amoebozoans/ bacterial transfers to eukaryotes- Ochre; Rest-
Black  
  

  
Top
